# Solvation Entropy as a Lever for Steering the Macroscopic Properties of a Functional Supramolecular Helical Polymer

**DOI:** 10.1002/anie.202521365

**Published:** 2025-11-20

**Authors:** Huanjun Kong, Mayte A. Martínez‐Aguirre, Yan Li, Tomoyuki Ikai, Eiji Yashima, Gangamallaiah Velpula, Steven De Feyter, Pierre‐Antoine Albouy, Komivi Akpo, Patrick Brocorens, Roberto Lazzaroni, Laurent Bouteiller, Matthieu Raynal

**Affiliations:** ^1^ Sorbonne Université CNRS Institut Parisien de Chimie Moléculaire 4 Place Jussieu Paris 75005 France; ^2^ Department of Molecular and Macromolecular Chemistry, Graduate, School of Engineering Nagoya University Chikusa‐ku, Nagoya 464‐8603 Japan; ^3^ Division of Molecular Imaging and Photonics Department of Chemistry KU Leuven Celestijnenlaan 200F Leuven B 3001 Belgium; ^4^ Laboratoire de Physique des Solides CNRS Université Paris‐Sud Université Paris‐Saclay Orsay 91400 France; ^5^ Service de Chimie des Matériaux Nouveaux Institut de Recherche sur les Matériaux Université de Mons Place du Parc, 20 Mons B‐7000 Belgium; ^6^ Present address: Department of Chemical Engineering National Tsing Hua University 101, Sec. 2, Kuang‐Fu Road Hsinchu 30013 Taiwan (R.O.C.)

**Keywords:** Hydrogen bonding, Solvation, Structural transition, Supramolecular chirality, Supramolecular polymer, Thermothickening

## Abstract

Solvent‐solute interactions are utterly important in supramolecular polymers (SPs), yet the high responsivity of SPs to solvent polarity makes it challenging to play on other solvation effects to tune their macroscopic properties in a rational manner. Herein, we report the characterization at various scales of the assembly properties of a *C*_2_‐symmetric benzene‐1,3,5‐tricarboxamide monomer with two (1*S*)‐methylheptyl moieties and one diphenylphosphino group as lateral chains. Our investigation reveals a highly cooperative structural transition between two SP states, which can exquisitely be tuned by solvents of similar polarities, leading to variation of the transition temperature (T*) over a range of 85 K. The structural transition was detected in 4 pure solvents and 13 toluene/cosolvent mixtures; a fair relationship is determined between T* and the solvent molar volume. The transition is only weakly favored by enthalpy (by ca. 2 kJ.mol^−1^ at 286 K). However, minimization of the entropic cost leads to a notable increase in the T*. This allows a fine tuning of the thermothickening and catalytic properties of the resulting SPs auguring that solvation, and notably solvation entropy, may constitute an important lever for steering SPs structure and properties.

## Graphical abstract

The structural transition between the two fully‐characterized states of a supramolecular helical hydrogen‐bonded polymer (represented as blue and light pink stacks) can be predictably varied over 85 K by changing the nature and amount of cosolvent; entropy of solvation (schematized as solvents of different sizes) is the main lever to tune the macroscopic properties of the resulting polymers.

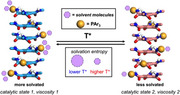

## Introduction

Solvation, the ability of solvent molecules to interact with solutes, is a fundamental process in chemical sciences that controls chemical equilibria,^[^ ^1^ ^]^ reaction rates^[^ ^2^ ^]^ and the conformation of discrete molecules,^[^ ^3^ ^]^ macromolecules^[^ ^4^ ^]^ and assemblies.^[^ ^5^ ^]^ Non‐covalent interactions are particularly sensitive to their environment and, as such, the construction of supramolecular architectures relies on a delicate balance between solute‐solute, solute‐solvent and solvent‐solvent interactions. In particular, solvent polarity, as quantified by a variety of empirical and model‐based parameters, affords a good rationalization of the strength of a variety of supramolecular complexes.^[^ ^5^ ^]^ In this regard, supramolecular polymers (SPs),^[^ ^6^, ^7^ ^]^ formed through the association of small molecules by means of non‐covalent interactions, constitute an exceptional playground to probe solvation effects because of their dynamic and macromolecular nature. Even though a precise picture of solvation at the (sub)molecular level is still lacking,^[^ ^8^ ^]^ the global effect of solvents on their ability to promote the association or dissociation of complementary monomers has gained significant understanding in recent years. Good solvents are notably used to dissociate SPs, in a way similar but not identical to protein denaturation, allowing systematic evaluation of the parameters governing the SP (dis)assembly.^[^ ^9^, ^10^, ^11^, ^12^ ^]^ Solvent quality is also used as a tool to mitigate assembly kinetics^[^ ^9^ ^]^ and to exert control on the nature and structure of SPs formed in competition through complex aggregation pathways.^[^ ^13^, ^14^, ^15^, ^16^, ^17^, ^18^, ^19^ ^]^ Likewise, the formation of supramolecular gels^[^ ^20^, ^21^ ^]^ and polymers^[^ ^22^ ^]^ is well related to solvent solubility parameters which offer a predictive tool for assembly formation. Monomers with elaborated molecular structures may assemble into various morphologies in solvent or solvent mixtures of different polarities; this has been exploited to tame the conductive,^[^ ^23^ ^]^ photophysical,^[^ ^24^, ^25^ ^]^ and chiroptical^[^ ^14^, ^26^, ^27^, ^28^, ^29^, ^30^, ^31^, ^32^, ^33^, ^34^, ^35^, ^36^, ^37^ ^]^ properties of a variety of SPs, including SPs adopting helical conformations. However, fine tuning of the SP conformations by playing on the nature of solvent and/or the composition of solvent mixtures, without relying on a drastic change in the SP structure/morphology would undoubtedly be useful for steering their macroscopic properties in a predictable manner.

Hydrogen‐bonded SPs adopting a dynamic helical conformation are particularly suited to achieve this goal because of their ability to adapt their structure to even minute perturbations of their environment, notably those induced by additives or solvents of similar polarity.^[^ ^38^, ^39^, ^40^ ^]^ Water molecules have recently been found to be non‐innocent components acting as comonomers in apolar solvents and leading to inversion of supramolecular helicity^[^ ^41^, ^42^, ^43^ ^]^ or capping^[^ ^43^, ^44^ ^]^ of the SP chains. In a relatively similar fashion,^[^ ^45^, ^46^, ^47^ ^]^ the relative stabilities of monomer‐monomer, monomer‐alcohol and alcohol‐alcohol aggregates have been finely tuned to yield diluted‐ and temperature‐induced polymerization processes,^[^ ^46^, ^47^ ^]^ unusual phenomena for SPs in apolar solutions. In these examples, interaction of the monomers with the additives through hydrogen‐bonding interactions has an obvious *enthalpic* contribution. SPs were also shown to adapt their conformations in the presence of solvents lacking obvious competing groups. Induction of a preferential supramolecular helicity by means of chiral solvents presumably occurs through specific solvation of the SP helical side chain(s)^[^ ^48^, ^49^, ^50^ ^]^ or nanotube interior.^[^ ^51^ ^]^ Likewise, changes in SP morphology,^[^ ^52^, ^53^, ^54^ ^]^ helical handedness,^[^ ^55^, ^56^, ^57^, ^58^ ^]^ extent of helicity,^[^ ^59^ ^]^ stability,^[^ ^60^, ^61^, ^62^, ^63^ ^]^ dynamicity,^[^ ^64^ ^]^ and cooperativity^[^ ^65^, ^66^ ^]^ of the aggregation pathway have been reported which are likely related to subtle modification in the solvation shell surrounding the SP side chains. The coexistence of two helical SPs in competition proves to be advantageous in the context of probing subtle changes in the solvation process at the condition that it translates into an easily detectable transition between the two states.^[^ ^67^, ^68^, ^69^, ^70^, ^71^ ^]^ Bouteiller and van der Schoot determined that the stability of bisurea based nanotubes was influenced by the nature of contacts between solvents confined in the interior of the tubes even in the case of solvents of similar polarities.^[^ ^62^ ^]^ Meijer and coworkers correlated the sudden inversion of SP helicity upon cooling a solution of a chiral triphenylene‐2,6,10‐tricarboxamide monomer to the solvation of its side chain by the chiral solvent.^[^ ^50^ ^]^ The inversion is enthalpically favorable but the enthalpy change is modest (Δ*H* = 3 kJ.mol^−1^); variation of the transition temperature with cosolvent further supported that the “differential solvation of the various helical states of the SPs by the chiral solvent is of an entropic nature”. Solvent penetration, intercalation and sequestration as mentioned in the preceding literature examples are solvation phenomena that are irrefutably mediated by a strong *entropic* contribution. Whilst it can thus be reasonably envisaged that playing on the relative extent of the enthalpic and entropic contributions of the solvation of SPs can be used to modulate their structures, there is a need to better apprehend what are the molecular requirements necessary to observe differentially solvated states of SPs and how this could translate into tunable macroscopic properties.

**Scheme 1:**
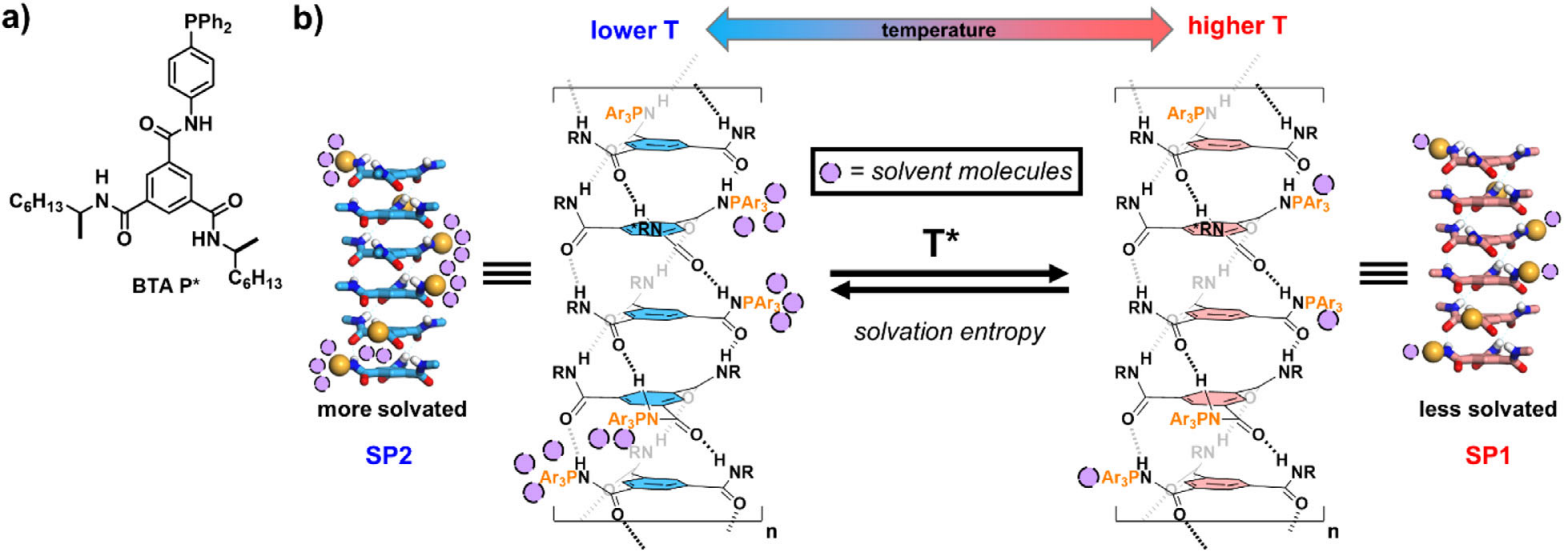
a) Chemical structure of the BTA monomer investigated in this study. b) Schematic representation of the two states (SP1 and SP2) of supramolecular polymers formed by BTA P* whose transition occurs sharply at a given temperature (T*) which is modulated by the differential solvation of the lateral PAr_3_ groups in the two states, mostly by leveraging solvation entropy. Solvent molecules are schematically represented as light purple spheres.

In our continuing efforts to develop benzene‐1,3,5‐tricarboxamide SPs as a platform for catalysis,^[^ ^72^, ^73^, ^74^, ^75^, ^76^, ^77^, ^78^, ^79^ ^]^ we discovered the ability of a structurally‐simple *C*_2_‐symmetric BTA derivative (BTA P*, Scheme 1a) to assemble into two distinct SPs as a function of the temperature; a process that we have thoroughly investigated by multifarious analytical techniques. The highly cooperative transition between the two states, which has a very weak favorable enthalpy (Δ*H* ≈ 2 kJ.mol^−1^), can be exquisitely tuned by solvents of similar polarity, leading to variation of the transition temperature over a range of ca. 85 K (Scheme 1b). The correlation of the transition temperature (mostly) with solvent molecular volumes and the comparison of the differential free energies of the SPs in different solvents unambiguously ascertain that solvation entropy is the major lever for tuning the relative stability of the two states. Control tests with selected model compounds relate the observed effect to the temperature‐dependent solvation of the lateral diphenylphosphino group. Consequently, the catalytic and rheological properties of the chiral SPs are steered rationally paving the way for improved properties of functional supramolecular materials by playing on solvation rather than by frequently‐used modification of the monomer structure, action of additives or tuning of the aggregation pathway.

## Results and Discussion

### Supramolecular Polymerization of BTA P*

Our group previously investigated chiral and achiral BTA ligands in asymmetric catalysis for which selectivity stemmed exclusively from their ability to organize into supramolecular helices (see a schematic representation in Scheme 1b).^[^ ^72^, ^73^, ^74^, ^75^, ^76^, ^77^, ^78^, ^79^ ^]^ During the course of our study, we have prepared BTA P*, a *C*_2_‐symmetric BTA monomer which combines two different types of lateral chain: one consists of a diphenylphosphino group (as metal binding group) appended to the BTA ring through a 1,4‐phenylene linker and the other one is a (1*S*)‐methylheptyl moiety for chirality induction. BTA P* was prepared straightforwardly in an enantiopure form (see the Supporting Information). BTA P* proves to be soluble in a range of aromatic solvents but its solubility is limited in alkanes (Table S1).

**Figure 1:**
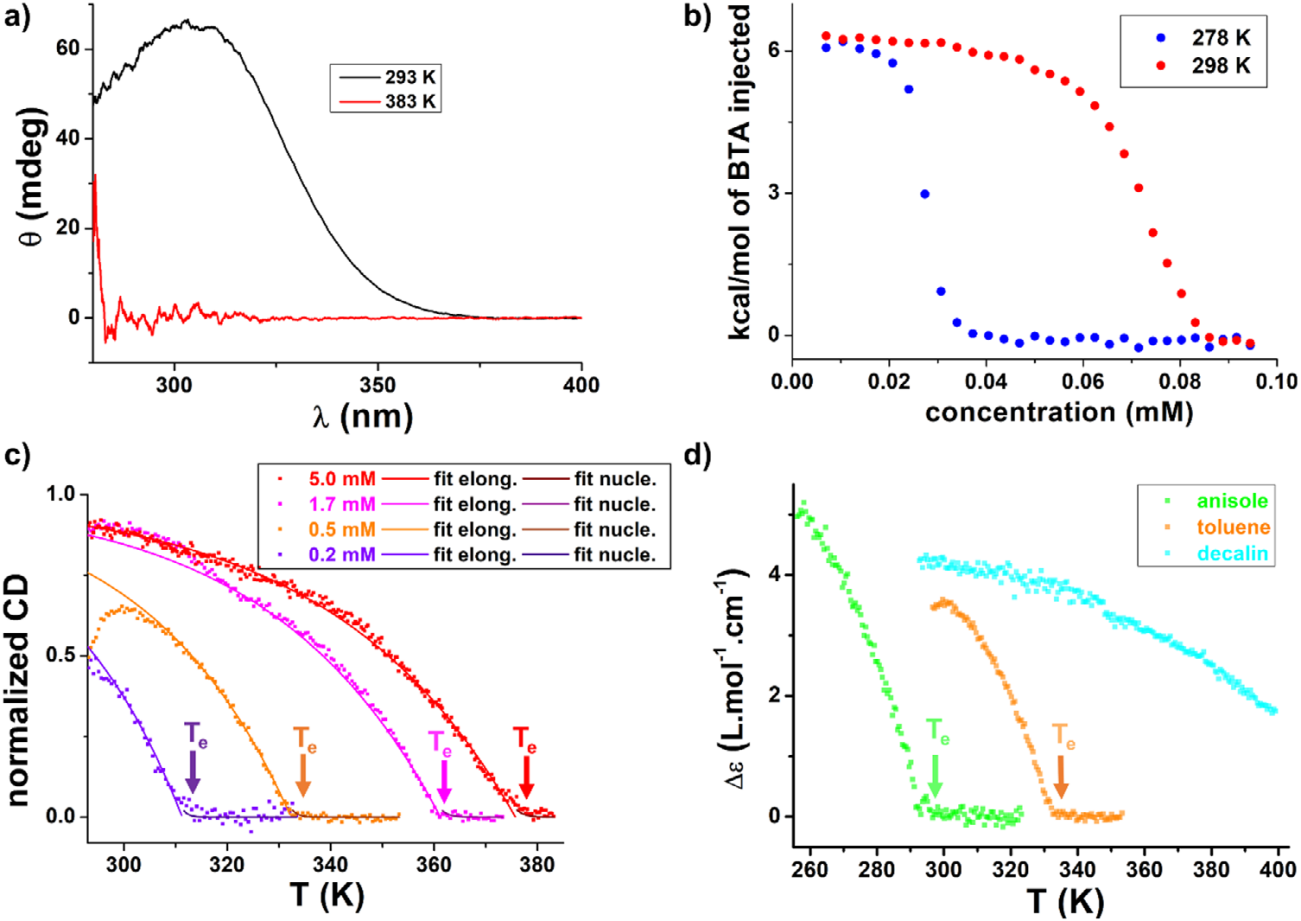
a) Variable‐temperature CD analyses of a 2.0 mM solution of BTA P* in toluene at 293 K and 383 K (spectra were obtained upon cooling, 1 K.min^−1^). b) ITC data obtained for toluene solutions containing BTA P* (0.5 mM) injected into pure toluene, versus total BTA concentration in the cell at 278 and 298 K. Critical concentrations (c*) are extracted that correspond to twice the concentration at the mid‐point of the heatflow jump,^[^ ^80^, ^81^ ^],^ i.e., c* = 0.06 mM and 0.14 mM, at 278 K and 298 K, respectively. c) CD intensity (*λ* = 330 nm) as a function of the temperature for solutions of BTA P* at different concentrations in toluene. Data recorded upon cooling (0.5 K.min^−1^). The curves have been fitted conjointly with the nucleation–growth model^[^ ^60^ ^]^ which requires both nucleation and elongation regimes to be fitted by two independent equations. Very good fits were obtained with *K*_a_ = 2 × 10^−5^ and *h*_e _= 8 kcal.mol^−1^ which allow to extract the following elongation temperatures: *T*_e_ = 311.3 K (0.2 mM), *T*_e_ = 332.4 K (0.5 mM), *T*_e_ = 361.2 K (1.7 mM), *T*_e_ = 375.6 K (5.0 mM). The data below 293 K have been omitted on purpose (see Figure 2b,c). For the same plot with CD values in Δ*ε* (L.mol^−1^.cm^−1^), see Figure S1c,d) CD intensity (*λ* = 330 nm) as a function of the temperature for 0.5 mM solutions of BTA P* in toluene, anisole and decalin (cooling, 0.5 K.min^−1^).

The analytical data of the SP formed by BTA P* in toluene at 293 K are characteristic of hydrogen‐bonded helical stacks as demonstrated by diagnostic Fourier‐Transform InfraRed (FT‐IR, Figure S1a) signals and Small‐Angle Neutron Scattering (SANS, Figure S1b) curve.^[^ ^82^, ^83^ ^]^ Likewise, the low‐energy Circular Dichroism (CD) band with a maximum at 306 nm is related to the induction of chirality from the supramolecular helices to the diphenylphosphino group;^[^ ^72^, ^73^, ^74^, ^75^, ^76^, ^77^, ^78^, ^79^ ^]^ which thus vanishes when the assemblies are disrupted at higher temperature (Figure 1a). The disassembly process was more precisely studied by Isothermal Titration Calorimetry (ITC) and variable‐temperature (VT) CD analyses. Integration of the endothermal signals obtained by ITC upon diluting solutions of BTA P* yields the ITC data shown in Figure 1b. The value of the dissociation enthalpy (≈ 6 kcal.mol^−1^) and the fact that the critical concentration increases with the temperature are characteristic of hydrogen‐bonded BTA assemblies.^[^ ^79^ ^]^ For solutions of BTA P* above 0.1 mM, the disassembly process can be conveniently monitored by VT‐CD analyses. The CD intensity at 330 nm shows an abrupt change at a temperature which increases as a function of the concentration (Figure 1c). The CD cooling curves can be appropriately fitted by the nucleation–growth model developed by van der Schoot and co‐workers^[^ ^60^ ^]^ yielding a dimensionless equilibrium constant, *K*_a_ (activation of the monomer), and the enthalpy release upon elongation (*h*_e_) at the elongation temperature (*T*_e_). These values are compiled in the caption of Figure 1. The *K*_a_ value of 2 × 10^−5^ is consistent with a highly cooperative aggregation pathway. The value of the elongation enthalpy (8 kcal.mol^−1^) is of the same order as the enthalpy change measured by ITC (6 kcal.mol^−1^) and the enthalpy extracted from the Van't Hoff plot (9 kcal.mol^−1^, Figure S1d), indicating that the disassembly process is robustly probed over a large range of concentrations and temperatures by different techniques. These values are consistent with those reported in the literature for other *C*_3_‐ and *C*_2_‐symmetrical BTA SPs in toluene.^[^ ^79^ ^]^

We next probed the influence of the solvent on the thermodynamics of the dissociation process (Figure 1d). The stability of the SP formed by BTA P* increases in the following order: anisole (*E_T_* 30 = 37.1) < toluene (*E_T_* 30 = 33.9) < decalin (*E_T_* 30 = 31.2) with a difference in stability of more than 100 K as reflected by the values of the elongation temperatures. The trend is consistent with a higher stability provided by the less polar solvents, as expected for a SP based on hydrogen‐bonding and aromatic interactions.^[^ ^8^ ^]^ The supramolecular polymerization of BTA P* monomers into helical stacks is thus conventional and follows the expected responsivity to solvent polarity.

### Transition Between the Two SP States in Toluene

**Figure 2:**
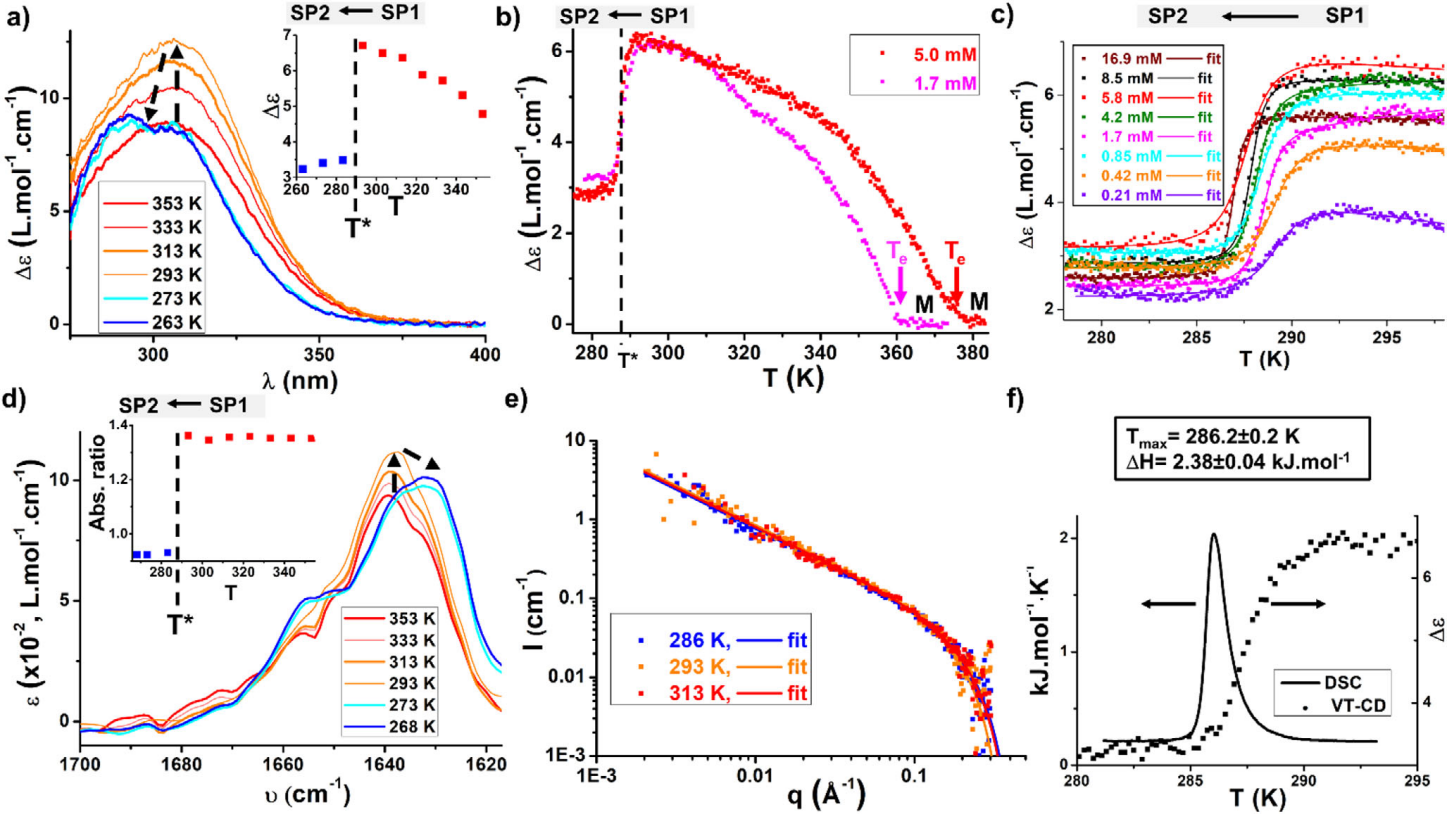
a) CD spectra of an 8.5 mM solution of BTA P* in toluene recorded at different temperatures (353 K to 263 K, 1 K.min^−1^). Arrows help to visualize the evolution of the CD bands when the temperature decreases. For CD and UV–vis spectra recorded every 10 K, see Figure S3. Insert: plot of the molar CD values at 330 nm as a function of the temperature. b) CD intensity (*λ* = 330 nm) as a function of the temperature for solutions of BTA P* at two different concentrations in toluene. Data recorded upon cooling (0.5 K.min^−1^). c) CD intensity (*λ* = 330 nm) as a function of the temperature for solutions of BTA P* at different concentrations in toluene. Data recorded upon cooling (0.1 K.min^−1^). The temperature range (278 K–298 K) was selected to tightly encompass the structural transition. The cooling curves for all solutions were fitted with the model for structural transitions occurring in linear assemblies, developed by van der Schoot et al.^[^ ^84^, ^85^ ^]^ Assuming a transition enthalpy of 2.38 kJ.mol^−1^, the transition temperatures and cooperativity factors were extracted for each concentration and compiled in Figure S6. d) FT‐IR spectra of an 8.5 mM solution of BTA P* in toluene recorded at different temperatures (353 K to 268 K, recorded upon heating, 1 K.min^−1^). Zoom on the amide I C═O band. Arrows help to visualize the evolution of the FT‐IR bands when the temperature decreases. Insert: plot of the ratio of ε values at 1638 and 1630 cm^1^ as a function of the temperature. e) SANS analyses of an 8.7 mM (6.0 g.L^−1^) solution of BTA P* in toluene‐d_8_ at 286 K, 293 K and 313 K with the corresponding fits for rigid rods. The extracted radii and number of molecules in the cross‐section are indicated in Figure S7. f) High‐sensitivity DSC analysis of a 10.0 mM solution of BTA P* in toluene obtained upon heating (0.2 K.min^−1^). The transition temperature corresponds to the abscissa at the maximum of the endothermal peak whilst the transition enthalpy is obtained upon integration of the peak. Comparison with the CD data obtained in the same temperature range.

Below 293 K, a sudden change in the spectroscopic data recorded for solutions of BTA P* occurs in toluene (or toluene‐d_8_). Two SP states are observed below and above 288 K that are characterized by small but significant shifts of the low‐energy CD and UV–vis bands (belonging to the PAr_3_ moiety) and of the amide C═O stretching frequency, and by a broadening of the ^31^P NMR chemical shift (see Figure S2 for the normalized spectra). More precisely, the CD signal experiences a decrease in its intensity accompanied by a change of its shape from a broad single band above 293 K to a sharper band with two detectable bumps at 283 K and below (Figure 2a). The change in the shape and intensity of the CD band allows us to precisely probe the transition. Monitoring of the CD intensity at 330 nm from 383 to 278 K shows first the assembly of BTA P* monomers into the first type of SP (SP1) at the elongation temperature (*T*_e_) and then the transition between the two SP states (Figure 2b) at the transition temperature (T*). The transition is actually very sharp, occurring within a temperature range of only 5 K (Figure 2c). The CD curves can be perfectly fitted by the two‐state model for the transitions occurring in linear self‐assemblies reported by van der Schoot and coworkers^[^ ^84^, ^85^ ^]^ yielding a T* value of 288 ± 1.1 K and a cooperativity parameter σ equal to 4 ± 3 × 10^−6^. The latter value is remarkably low, indicating that the observed transition is extremely cooperative; significantly more cooperative than most of the transitions driven by conformational changes reported for covalent and supramolecular polymers to date.^[^ ^69^, ^84^ ^]^

Spectroscopic changes associated with this transition have also been observed in FT‐IR and NMR analyses performed at various temperatures. We notably noticed a significant change in the shape of the C═O band by FT‐IR spectroscopy, from a single band with a maximum of 1638 cm^−1^ above T* to a band with a maximum of 1630 cm^−1^ and an obvious shoulder at ca. 1655 cm^1^ below T* (Figure 2d). Note that the intensity of the N─H band increases concomitantly yet the overall evolution of the shape of this band with the temperature is more gradual (Figure S4). The ^31^P NMR signal associated with PAr_3_ is broad below 303 K as a result of the polydisperse and dynamic nature of the assemblies, yet it can be detected upon sufficient accumulation (Figure S5). This signal significantly broadens between 293 and 283 K, whereas more gradual changes are observed below this temperature. Overall, abrupt spectroscopic changes observed by CD, FT‐IR and NMR analyses all occur at a temperature which is also consistent with the endothermic signal detected by calorimetry upon heating (see below), thus confirming that these changes result from the same phenomenon.

We next determined the salient features associated with this transition. First, virtually identical T* values are obtained upon cooling and heating, and at different heating rates as expected for a reversible transition occurring under thermodynamic control (Figures S4, S6, and S10). Second, CD analyses of solutions with concentrations differing by almost two orders of magnitude show that T* only slightly decreases when the concentration increases (Figures 2c and S6). This indicates that the transition occurs similarly for very long and relatively short assemblies.^6^ Overall, this data is consistent with the intramolecular nature of the transition. This is confirmed by SANS data which shows no significant difference in the radius of the two SP states; single stacks are present in toluene whatever the temperature (Figure 2e), a point that is further confirmed by Small‐Angle X‐ray Scattering (SAXS) data recorded down to 193 K (Figure S7). Note that the q^−1^ dependency of the scattered intensity is maintained down to the lower q values, indicating that the SP chains are longer than 50 nm (lower estimate value for the DP_n_ of 70); yet a potential influence of the transition on the length of the SPs cannot be determined. Third, the influence of water was investigated as recent studies by Meijer and coworkers demonstrated that the binding of water to SP chains can induce structural transitions, such as reversal of their handedness.^[^ ^41^ ^]^ Herein, water molecules are actually found to destabilize the SPs as indicated by an overall decrease of the CD intensity, however CD spectra of both “wet” and “dry” solutions are identical upon normalization indicating that water does not play a role in the transition process (Figure S8). Finally, full CD spectra were recorded below and above T* under specific conditions (Figure S9). The negative couplet for the bisignated CD signal observed at high energy is consistent with the left‐handed arrangement of the hydrogen‐bonding network for both states.^[^ ^83^, ^86^ ^]^ The signal at lower energy exhibits a change in its intensity and shape as mentioned above but remains positive. The transition is thus not associated with a reversal of the handedness of the supramolecular helices.

**Figure 3:**
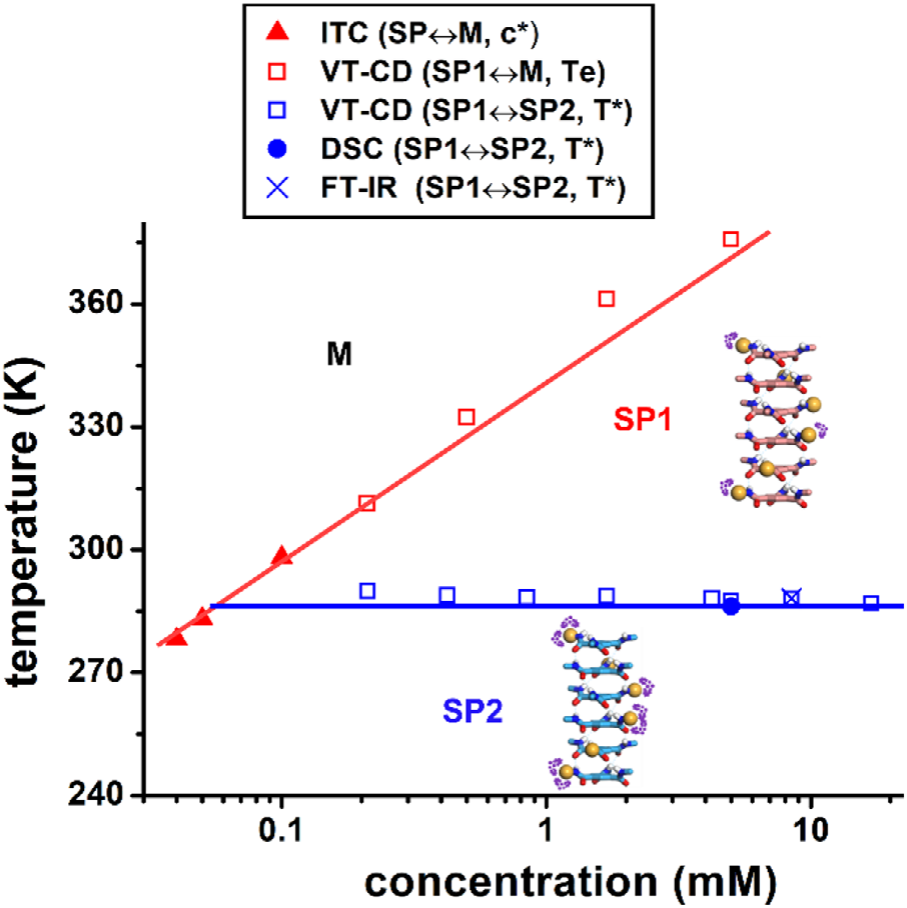
Pseudo‐phase diagram for solutions of BTA P* in toluene. The critical concentrations (c*), elongation temperatures (*T*_e_) and transition temperatures (T*) have been determined according to the analytical techniques indicated in the legend. Lines are guide to the eye for the delimitation of the different domains. M= monomer.

The determination of the critical concentrations by ITC at different temperatures (Figure 1b) and of the elongation and transition temperatures under a large range of concentrations by means of various analytical techniques allows building a pseudo‐phase diagram (Figure 3) with well‐defined domains of predominance for the monomers (M) and the two SP states (SP1 and SP2). The SP2 state is predominant below 288 K for concentrations equal or superior to 10^−4^ M. The consistency of the pseudo‐phase diagram also validates the robustness of the reported data.

The thermodynamic parameters associated with the transition have been determined by high‐sensitivity DSC analyses^[^ ^81^ ^]^ conducted in toluene (Figures 2f and S10). The sharp endothermic peak detected upon heating confirms the highly cooperative nature of the transition. The transition exhibits a favorable enthalpy, yet its value is low (2.38 ± 0.04 kJ.mol^−1^). Transition enthalpies of 3 and 5 kJ.mol^−1^ have been reported for transitions involving differential solvation for the SP states^[^ ^50^ ^]^ and change in the thickness of the SP chains,^[^ ^69^ ^]^ respectively. These transitions were accompanied by a drastic conformational change of the SPs; a reversal of the SP handedness or a reorganization of the hydrogen‐bonding network. The above data suggest that the present transition does not involve such an important conformational change of the SP, but rather a different organization of the lateral chains notably the lateral diphenylphosphino group. Indeed, the observation of better‐resolved CD and FT‐IR bands suggest a more regular organization of these groups for the monomers present in the SP2 state. In this regard, broadening of the ^31^P NMR signal could be attributed to a lower dynamicity of the BTA P* monomers, which does not allow to average the different conformational states of BTA monomers present in the SP2 state. A clearer picture of the SP states will be proposed in the last part of the manuscript. This subtle conformational change is possibly driven by solvation, a point that will be investigated in the next paragraph.

### Transition Between the Two SP States in Other Solvents

**Figure 4:**
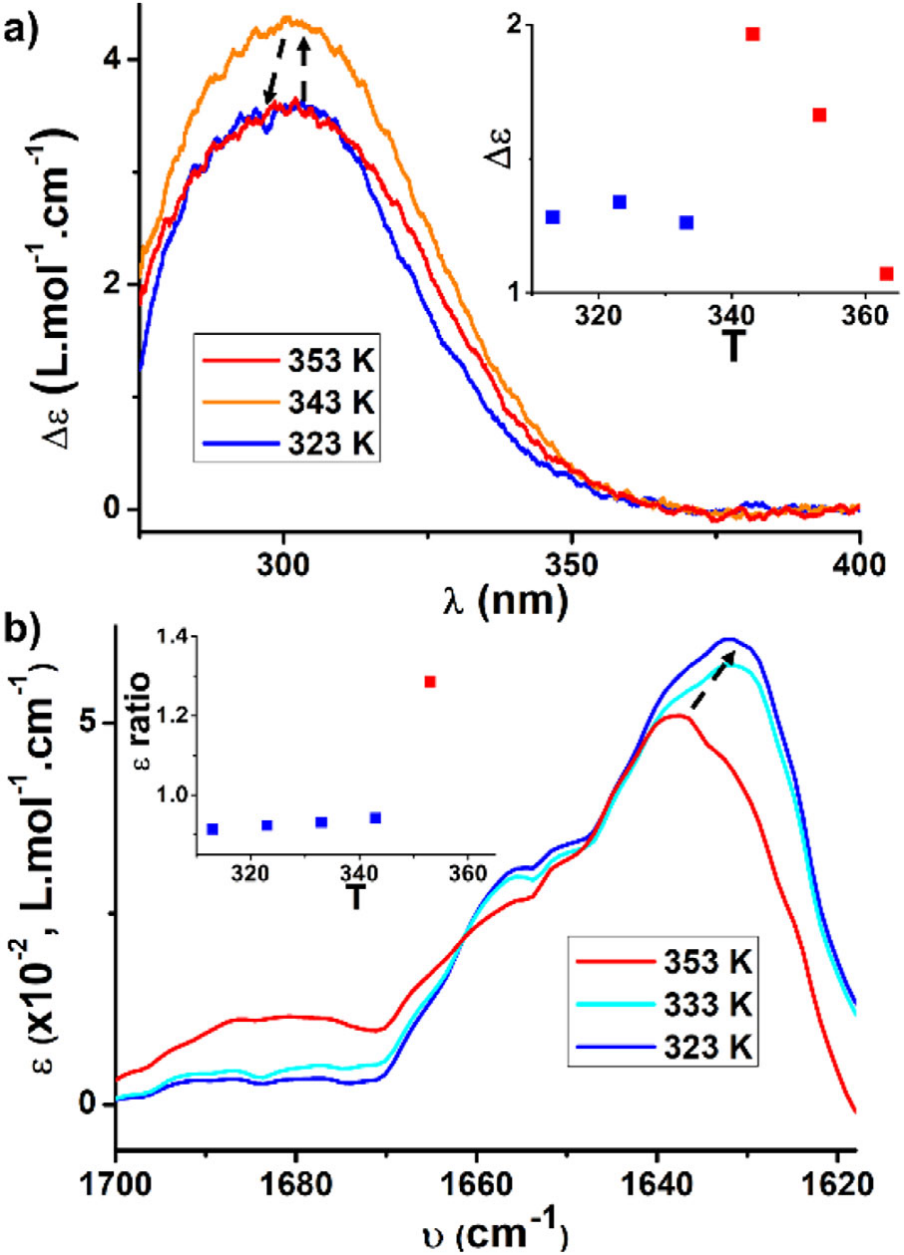
a) CD spectra of a 5.0 mM solution of BTA P* in benzene recorded at different temperatures (only 353 K, 343 K and 323 K are shown here, see Figure S11a for all spectra, recorded upon cooling, 1 K.min^−1^). Arrows help to visualize the evolution of the CD bands when the temperature decreases. Insert: plot of the molar CD values at 330 nm as a function of the temperature. b) FT‐IR spectra of a 5.0 mM solution of BTA P* in benzene recorded at different temperatures (only 353 K, 333 K, and 323 K are shown here, see Figure S11b for all spectra, recorded upon heating, 1 K.min^−1^). Zoom on the amide I C═O band. The arrow helps to visualize the evolution of the FT‐IR bands when the temperature decreases. Insert: plot of the ratio of ε values at 1638 and 1630 cm^−1^ as a function of the temperature extracted from FT‐IR data.

Upon cooling of a 5.0 mM solution of BTA P* in benzene, spectroscopic changes (Figure S11ab) similar to those observed in toluene have been detected: comparison of the normalized CD and FT‐IR spectra unambiguously ascertains that the same SP states are present in both solvents (Figure S11cd). However, the transition occurs at a remarkably higher temperature in benzene as can be deduced from the shifts of the CD signal (between 333 K and 343 K, Figure 4a) and of the FT‐IR amide I band (between 343 K and 353 K, Figure 4b). Precise monitoring of the transition by recording the CD intensity at 330 nm (Figure S11e) indeed indicates a difference in the transition temperature of 52 K between benzene and toluene, which is also consistent with the shift seen by FT‐IR (Figure S11f). This difference is quite remarkable regarding the similar physicochemical properties of these two solvents.

**Figure 5:**
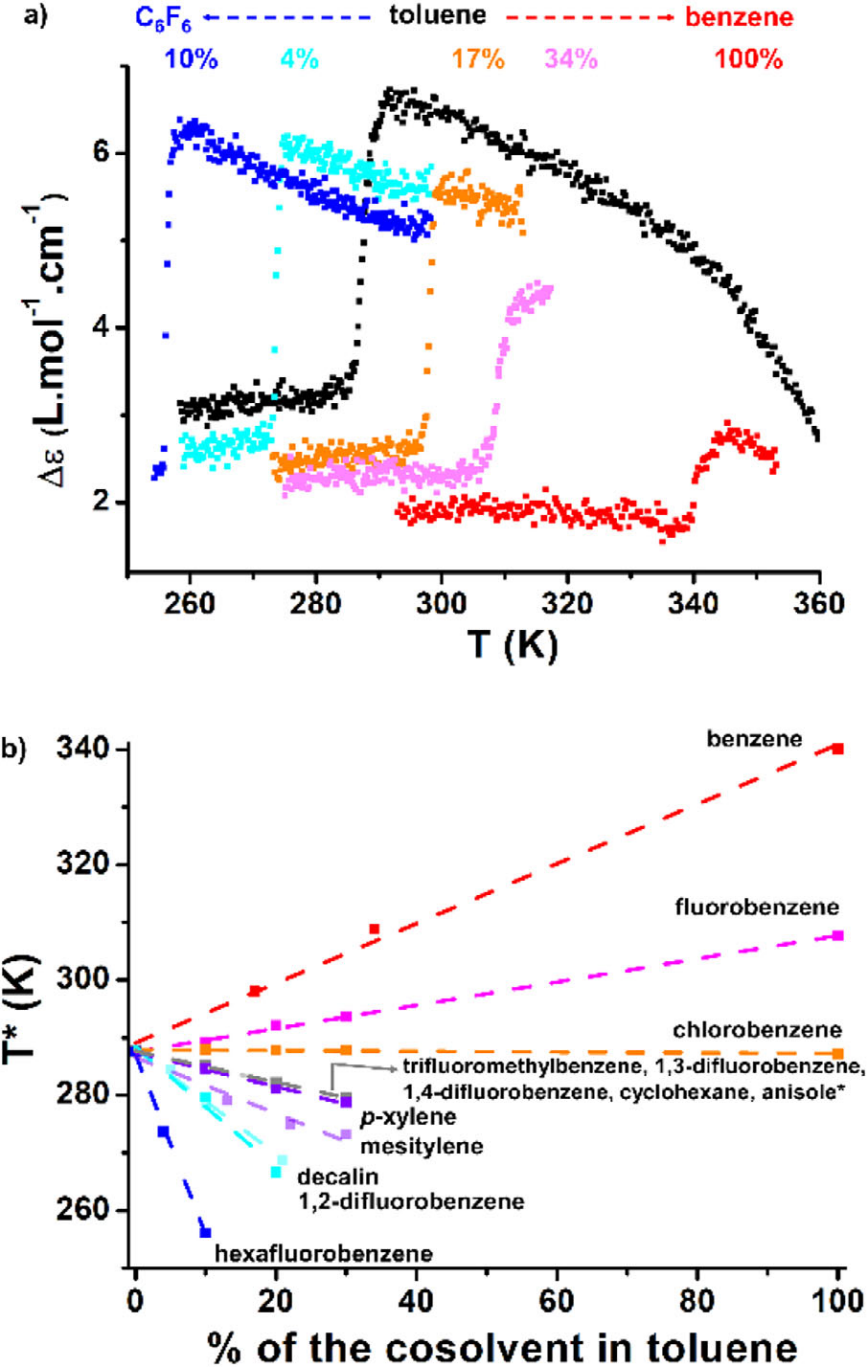
a) CD intensity (*λ* = 330 nm) as a function of the temperature for 5.0 mM solutions of BTA P* in toluene/benzene and toluene/hexafluorobenzene mixtures. The indicated percentages correspond to the proportion of cosolvent in volume (vol%). Data recorded upon cooling (0.5 K.min^−1^). b) Plot of the T* for the 13 combinations between toluene and a cosolvent as a function of the vol% of the cosolvent.

This result prompts us to investigate the effect of solvents of similar polarities on the transition between the two SP states. We first compared the influence of benzene (*E_T_* 30 = 34.3) and hexafluorobenzene (*E_T_* 30 = 34.2), two solvents with identical *E_T_* 30 values. Monitoring by CD of the transition for mixtures between toluene and one of these two solvents reveals drastically opposite behaviors: the presence of a few percent of hexafluorobenzene (vol%) shifts the transition to lower temperatures whilst benzene increases the transition temperature as mentioned above (Figure 5a). This different trend is well reflected by plotting T* as a function of the percentage of cosolvent in toluene (Figure 5b). The evolution is linear, which means that the transition is affected proportionally to the bulk composition of the solvent mixture, thus discarding any effects related to solvent partitioning or preferential solvation at the (macro)molecular level.

To get a more global view of the solvent effect, the transition was probed in 13 mixtures composed of toluene and various cosolvents. The amount of cosolvent has been adapted to make the transition observable in the range of temperature accessible by CD spectroscopy (255 K–383 K) and in the upper limit of the cosolvent boiling point. Only five pure solvents (benzene, fluorobenzene, chlorobenzene, anisole, decalin) in addition to toluene have been investigated because of solubility limitations (Table S1) or because a trend was already clear with a few solvent mixtures, notably for those leading to a decrease in T*. Hence, the transition could not be detected in pure anisole and decalin because it occurs below 255 K. Remarkably, all mixtures exhibit a sharp change in their CD intensity, inferring a similar transition between the two SP states (Figures S12–S14). In most cases, similar molar CD values are obtained below the transition temperature, which suggests that while the solvent impacts the transition temperature, it does not significantly impact the structure of SP2. Variation in the molar CD values above the transition temperature is tentatively attributed to the difference in elongation temperatures between solvents of different natures (as probed in Figure 1d), which will affect the CD intensity of SP1. However, there is no obvious relation between the molar CD value of SP1 and the transition temperature, thus discarding a potential effect of its (de)stabilization on the transition under scrutiny.

The result for the 13 combinations, plotted in Figure 5b, can be summarized as follows: i) two solvents (benzene, fluorobenzene) lead to an increase in T*, ii) chlorobenzene behaves similarly as toluene, iii) seven solvents (five plotted in grey squares, as well as *p*‐xylene and mesitylene) decrease smoothly the T*, iv) three solvents (decalin, 1,2‐difluorotoluene, hexafluorobenzene) more drastically decrease T*; hexafluorobenzene being the most impactful one. The transition temperature can be tuned in a predictable way from 255 K to 340 K, i.e., over 85 K range, by selecting the ratio and nature of the cosolvent. Solvation obviously plays a key role in the subtle conformational change leading to the cooperative transition; a more detailed investigation of the relationship between T* and solvent parameters will be given in the last part of the manuscript.

### Macroscopic Properties of the Two SP States in Different Solvents

**Figure 6:**
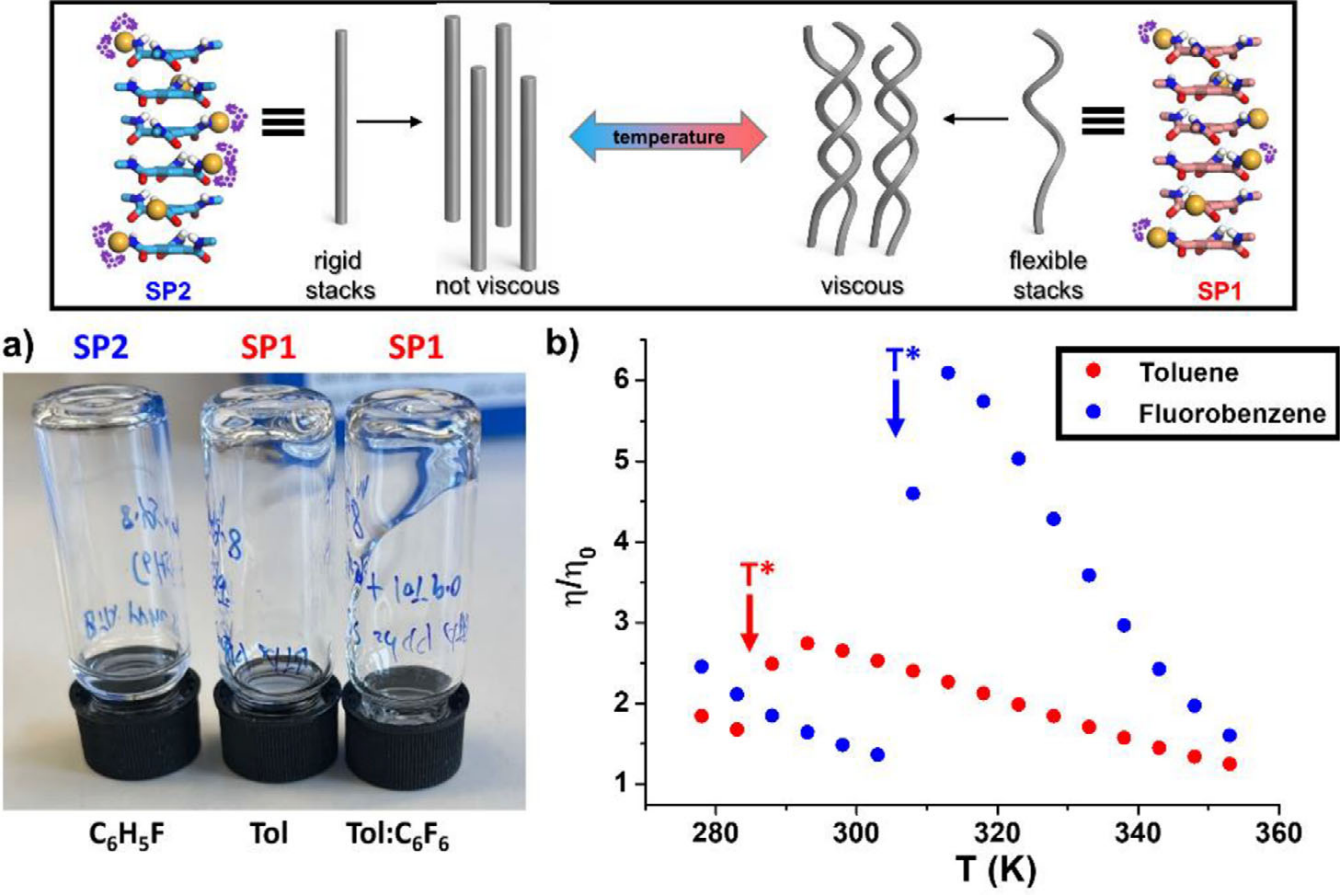
a) Pictures of 8.5 mM solutions of BTA P* in fluorobenzene, in toluene and in a 90:10 mixture of toluene/hexafluorobenzene (vol%) at 293 K. The corresponding SP states are indicated above the vials. b) Plot of the relative viscosities as a function of the temperature for solutions of BTA P* in toluene (2.0 mM) and fluorobenzene (6.0 mM), together with an indication of the T* for the corresponding solutions as determined by high‐sensitivity DSC. The concentration has been adapted for practical reasons – the viscosities are higher at 6.0 mM allowing to better probe the thermothickening effect, however the viscosity of BTA P* in toluene at 293 K is too high at this concentration for the solution to be handled properly. Top: proposed rationale for the thermothickening effect.

We wondered whether the access to two distinct states could be useful to tune the properties displayed by the SPs of BTA P*. It was anticipated that the (local) conformational change revealed by spectroscopy, even though not yielding a larger change at the mesoscale such as an inversion of the handedness or the bundling of single helices, could anyway lead to differences in the shape of the macromolecule and the interaction between macromolecules. A first hint towards this possibility is suggested by the fact that, at 293 K, the solutions of BTA P* in toluene and in the toluene/hexafluorobenzene mixture (90:10, vol%) are viscous whilst that in fluorobenzene is fluid (Figure 6a). It is established from the above data that the former two are in the SP1 state whilst the latter is in the SP2 state at this temperature. To firmly establish a relationship between the SP states and the rheological properties, the viscosity of solutions of BTA P* has been monitored at various temperatures in toluene and fluorobenzene (Figure 6b). At the lowest investigated temperature (278 K) the viscosity of the solutions is low in both solvents, i.e., only slightly more viscous than the solvent itself. Upon heating, the viscosity of the solutions decreases and then experiences a sharp increase at a temperature close to the transition temperature, i.e., 285 K and 305 K for toluene and fluorobenzene, respectively. After reaching a maximum at a few degrees above T*, the viscosity then gradually decreases as expected, because of the dissociation of hydrogen‐bonded SPs. This temperature‐dependent rheological behavior of solutions of BTA P* is quite unexpected since most of SPs reported in the literature exhibit a monotonic evolution of the viscosity in apolar solvents, namely a decrease of the viscosity as the temperature rises.^[^ ^69^ ^]^ It is conventionally explained by the fact that entanglements between polymer chains are disrupted upon heating. Thermothickening, i.e., an increase of the viscosity upon heating, is a rare phenomenon for SPs in apolar solvents.^[^ ^87^, ^88^ ^]^ In the present case, this effect is related to the temperature‐triggered conformational and solvation change which occurs for the SPs formed by BTA P*: solutions containing predominantly SP1 are more viscous than those containing SP2 (at a temperature close to the transition temperature). Upon looking for a rationale for this unusual effect, it is very unlikely that it comes from an increase in the length of the polymer chains above T*. One can more convincingly explain this thermothickening phenomenon by a difference in the flexibility of the single helices present in both states; those formed by SP2 (below T*) would be rigid, thus preventing/limiting entanglements, whilst those of SP1 (above T*) would be more flexible generating more entanglements between the chains. These entanglements are likely to be rarely and randomly distributed along the polymer chain, acting as bridges between long unaltered single chains since they have not been detected in the SANS data (Figure 2e). A schematic representation of this proposition is shown in Figure 6.

**Figure 7:**
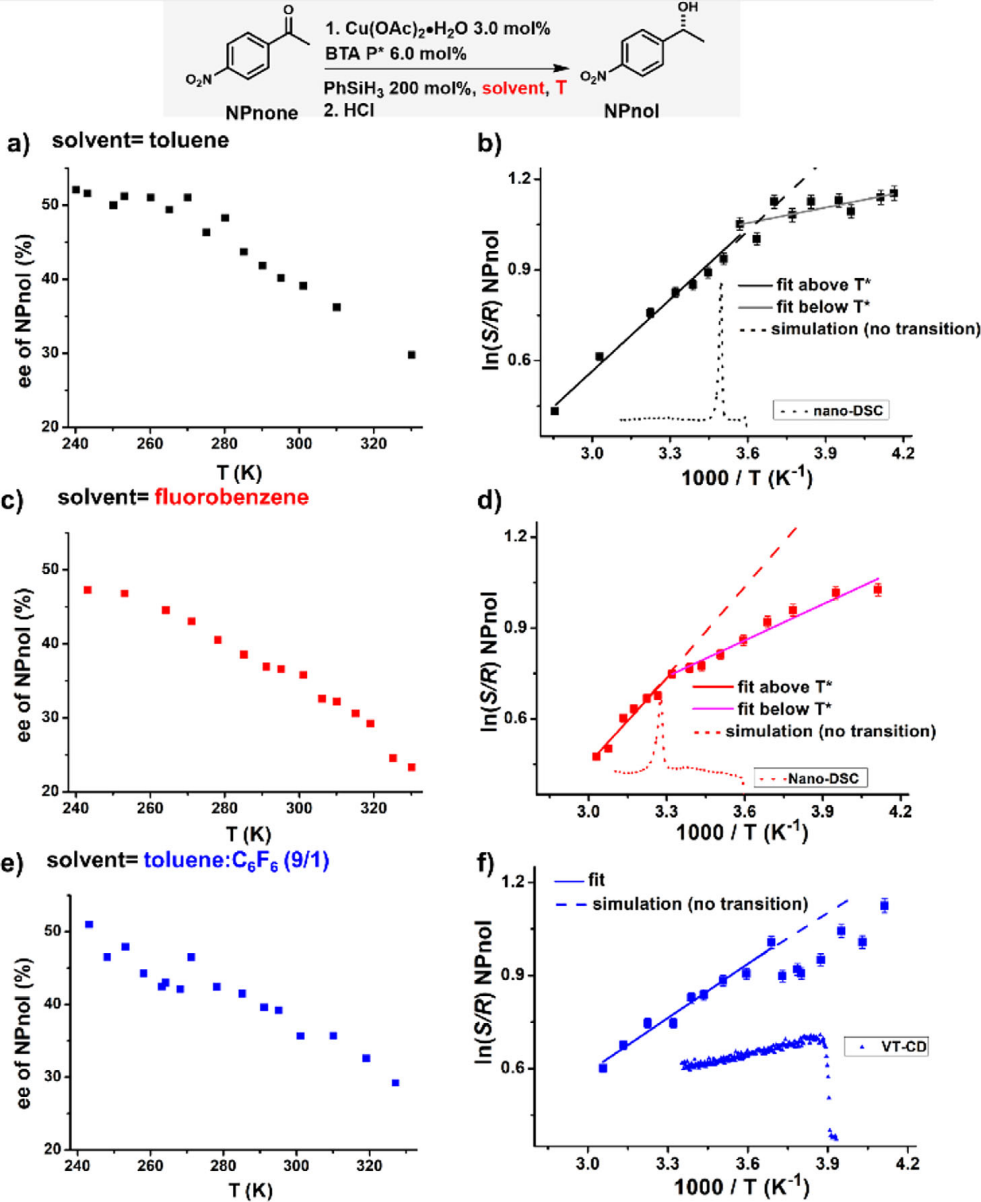
Plot of enantioselectivities in **NPnol** as a function of the temperature for the copper‐catalyzed hydrosilylation reaction with solutions of BTA P* in toluene a), in fluorobenzene c) and in a 90:10 mixture of toluene and hexafluorobenzene (vol%, e). Corresponding Eyring plots b), d), f) with regression lines and analytical traces – obtained by DSC or VT‐CD‐ to visualize the position of the transition temperature. Simulation of the Eyring plot with a single regression line is represented by a dotted line for each solvent to emphasize the effect of T* on the catalytic data. The data for three solvents is compared in Figure S15.

We next study the influence of the transition on the enantioselectivity displayed by SPs of BTA P* functionalized with copper atoms. These copper sites promote the hydrosilylation of 4‐nitroacetophenone, the substrate of reference employed in our previous studies.^[^ ^73^, ^74^, ^75^, ^79^, ^89^, ^90^ ^]^ These studies indicated that the enantioselectivity displayed by BTA SPs exclusively stems from their helical chirality; this is thus another property which is controlled at the (supra)macromolecular scale. The enantioselectivity in the product of the reaction (**NPnol**) has been measured between 248 K and 327 K in toluene (Figure 7a), in fluorobenzene (Figure 7c) and in 90:10 toluene/hexafluorobenzene mixture (vol%, Figure 7e). The high concentration in BTA P* (16.9 mM) used in these experiments ensures that assemblies are maintained up to 400 K (Figure 3). Regarding the data in toluene, the enantioselectivity first increases upon lowering the temperature, then plateaus below 280 K at a value of ca. 50% enantiomeric excess (ee, Figure 7a). Two distinct straight lines can be deduced from the corresponding Eyring plot (Figure 7b) with a kink at ca. 280 K, i.e., a temperature close to T* in this solvent (286–288 K). In fluorobenzene, no obvious plateauing of the selectivity is observed (Figure 7c) but the Eyring plot anyway reveals two distinct linear trends; the selectivity increases upon lowering of the temperature but in a non‐monotonic way (Figure 7d). The change in slope occurs at a temperature (301 K) again close to T* (305 K). The data obtained in the toluene/hexafluorobenzene mixture (Figure 7e) do not yield two obvious straight lines in the Eyring plot (Figure 7f), but a change in the evolution of the selectivity is anyway detected at 268 K, close to the T* value recorded for this solvent mixture (256 K).

The evolution of the enantioselectivity displayed by BTA P* coordinated to copper as a function of the temperature drastically differs depending on the nature of the solvent. The reported data ascertains that the sudden change in the slopes of the Eyring plots is related to the structural transition which occurs within single helices of BTA P*. It is likely due to different conformation and solvation of the copper centers in the two SP states whilst the influence of the aforementioned entanglements on the selectively outcome cannot be excluded (for a more detailed discussion, see Figure S15). To the best of our knowledge, the correlation between a subtle change in the conformation/solvation of the catalytic sites and a difference in the energetics governing the selectivity as reported here is different from the reported examples for which this effect was attributed to catalyst aggregation, a change in the nature of the enantiodetermining step, a modification in the nature of substrate‐solvent clusters, or more intricated phenomena.^[^ ^91^, ^92^ ^]^

### Dissecting the Nature of the Structural Transition

The above data allow us to determine that solvation plays a key role in the subtle conformational change that leads to observation of the two SP states for BTA P*. Additional efforts have been dedicated to better apprehend the origin and salient features of this conformational transition and how it is affected by solvation.

#### Probing the Influence of the Molecular Structure

**Figure 8:**
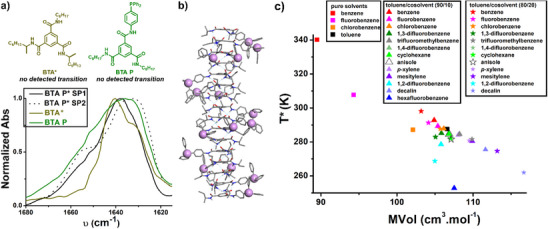
a) Molecular structures of the reference compounds BTA* and **BTA P**, and normalized FT‐IR spectra in the amide I region. 8.5 mM solutions of either BTA P* (293 K for SP1, 283 K for SP2), BTA* (293 K) or **BTA P** (293 K) have been analyzed in toluene (and toluene‐d_8_) at different temperatures, see the full spectra in Figure S16. b) Computed structure of a hexadecamer of an analogue of BTA P* with (*S*)‐*sec*‐butyl side chains; the phosphorus atoms have been emphasized as pink spheres to better visualize the right‐handed orientation of the peripheral diphenylphosphino groups, which is of opposite direction relatively to hydrogen‐bonding network. c) Plot of the T* values obtained for BTA P* solutions in pure solvents and in toluene/cosolvent mixtures as a function of the molar volume of the solvents. The T* values of the mixtures correspond to those of mixtures with identical mol% of cosolvents (10 mol% or 20 mol%) to be consistent with the fact that the molar volumes of the mixtures are obtained by the average weight of the molar volumes of the pure solvents (Table S2).

To delineate the structural factors that conduct the observation of two SP states, two monomers with precise chemical mutations relative to BTA P* have been studied (Figure 8a): the side chains of BTA P* have been independently replaced by octyl groups to yield **BTA P** (an achiral version of BTA P*) or BTA* (a version of BTA P* lacking the diphenylphosphino appended side chain). Variable‐temperature FT‐IR analyses of these three monomers in toluene show that they all assemble into hydrogen‐bonded stacks but only BTA P* displays a transition between two SP states within the investigated temperature range (268–353 K, Figure S16ad). In agreement with the absence of transition, no thermothickening effect has been detected for BTA* and **BTA P** (Figure S16e), further establishing that the aforementioned macroscopic properties are related to the existence of the two SP states. This comparative study between monomers of similar structures reveals that both the presence of the lateral PAr_3_ group and of stereogenic center(s) are required to get two distinct SP states in an observable temperature range.

#### Probing the Structure of the Fibers in Solid‐State

We wondered whether additional structural information could be gained by analyses conducted in solid‐state. We have probed the morphology of films obtained by spin‐coating toluene or fluorobenzene solutions of BTA P* onto silica wafer by Atomic Force Microscopy (AFM). Spin‐coating has been performed at 293 K, a temperature for which SP1 and SP2 are the dominant states in toluene and fluorobenzene, respectively. Both samples exhibit large fibers which are actually bundles of single helical SPs, bundling being likely promoted by the evaporation process. This is reminiscent of the morphologies found for other hydrogen‐bonded SPs in the literature (Figure S17).^[^ ^41^, ^50^ ^]^ Those obtained in fluorobenzene appear to be better defined and to more densely occupy the surface of the films; an observation compatible with the spectroscopic and rheological data which infers that single helices present in the SP2 state are better organized (i.e., contains fewer defects). However, the impact of solvent evaporation on the morphologies could not be evaluated, which let open the question on the actual origin of the different morphologies observed in these thin films.

#### Probing the Relative Arrangement of the BTA P* Monomers in the Single Helices

Whilst the core of the assembly is well constrained by the non‐covalent interactions, the relative position between the lateral diphenylphosphino and (1*S*)‐methylheptyl groups may lead to a high number of possible structures for the single helices (not counting for other possible subtle conformational differences, e.g. in the dihedral angle between the amide functions and the central BTA ring and the relative orientation of the amide functions).^[^ ^65^ ^]^ The structural difference between the two SP states is likely driven by differential solvation at various temperatures (see below), but a sub(molecular) picture of this subtle difference is precluded by the many possible conformations. Hence, we directed our effort to determine the possible relative arrangement of the BTA P* monomers in the assemblies. CD analyses of BTA P* in toluene show the presence of two bisignated signals of similar intensities (Figure S9): a negative couplet and a positive couplet at high and low energy, respectively. Whilst the former ascertains the presence of left‐handed helices for the hydrogen‐bonding network (internal helical array),^[^ ^83^, ^86^ ^]^ the latter suggests an opposite helical organization for the lateral diphenylphosphino moieties (external helical array).^[^ ^93^ ^]^ The high CD intensity of the positive couplet is also consistent with a well‐defined organization of these diphenylphosphino groups. We thus surmise that such an organization of the peripheral side chains with internal and external helices of opposite handednesses is plausible. Please note that this structure is valid for both the SP1 and SP2 states since the CD couplets exhibit the same signs in both states, whilst shifts of these bands are too small to be interpreted because they are induced by a subtle change in the conformation and solvation sphere of the lateral PPh_2_ groups. FT‐IR analyses (see discussion in Figure S16) are consistent with a more regular organization of the amide functions below the transition temperature, maybe through exclusive binding between the aromatic and alkyl amide functions in the SP2 state. A hexadecamer of an analogue of BTA P* with (*S*)‐*sec*‐butyl side chains has been modelled following these structural requirements (Figure 8b). The good agreement between experimental and computed CD spectra (Figure S18) argues in favor of such an organization of the lateral chains in the SP assemblies formed by BTA P*.

Our hypothesis, which agrees with the experimental observations, is that a conformational change in a structure of the type of the one presented in Figure 8b occurs upon differential solvation of the lateral diphenylphosphino groups; the low temperature SP state being more solvated.^[^ ^50^ ^]^ It is expected that the change in the solvation volume is modest at the scale of the monomer but cooperatively transfers to other groups in the polymer leading a detectable conformational change at the mesoscale. The overall organization of the monomers into the SPs is not dramatically altered, most probably a more regular organization of the hydrogen‐bonding network occurs that leads to a more regular arrangement of the lateral diphenylphosphino groups at the periphery of the single helices. A representation of the two SP states, consistent with our experimental and computational data, is shown in Scheme 1.

#### Probing the Solvation Features

The transition temperature values obtained for solutions of BTA P* in 4 pure solvents and 13 toluene/cosolvent mixtures can be compared with solvent parameters. The T* values are not correlated to standard solvent polarity descriptors (Figure S19) such as *E_T_* 30 values and individual terms of the Hansen Solubility Parameters (HSP). Likewise, solvents which yield low T* values do not occupy a specific position in the 3D Hansen space. Unlike the monomer‐SP1 assembly process, the conformational change between SP1 and SP2 states is not responsive to solvent polarity, a point that was already obvious from the plot of the T* in the different solvents presented in Figure 5b. However, a reasonable relationship is identified between T* and the solvent molecular volume (Figure 8c); the general trend being that the larger the solvent, the lower the transition temperature. A similar trend is obtained when molar, accessible (Figure S20a) or van der Waals (Figure S20b) volumes are considered. It can be surmised that all the tested solvents have similar affinity, on electronic grounds, for the lateral PAr_3_ group, but smaller ones lead to better solvation at low temperature. This hypothesis seems valid not only for aromatic solvents but also for alkanes, since cyclohexane and decalin mixtures fit well into the overall trend, their size (rather than lower affinity) seems to be the reason for the decrease in T* observed in their respective solvent mixtures (see Figure S20 for a more detailed discussion on the correlation between T* and solvent volume).

It is quite remarkable that the plot in Figure 8c with a single solvent parameter provides a fair picture of the solvation process for which both enthalpic and entropic contributions can be at work in triggering the conformational transition observed between the SP states of BTA P*.

To better quantify the energetics underlying the solvation process, we used a model that was previously implemented to compare the excess free energy of binding of two (bisurea) monomers into 1D nanostructures in a given solvent.^[^ ^71^, ^94^ ^]^ The same model can be used to compare the excess free energy of binding of a given monomer into 1D nanostructures in two different solvents. It was established that this excess free energy is directly linked to the transition temperatures observed between the nanostructures in each solvent according to the formula indicated in the caption of Table 1. In the present case, we selected the data recorded in toluene and fluorobenzene, the latter leading to an increase in the transition temperature by 19 K. The transition enthalpies measured by high‐sensitivity DSC (Figure S10) are similar, the value in fluorobenzene is actually lower than the one in toluene. The thermodynamic parameters extracted from these data, i.e., the differential excess free energy, enthalpy and entropy between the SP1 and SP2 states in fluorobenzene relatively to toluene, are compiled in Table 1. The enhanced stability of the SP2 state in fluorobenzene relative to toluene (ΔΔ*G* = −0.13 kJ.mol^−1^) benefits from a favorable entropic contribution (−TΔΔS = −0.59 kJ.mol^−1^) and an unfavorable enthalpic contribution (ΔΔ*H* = 0.46 kJ.mol^−1^). This unambiguously demonstrates that the shift of the transition temperature to higher value, i.e., the enhanced thermodynamic stability of the SP2 state, is driven by entropy, not by enthalpy. This analysis, in conjunction with the aforementioned data which ascertains the role of the solvation in the conformational transition experienced by BTA P* monomers in their single helices, reinforces the idea that solvation entropy is the main lever which allows us to tune the T* values measured in a range of solvents of similar polarities.

**Table 1:** Values for the parameters deduced from the T* and transition enthalpies obtained for BTA P* solutions in toluene and fluorobenzene.

Solvent	T*^a)^ (±0.2 K)	Δ*H* ^a)^ (±0.04 kJ.mol^−1^)	ΔΔ*H* ^b)^ (kJ.mol^−1^)	ΔΔ*G* ^c)^ (kJ.mol^−1^)	TΔΔS^d)^ (kJ.mol^−1^)
Toluene	286.2	−2.38	0.46	−0.13	0.59
Fluoro‐benzene	305.3	−1.92			

^a)^ Transition temperature and enthalpy for BTA P* solutions determined by high‐sensitivity DSC experiments.

^b)^ Difference in transition enthalpies for solutions of BTA P* (= ΔH_fluorobenzene_ − ΔH_toluene_), uncertainty ± 0.04 kJ.mol^−1^.

^c)^ Excess free energy computed through ΔΔG = ΔH_fluorobenzene_×(T*_fluorobenzene_ − T*_toluene_)/T*_toluene_, uncertainty ±0.004 kJ.mol^−1^.

^d)^ Uncertainty ±0.04 kJ/mol^−1^.

## Conclusions

The thorough investigation of the assembly properties of BTA P*, a structurally‐simple *C*_2_‐symmetric benzene‐1,3,5‐tricarboxamide monomer, in solvents of similar polarities by multifarious analytical techniques at various scales reveal several important features. First, it allows us to identify a highly cooperative structural transition between two supramolecular polymer (SP) states which can consistently be varied over a temperature range of 85 K by playing on the nature and amount of the toluene cosolvent. Second, our data ascertains that differential solvation of the two SP states triggers a conformational change which is detected spectroscopically but is not related to a drastic change at the mesoscale such as helix inversion or bundling of the single helices, like precedent examples in the literature.^[^ ^50^, ^62^ ^]^ A higher solvation of the lateral PAr_3_ occurs at low temperatures triggers a more regular organization of the hydrogen bonding network and leads to a better organization of the lateral chains. A modelled hexadecamer structure is proposed for which the PAr_3_ groups adopt an ideal helical arrangement at the periphery of the assemblies which is of opposite handedness relatively to the inner hydrogen‐bonding network involving the amide groups. Third, the volume of the solvent molecules appears to be the most suitable parameter to tune the transition temperatures between the two SP states in a predictable manner. Lastly, the different conformations in the SP states, even though subtle, lead to remarkable differences in their macroscopic properties; a thermothickening effect can notably be shifted to a higher temperature which is interesting in view of various applications such as viscosity index improvers.^[^ ^88^ ^]^ Minimizing the entropic cost of solvation thus appears to be an interesting lever to control the macroscopic properties of supramolecular polymers, an approach which could prove to be advantageous regarding the existing strategies based on playing on the molecular structure of the monomer,^[^ ^75^, ^79^ ^]^ on the presence of additives or on tuning the aggregation pathway.^[^ ^95^ ^]^

## Conflict of Interests

The authors declare no conflict of interest.

## Supporting Information

The authors have cited additional references within the Supporting Information.^[^ ^3^, ^96^, ^97^, ^98^, ^99^, ^100^, ^101^, ^102^, ^103^, ^104^, ^105^ ^]^

## Supporting information



Supporting Information

## Data Availability

The data that support the findings of this study are available in the supporting information of this article.
