# Supplementary material for: Solvation Entropy as a Lever for Steering the Macroscopic Properties of a Functional Supramolecular Helical Polymer
Source: Angew Chem Int Ed Engl. 2025 Nov 20;65(3):e21365. doi: 10.1002/anie.202521365 (PMC12811656; doi:10.1002/anie.202521365)
Supplement: Supplementary file 1 — Supporting Information [file ANIE-65-e21365-s001.pdf]

Supporting Information

Solvation Entropy as a Lever for Steering the Macroscopic Properties of a Functional Supramolecular Helical Polymer

Huanjun Kong,[1] Mayte A. Martínez-Aguirre,[1] Yan Li,[1] Tomoyuki Ikai,[2] Eiji Yashima,[2] Gangamallaiah Velpula,[3] Steven De Feyter,[3] Pierre-Antoine Albouy,4 Komivi Akpo,[5] Patrick Brocorens,[5] Roberto Lazzaroni,[5] Laurent Bouteiller,[1] and Matthieu Raynal[1,*]

1 Sorbonne Université, CNRS, Institut Parisien de Chimie Moléculaire, Equipe Chimie des Polymères, 4 Place Jussieu, 75005 Paris (France) e-mail: matthieu.raynal@sorbonne-universite.fr.
2 Department of Molecular and Macromolecular Chemistry, Graduate, School of Engineering, Nagoya University, Chikusa-ku, Nagoya 464-8603 (Japan)
3 Division of Molecular Imaging and Photonics, Department of Chemistry, KU Leuven, Celestijnenlaan 200F, 3001, B 3001 Leuven (Belgium)
4 Laboratoire de Physique des Solides, CNRS, Université Paris-Sud, Université Paris-Saclay, 91400 Orsay, France
5 Service de Chimie des Matériaux Nouveaux, Institut de Recherche sur les Matériaux, Université de Mons, Place du Parc, 20, B-7000, Mons (Belgium)

Supplementary Figures and Tables (Figures S1-S20, Tables S1-S3) ________________ 2
Materials and methods (Figure S21) ________________ 24
Catalytic experiments (Tables S4-S6) ________________ 29
Synthesis of **BTA P*** (Figures S22-S25) ________________ 32
Optical purity of **BTA P*** (Figure S26) ________________ 35
Stability of **BTA P*** (Figures S27-28) ________________ 36
Selected chiral GC analyses (Figures S29-S39) ________________ 37
References ________________ 43

Supplementary Figures and Tables (Figures S1-S20, Tables S1-S3)

solvent	E_T30[a]	solubility	solvent	E_T30[a]	solubility
1,2-difluorobenzene*	38	S	*p*-xylene*	33.1	S
1,3-difluorobenzene*	37.3	S	mesitylene*	32.9	S
anisole*	37.1	S	1-chlorooctane	*nd*	I
(trifluoromethyl)benzene*	*nd*	P	cyclohexane*	*nd*	I
fluorobenzene*	37	S	butylcyclohexane	*nd*	I
chlorobenzene*	36.8	S	decalin*	31.2	S
1,4-difluorobenzene*	36.4	S	*n*-octane	31	I
benzene*	34.3	S	*iso*-octane	*nd*	I
hexafluorobenzene*	34.2	I	*n*-decane	31	I
toluene	33.9	S	2,2,4,4,6,8,8-heptamethylnonane	*nd*	I
tetralin	*nd*	I	*n*-dodecane	31	I

Table S1. Solubility of **BTA P*** (0.5 mM) in different solvents at 293 K. S: soluble. P: precipitate (compound is soluble in the hot solution but precipitates at 293 K). I: insoluble (compound is not soluble in the hot solution). Solvents indicated with * will be used as cosolvents of toluene in this study.

[a]: Data from references[1,2]. *nd*: not determined.

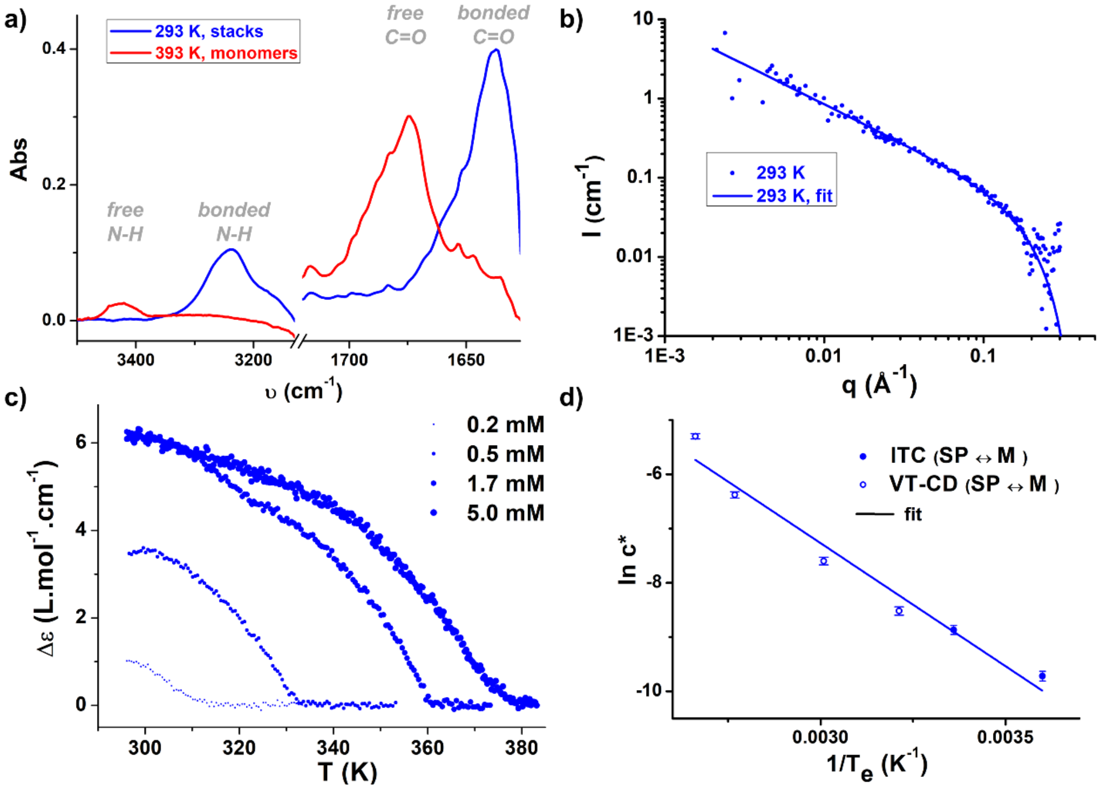

Figure S1. a) FT-IR analyses of a 2.0 mM solution of **BTA P*** in toluene at 293 K and 393 K. b) SANS analysis of an 8.7 mM (6 g.L^{-1}) solution of **BTA P*** in toluene-d_8 at 293 K with the corresponding fit for rigid rods. The extracted radius and number of molecules in the cross-section (see Figure S7) indicate that **BTA P*** mostly exists under the form of long single stacks. c) CD intensity (λ= 330 nm, molar CD) as a function of the temperature (above 296 K) for solutions of **BTA P*** at different concentrations in toluene. Data recorded upon cooling (0.5 K.min^{-1}). d) Van't Hoff plot: natural logarithm of the critical concentration versus the reciprocal T_e as determined by VT-CD and ITC analyses. Linear regression of the data points allows to extract the assembly enthalpy (ΔH) from the slope (-ΔH/R with R being the molar gas constant, R^2= 0.96). The extracted assembly enthalpy (*ca.* 9 kcal.mol^{-1}) is of the same order as the enthalpy release upon elongation measured by ITC (≈ 6 kcal.mol^{-1}, Figure 1b) and that deduced from fitting the CD data (8 kcal.mol^{-1}, Figure 1c).

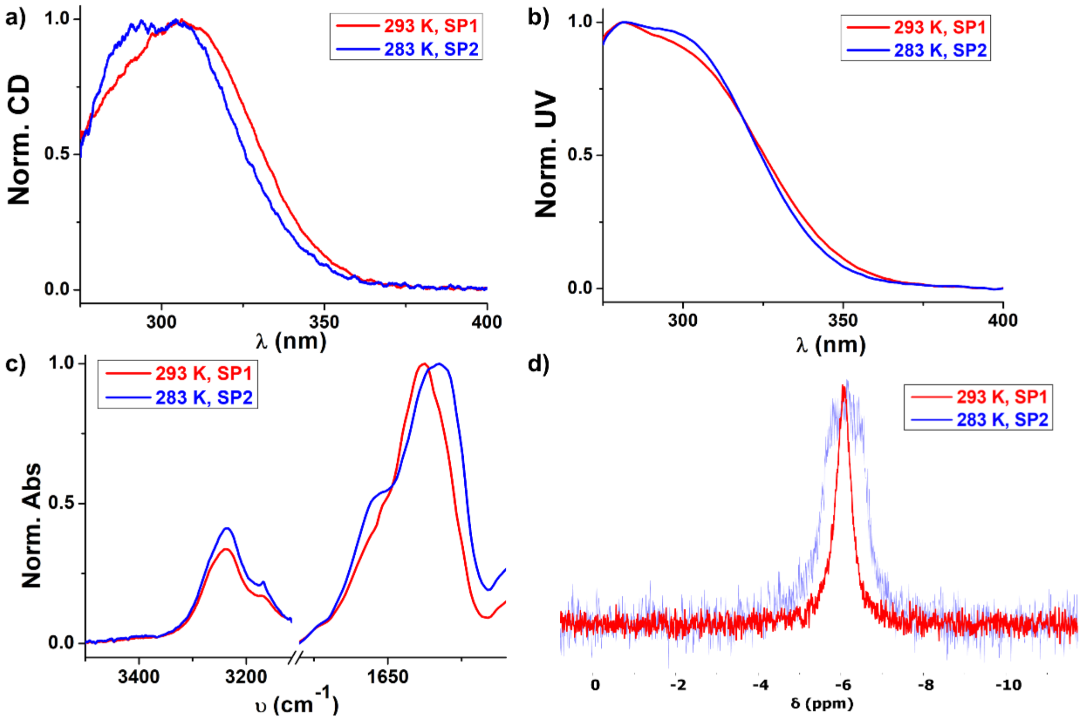

Figure S2. Characterization of the two SP states of BTA P* in toluene. a) Normalized CD data at 293 K (SP1) and 283 K (SP2). b) Normalized UV-Vis data at 293 K (SP1) and 283 K (SP2). c) Normalized FT-IR data at 293 K (SP1) and 283 K (SP2). Zoom on the N─H and C=O regions. d) Normalized $^{31}P\{^{1}H\}$ data at 293 K (SP1) and 283 K (SP2). All spectra have been normalized to 1 at their maximum.

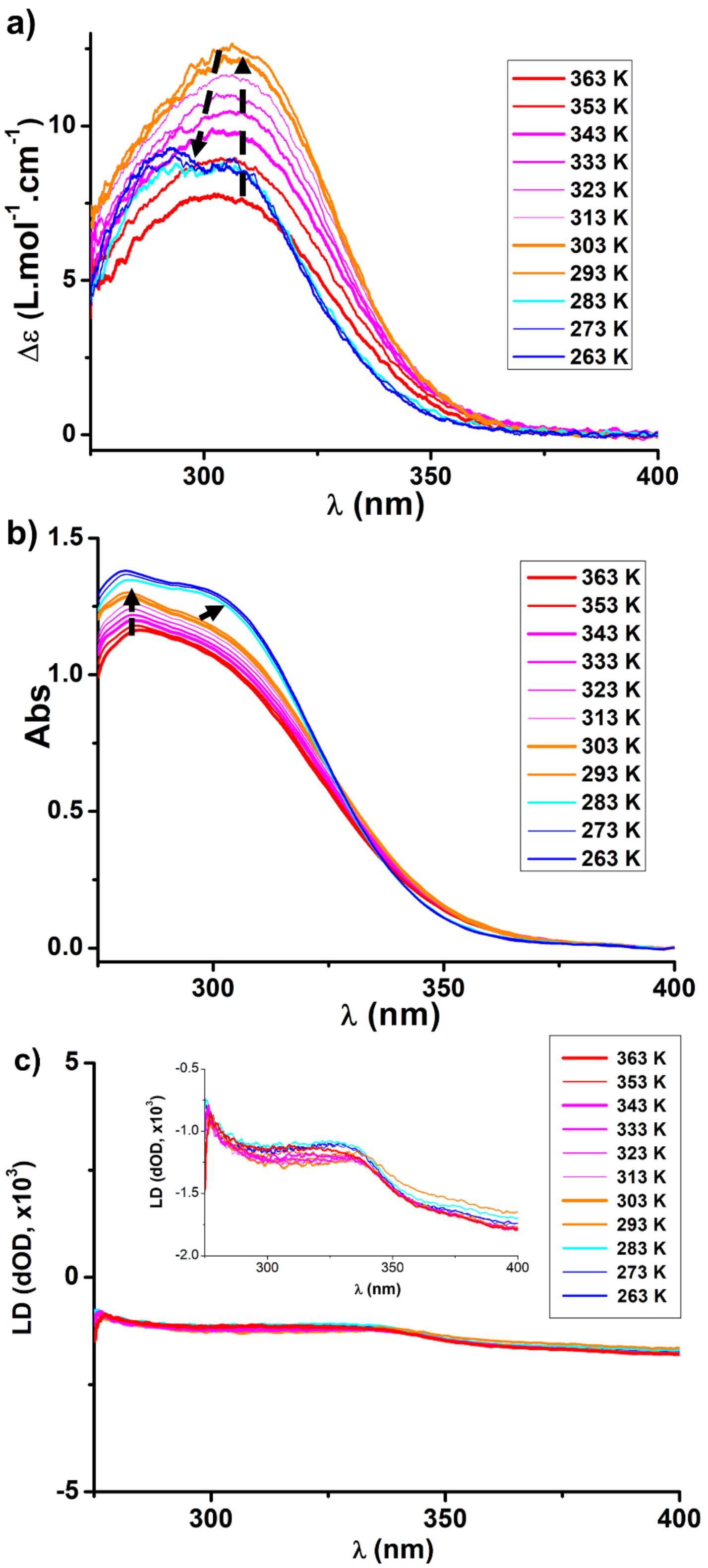

Figure S3. a) CD, b) UV-Vis and c) LD spectra of an 8.5 mM solution of **BTA P*** in toluene recorded at different temperatures (363 K to 263 K, one spectrum every 10 K recorded upon cooling, 1 K.min^{-1}). Arrows help to visualize the evolution of the CD and UV-Vis bands when the temperature decreases.

Interpretation: The transition between the two SP states is well detected by CD and by UV/Vis whilst no significant changes are detected by LD. The overall LD value is very low indicating no contribution to CD spectra.

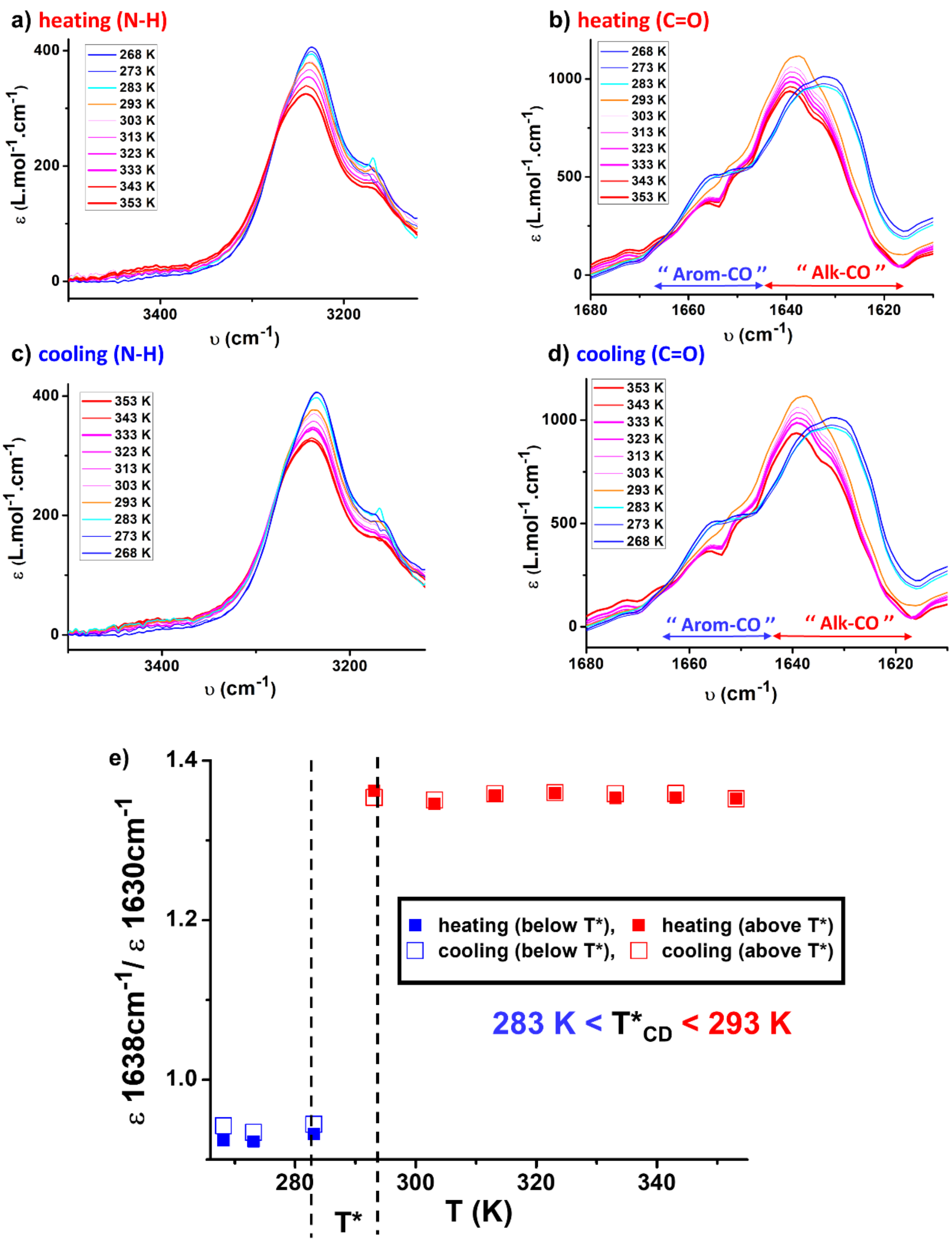

Figure S4. FT-IR spectra of an 8.5 mM solution of **BTA P*** in toluene (and toluene-d$_8$) recorded at different temperatures (268 K-353 K, one spectrum every 10 K except between 273 K and 268 K, 1 K.min^{-1}) recorded upon heating (ab) and cooling (cd). ac) N─H stretching region recorded in toluene and bd) amide C=O stretching region recorded in toluene-d$_8$. The attribution of the regions corresponding to aromatic carbonyl ("Arom-CO", amide CO group connected to the aryl linker) and alkyl carbonyls ("Alk-CO", amide CO groups connected to the (1*S*)-methylheptyl moieties) is made by comparison with the FT-IR spectrum of **BTA*** (see Figure S16b), a BTA monomer which lacks aromatic carbonyl group. e) Plot of the ratio of the ε values at 1638 cm^{-1} and 1630 cm^{-1} as a function of the temperature for the heating and cooling processes.

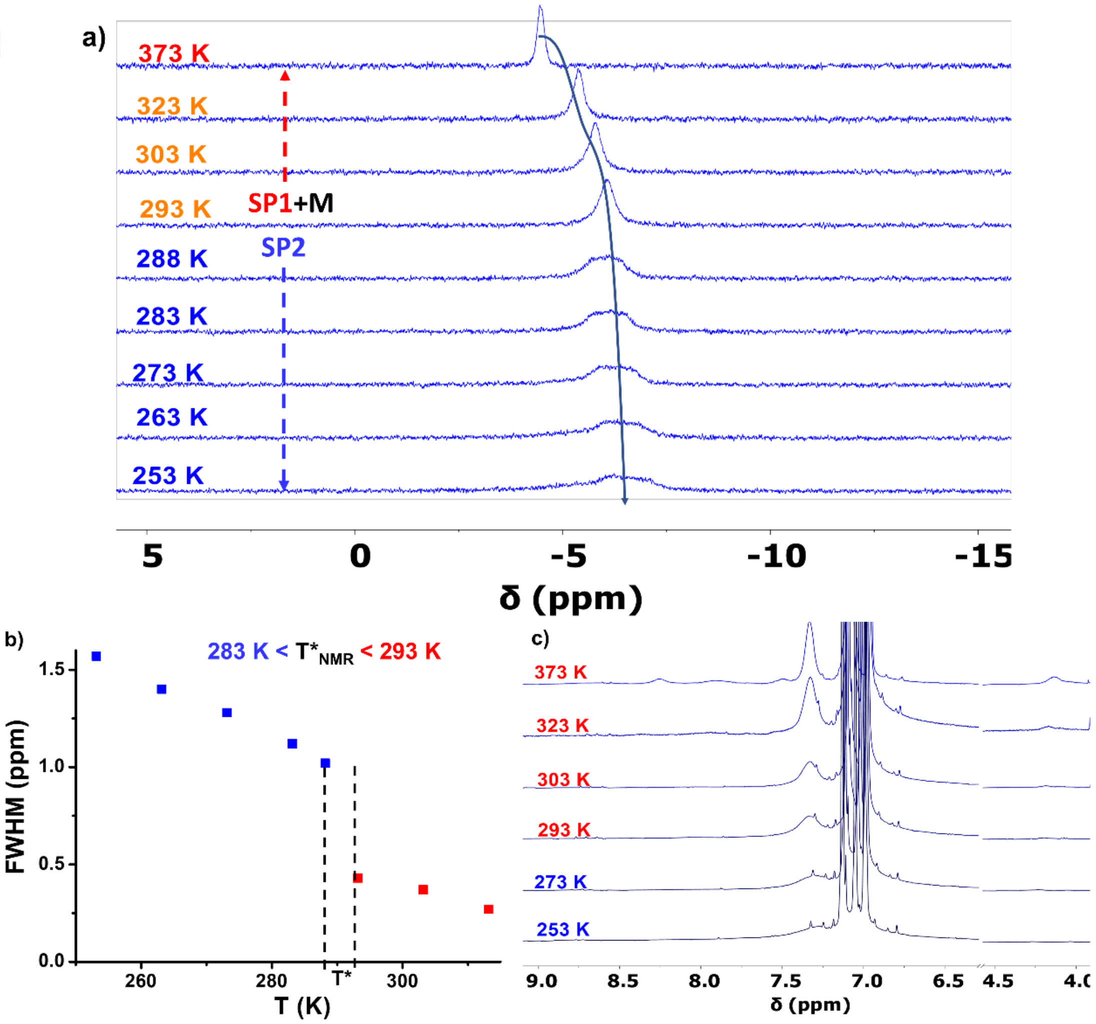

Figure S5. a) $^{31}P\{^1H\}$ NMR spectra as the function of the temperature for a 16.9 mM solution of **BTA P*** in toluene-d_8 (recorded upon cooling). b) Plot of the full width at half maximum (FWHM) of the ^{31}P NMR signal as a function of the temperature. c) ^1H NMR spectra as the function of the temperature for a 16.9 mM solution of **BTA P*** in toluene-d_8 (recorded upon cooling). Zoom on the regions corresponding to aromatic hydrogens, N─H and C─H groups. Below 323 K, only a broad signal corresponding to the diphenylphosphino group is detected; the other signals are not detected because of the polydisperse nature of the SP and their dynamicity.

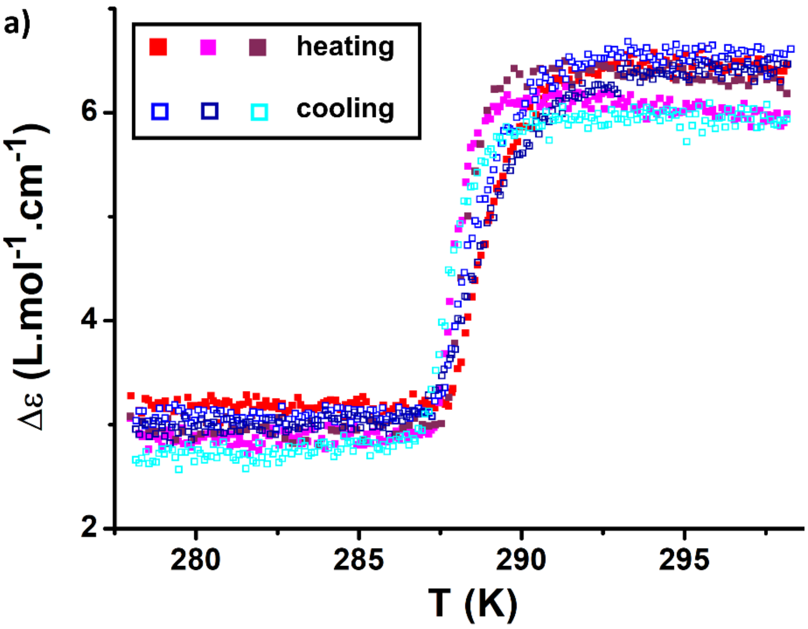

b)

concentration (mM)	T* (K)	σ
16.9	286.93	$1.18{\times}10^{-6}$
8.5	287.86	$1.66{\times}10^{-6}$
5.8	287.60	$6.38{\times}10^{-6}$
4.2	288.14	$2.84{\times}10^{-6}$
1.7	288.53	$2.62{\times}10^{-6}$
0.85	288.36	$5.22{\times}10^{-6}$
0.42	289.12	$6.24{\times}10^{-6}$
0.21	289.20	$9.33{\times}10^{-6}$

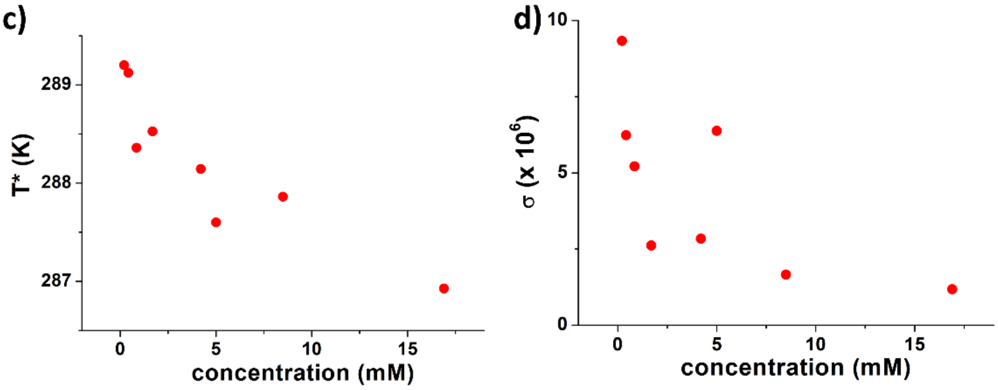

Figure S6. CD intensity (λ= 330 nm, molar CD) as a function of the temperature for an 8.5 mM solution of **BTA P*** in toluene. Heating (solid squares) and cooling (empty squares) processes were repeated between 278 K and 298 K (0.1 K.min^{-1}). An average value of 288.0±0.4 K is obtained for the transition temperature by considering the inflexion point of the curves. b) Transition temperature (T*) and σ values extracted from the fits of the CD curves shown in Figure 2c. c) Plot of the T* versus concentration in **BTA P***. d) Plot of σ values versus concentration in **BTA P***.

Interpretation: The CD curves in a) showed that the transition is reproducible and similar for heating and cooling processes (T*= 288.0±0.4 K). The transition temperature is found to slightly decrease upon increasing the concentration in **BTA P*** (T*= 288.0±1.1 K), yet the change is modest considering that concentration has been changed over two orders of magnitude (c). The cooperativity of the transition is not related to the concentration in **BTA P*** (d, σ= 4±3x10^{-6}).

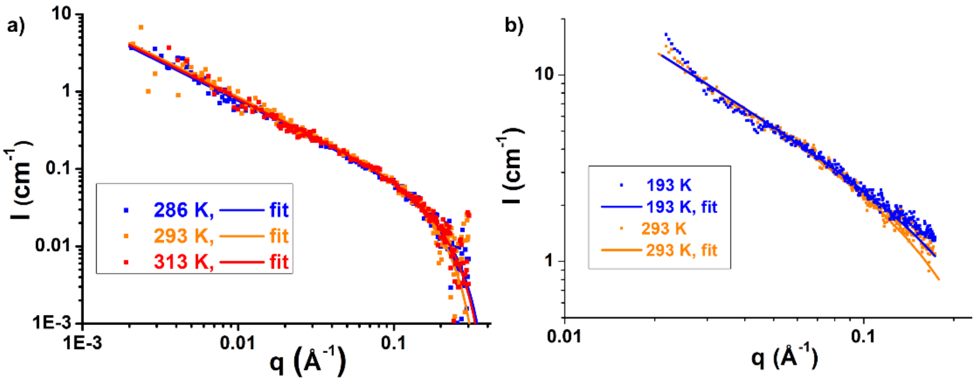

	T (K)	molec / section	radius cross-section (Å)
SANS	286	0.62	8.9
	293	0.69	10.2
	313	0.65	9.2

	T (K)	radius cross-section (Å)
SAXS	193 K	7.0
	293 K	8.7

Figure S7. a) SANS analyses of an 8.7 mM (6.0 g.L^{-1}) solution of **BTA P*** in toluene-d$_8$ at 286 K, 293 K and 313 K with the corresponding fits for rigid rods of infinite length. The extracted radii and number of molecules in the cross-section are indicated in the Table below. b) SAXS analyses of a 16.9 mM (11.7 g.L^{-1}) solution of **BTA P*** in toluene at 293 K and 193 K in the q-range of 0.02 to 0.17 Å^{-1} with the corresponding fits for rigid rods of infinite length. The extracted radii are indicated in the Table below.

Interpretation: The q^{-1} dependency of the scattered intensity is maintained down to the lowest measured q value (SANS data). This indicates that the SP chains are longer than 50 nm which yields a lower estimate value for the DP$_n$ of 70 by considering a distance between **BTA P*** molecules of 3.6 Å in the stacks and a length dispersity of 2. The radii extracted from the SANS and SAXS analyses do not vary significantly as a function of temperature; the radius of the objects is consistent with a single molecule of **BTA P*** present in the cross-section of the rods. These analyses unambiguously discard bundling of the single helices upon lowering the temperature.

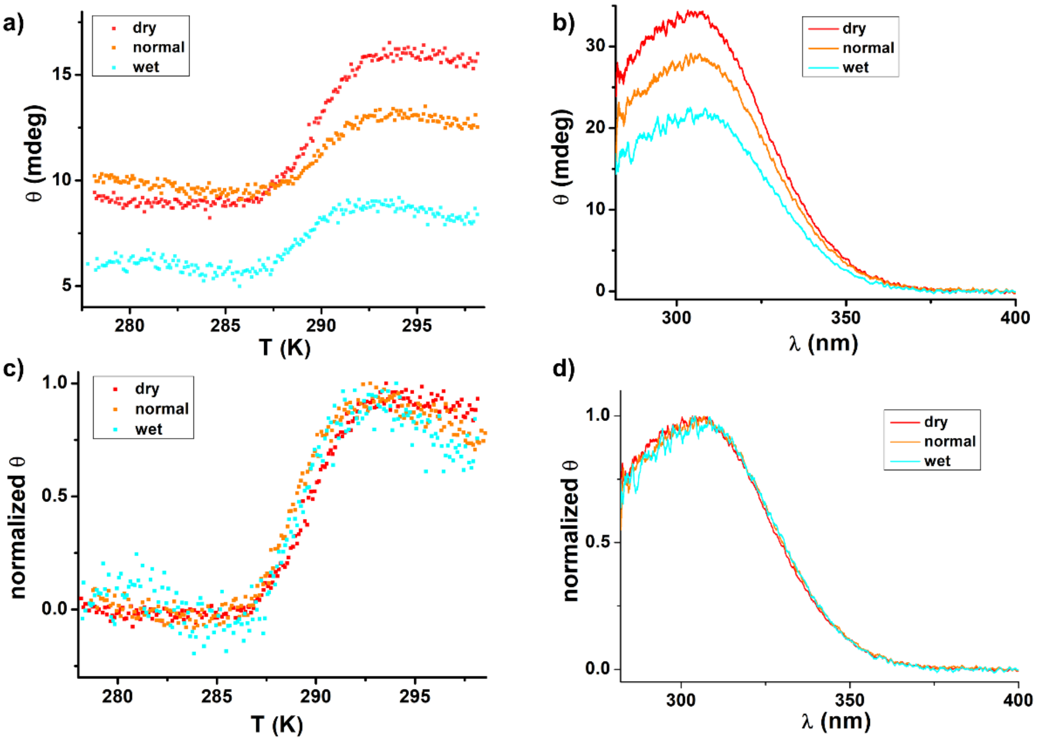

e)

sample name	water (ppm)	ratio (H_2O: **BTA P***)	T* CD (K)
dry	89	0.5:1	289.9
normal	246	1.5:1	290.0
wet	459	2.7:1	289.5

Figure S8. a) CD intensity (λ= 330 nm, mdeg) as a function of the temperature for a 0.21 mM solution of **BTA P*** in toluene containing various amounts of water as indicated in e) (cooling rate: 0.1 $K.min^{-1}$). b) Corresponding CD spectra at 298 K. c) CD plots (from a) normalized at their maxima. d) CD spectra (from b) normalized at their maxima. e) Amount of water in the different samples and the corresponding transition temperatures.

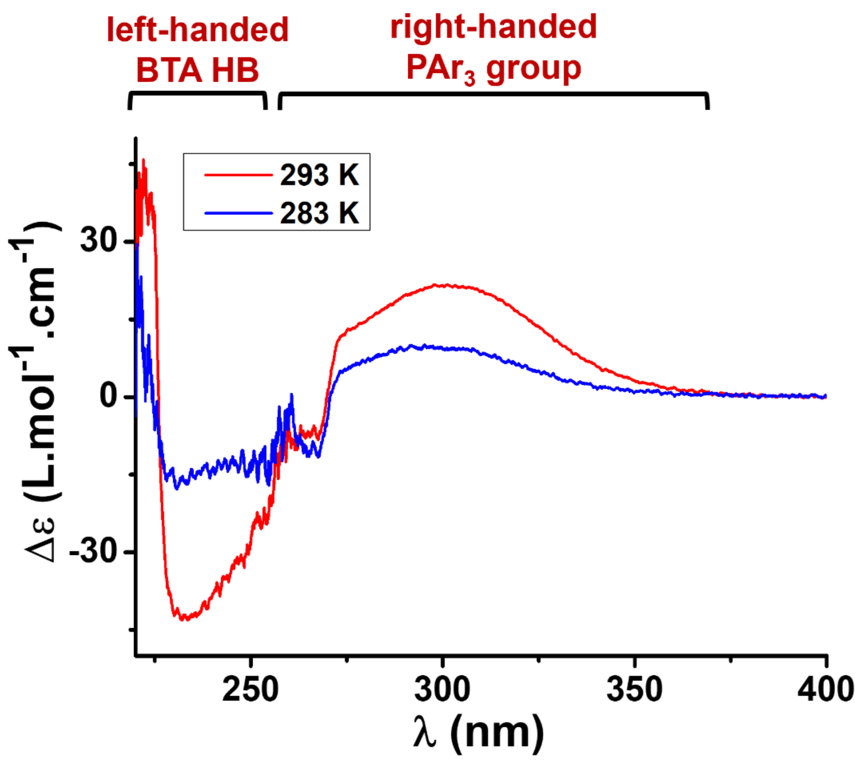

Figure S9. CD spectra of an 8.5 mM solution of **BTA P*** in toluene at 293 K and 283 K. The noise of CD spectra below 280 nm (region where toluene absorbs) was tentatively minimized by increasing the bandwidth to 8 nm. Accordingly, the intensity and shape of the CD signal in that region is not precise. However, it definitively ascertains that both SP states have the same handedness; their hydrogen-bonding (HB) network is left-handed because of the negative CD signal at 230 nm.[3] Both SPs have a positive CD band at *ca.* 300 nm, which belongs to the diphenylphosphino group, which suggests that this group adopts an opposite helical arrangement compared to the hydrogen-bonded network (see the modelling studies for more details).

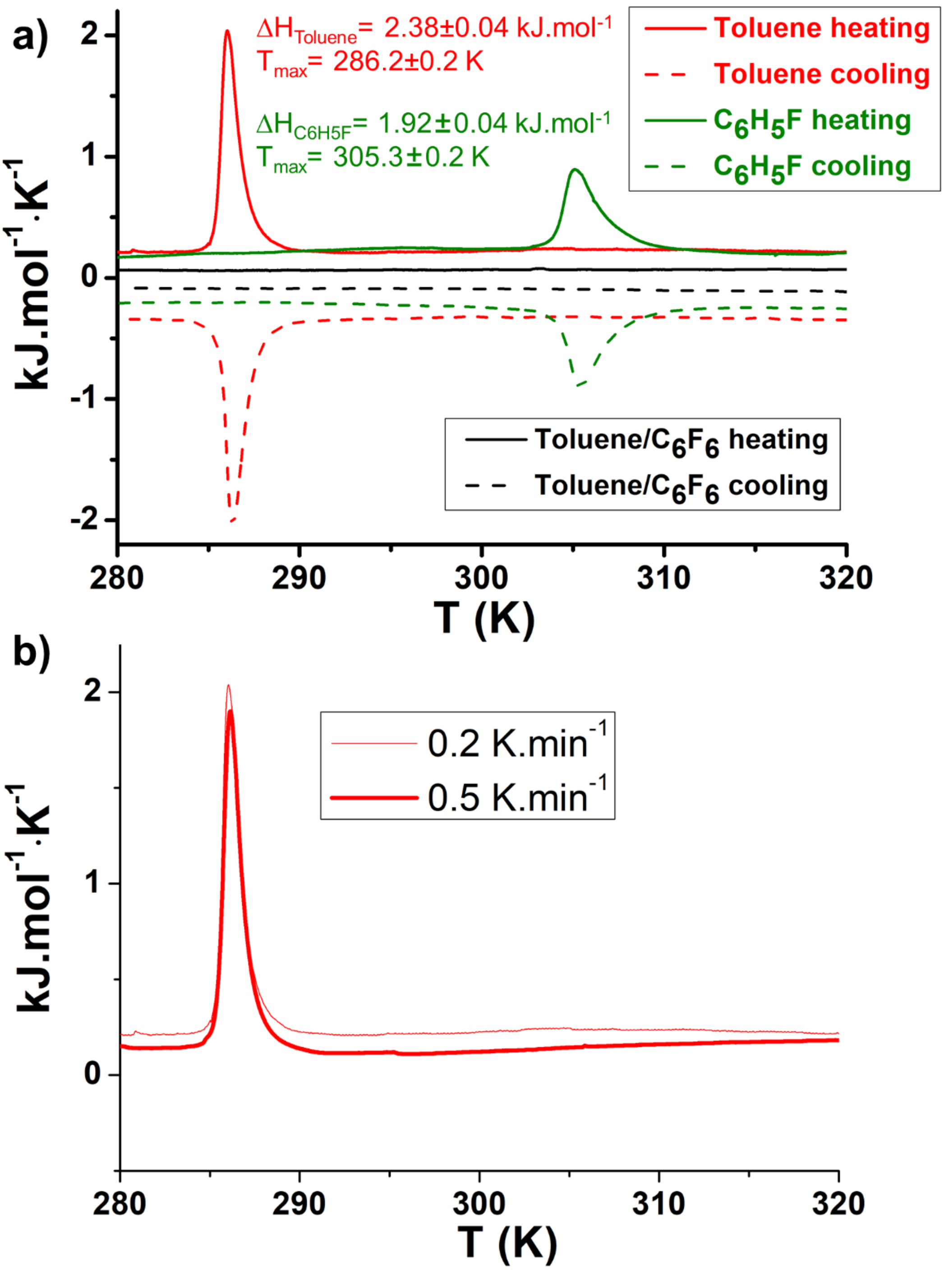

Figure S10. High-sensitivity DSC analyses (0.2 K.min^{-1}) of a 10.0 mM solution of **BTA P*** in toluene, of a 10.0 mM solution of **BTA P*** in fluorobenzene and of a 7.5 mM solution of **BTA P*** in a mixture of toluene: hexafluorobenzene= 90:10 (vol%). Endothermal peaks are directed upward. The transition temperature corresponds to the abscissa at the maximum of the peak whilst the transition enthalpy is obtained upon integration of the peak (average values for the heating and cooling processes). b) High-sensitivity DSC analyses of a 10.0 mM solution of **BTA P*** in toluene at two different heating rates.

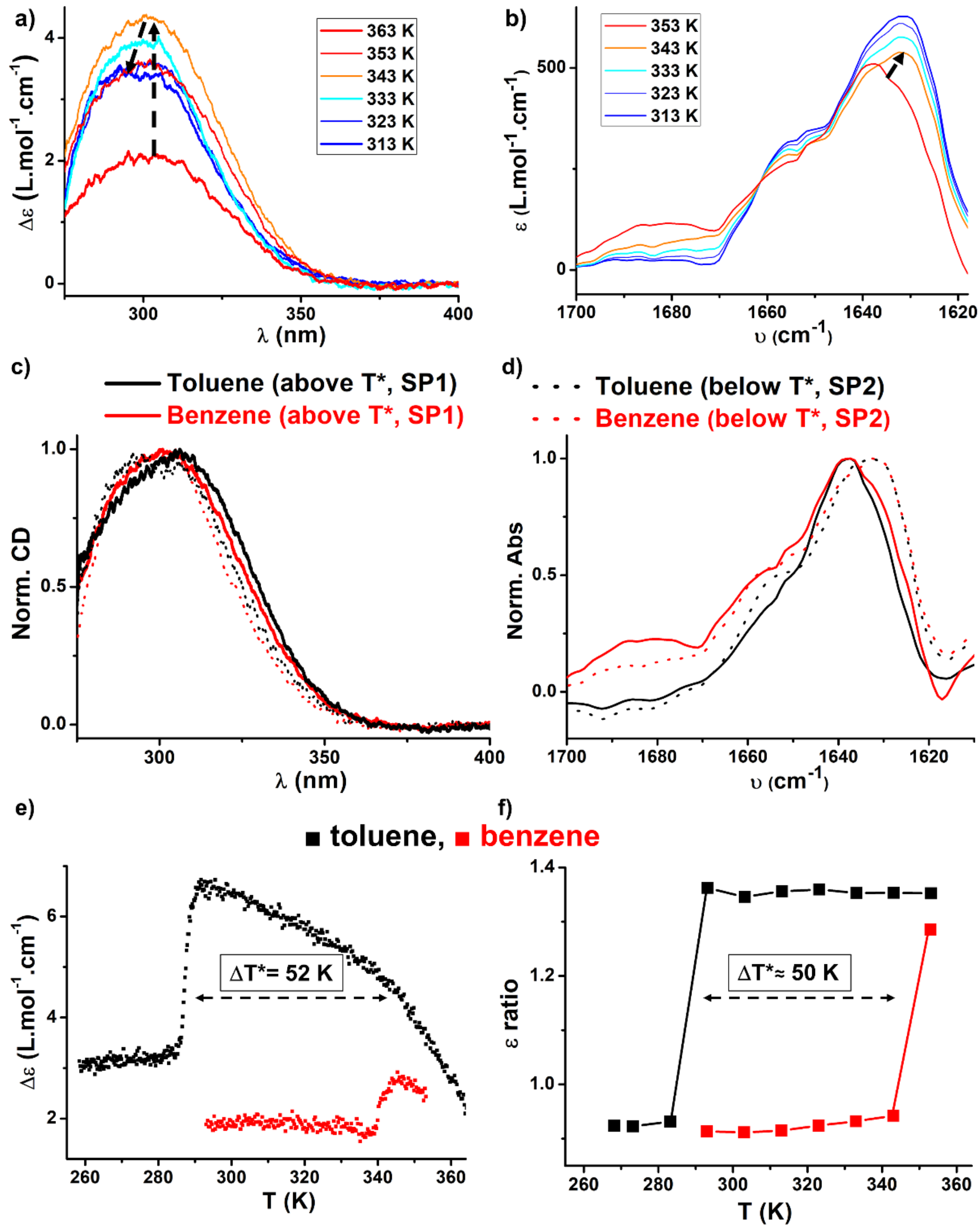

Figure S11. CD (a) and FT-IR (b) spectra of a 5.0 mM solution of **BTA P*** in benzene at different temperatures (313-363 K, one spectrum every 10 K, recorded upon heating, 1K.min^{-1}). Arrows help to visualize the evolution of bands when the temperature decreases. Normalized CD (c) and FT-IR (d) spectra of a 5.0 mM solution of **BTA P*** in toluene and benzene just below and above T*, i.e. 283 K and 293 K for toluene and 333/343 K (CD/FT-IR) and 343/353 K (CD/FT-IR) for benzene. All spectra have been normalized to 1 at their maximum. e) CD intensity (λ= 330 nm, molar CD) as a function of the temperature for 5.0 mM solutions of **BTA P*** in toluene or benzene (cooling rate: 0.5 K.min^{-1}). f) Plot of the ratio of ε values at 1638 cm^{-1} and 1630 cm^{-1} as a function of the temperature extracted from FT-IR data in toluene (Figure S4) and benzene (Figure S11b).

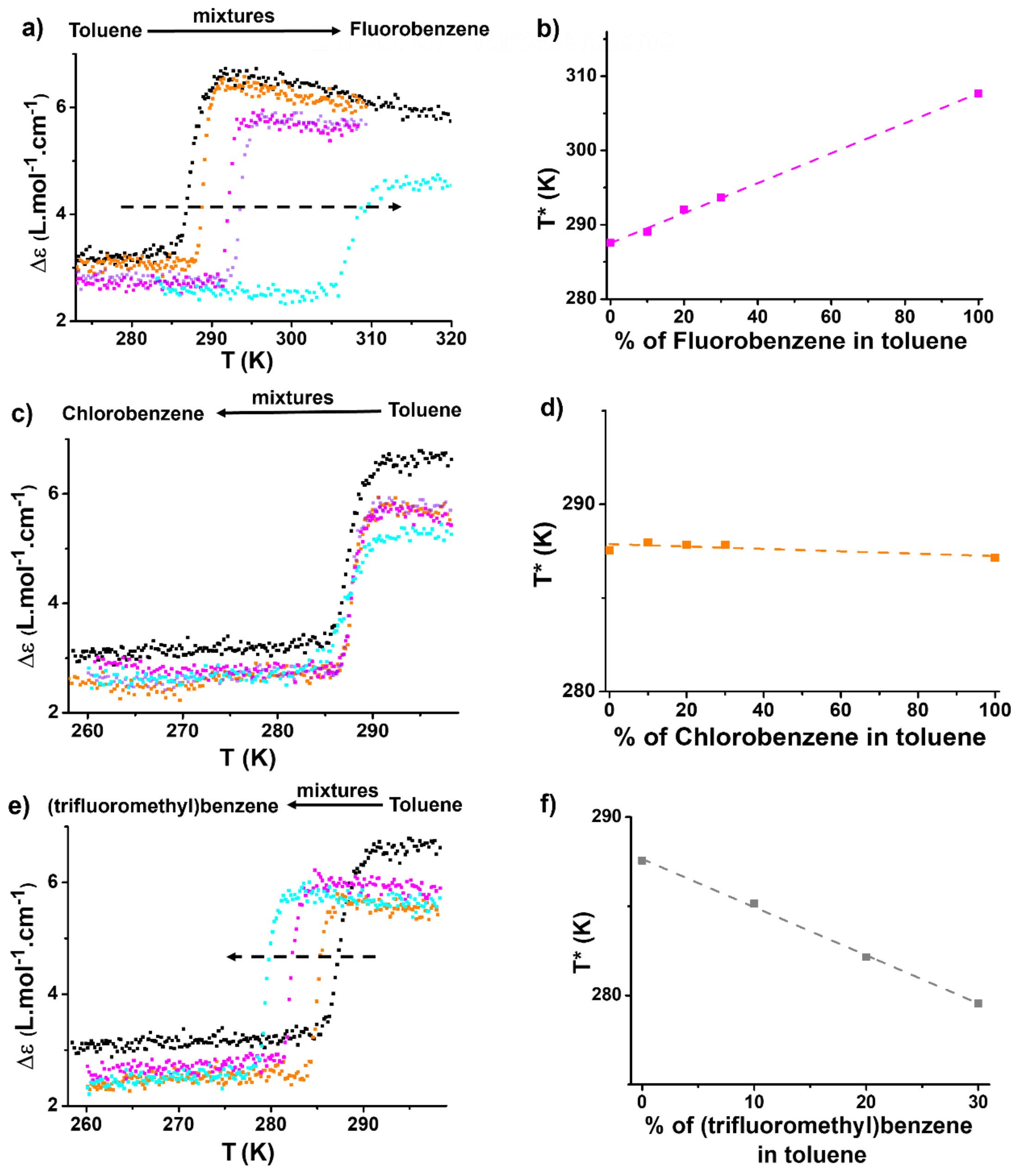

Figure S12. CD intensity (λ= 330 nm, molar CD) as a function of the temperature for 5.0 mM solutions of **BTA P*** in solvent mixtures between toluene and either fluorobenzene (a), chlorobenzene (c) or (trifluoromethyl)benzene (e) with various vol% of cosolvent. Recorded upon cooling (0.5 K.min^{-1}). Plot of the T* values as a function of the vol% of the cosolvent (b, d, f). Data for the mixtures between toluene and benzene mixtures are shown in Figure 5.

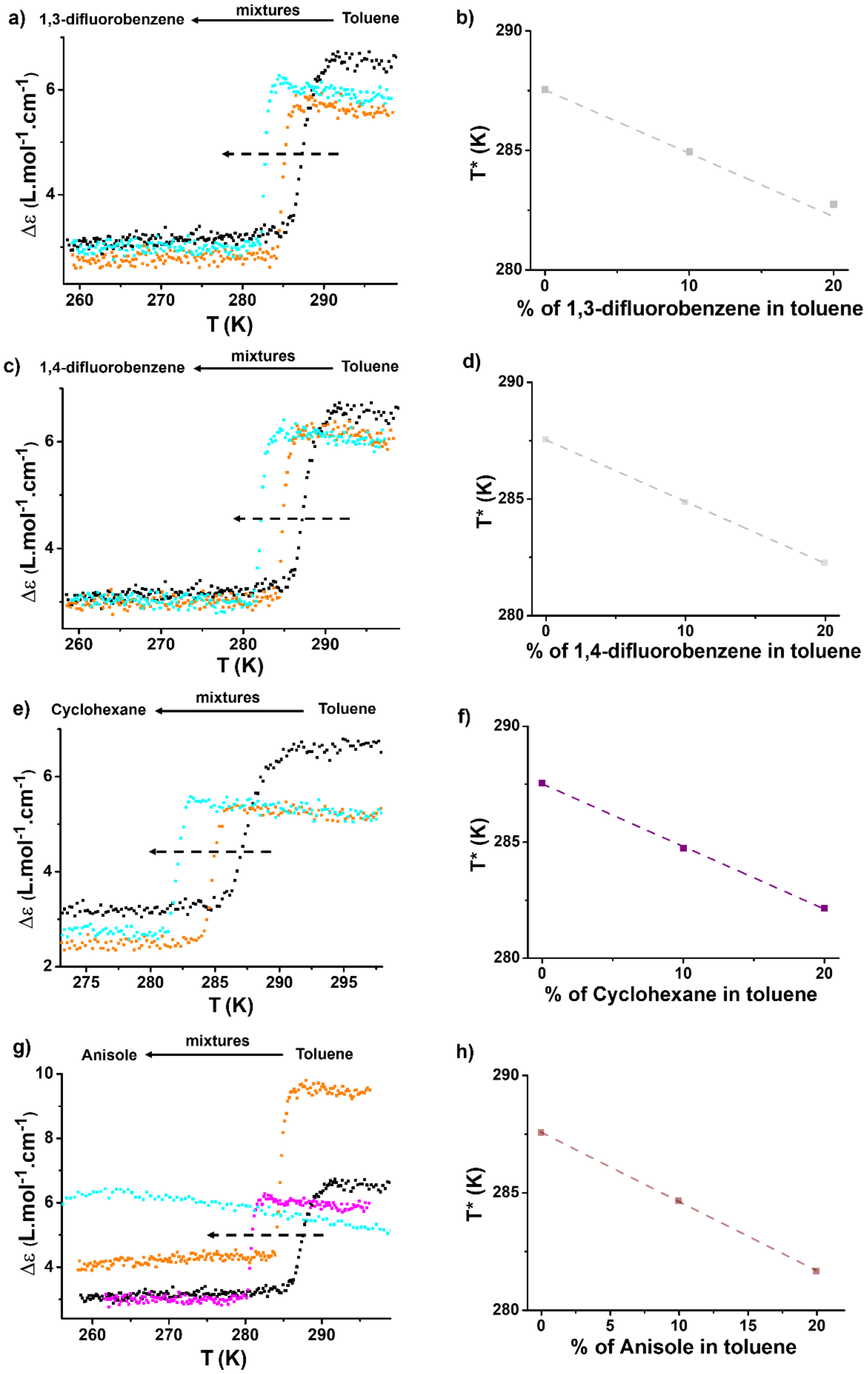

Figure S13. CD intensity (λ= 330 nm, molar CD) as a function of the temperature for 5.0 mM solutions of **BTA P*** in solvent mixtures between toluene and either 1,3-difluorobenzene (a), 1,4-difluorobenzene (c), cyclohexane (e) or anisole (g) with various vol% of cosolvent. Recorded upon cooling (0.5 $K.min^{-1}$). Plot of the T* values as a function of the vol% of the cosolvent (b, d, f, h). The transition could not be detected in pure anisole (g, cyan curve) because it likely occurs below the temperature accessible to our CD setup.

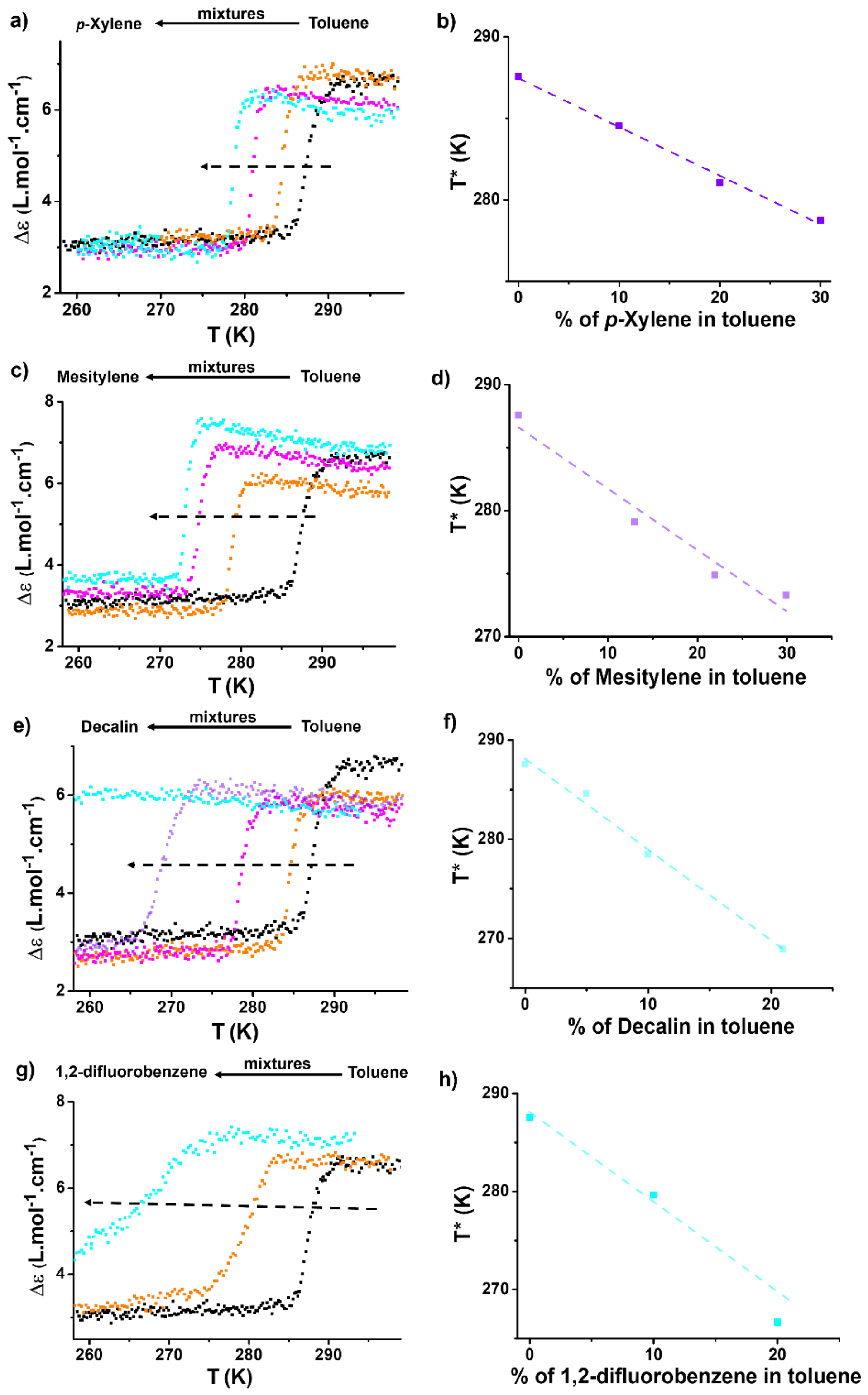

Figure S14. CD intensity (λ= 330 nm, molar CD) as a function of the temperature for 5.0 mM solutions of **BTA P*** in solvent mixtures between toluene and either *p*-xylene (a), mesitylene (c), decalin (e) or 1,2-difluorobenzene (g) with various vol% of cosolvent. Recorded upon cooling (0.5 K.min^{-1}). Plot of the T* values as a function of the vol% of the cosolvent (b, d, f, h). The transition could not be detected in pure decalin (e, cyan curve) because it likely occurs below the temperature accessible to our CD setup. Data for the mixtures between toluene and hexafluorobenzene are shown in Figure 5.

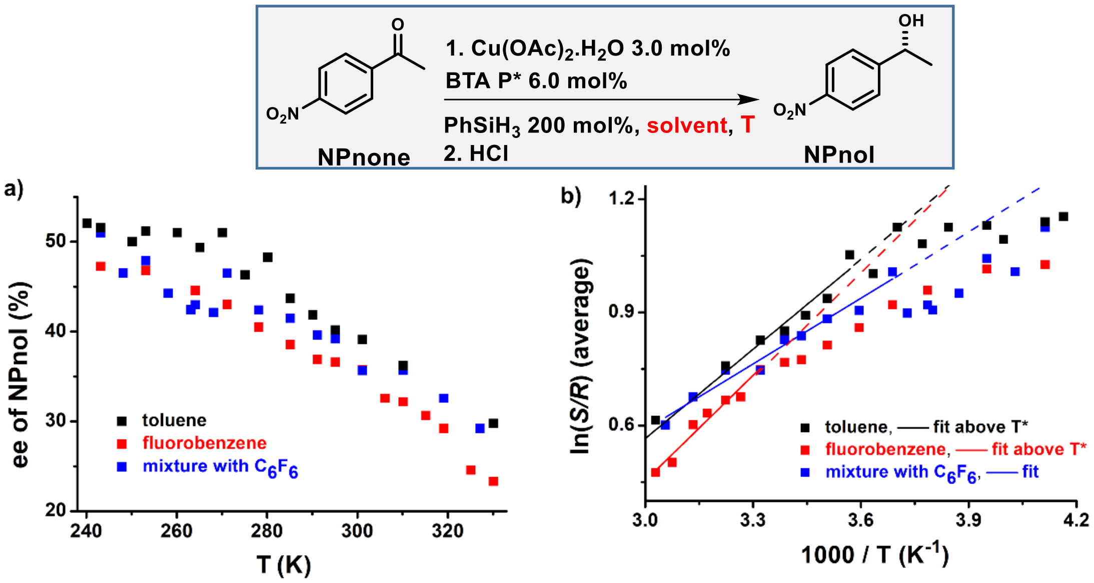

c)

solvent	fit above T* (ΔΔH‡, ΔΔS‡)	fit below T* (ΔΔH‡, ΔΔS‡)
toluene	–6.6±0.3 kJ.mol^{-1}, –15.0±0.9 J.mol^{-1}.K^{-1}	–1.4±0.4 kJ.mol^{-1}, 3.7±1.5 J.mol^{-1}.K^{-1}
fluorobenzene	–7.8±0.5 kJ.mol^{-1}, –19.6±1.5 J.mol^{-1}.K^{-1}	–3.3±0.2 kJ.mol^{-1}, –4.7±0.7 J.mol^{-1}.K^{-1}
mixture with C_6F_6	–4.9±0.3 kJ.mol^{-1}, –9.7±1.1 J.mol^{-1}.K^{-1}	

Figure S15. a) Enantiomeric excess (ee) in **NPnol** as the function of the temperature for the reaction of reference conducted in toluene, in fluorobenzene and in a 90:10 mixture of toluene and hexafluorobenzene (vol%). Conversion>99% was obtained for all catalytic experiments, as determined by GC and ^{1}H NMR analyses. b) Eyring plots for the three solvents. Linear regression is shown only for the high temperature region (above T*). c) Differential enthalpy ($\Delta\Delta H^‡$) and entropy ($\Delta\Delta S^‡$) of activations are extracted from the slope and intercept, respectively, of the fits of the Eyring curves below and above the transition temperature according to the formula: $\ln(S/R) = -\Delta\Delta H^‡/(R\times T) + \Delta\Delta S^‡/R$ (with S/R being the ratio of **NPnol** enantiomers, R the molar gas constant and T the temperature). For the mixture of toluene and hexafluorobenzene, only the data above 270 K can be reasonably fitted.

Interpretation of the catalytic experiments: The Eyring plots for the catalytic data in toluene and fluorobenzene are conveniently fitted with two regression lines, with kinks occurring at temperatures close to the transition temperatures detected in these two solvents (Figure 7). The data obtained for the mixture of toluene and hexafluorobenzene are fitted with a single regression line but a change in the selectivity trend is observed below 270 K, not very far from the transition temperature detected in this solvent mixture (256 K). Regarding the differential energetics between the two diastereomeric transition states (TSs) extracted from the Eyring plots (Figure S15c), it can be deduced that the change of slope is related to a lower and a greater contribution of the differential enthalpy and entropy of activation, respectively, in dictating the selectivity of the SP2 state. In toluene, the contribution of entropy in biasing the relative energy between the two diastereomeric TSs is more important than in fluorobenzene, but a marked reduction in the contribution of enthalpy is observed in both solvents. The energetics governing the TSs above the transition temperature are similar (but not identical) in the three liquids which agrees with a poorly solvated (but not unsolvated) SP1 state. Favoring the SP1 state should in principle lead to higher enantioselectivities at low temperatures since the selectivity for this state is mostly driven by the difference in enthalpies between the diastereomeric TSs (see the equation in the caption of Figure S15). It should be in principle the case for the toluene/hexafluorobenzene mixture but the positive effect of lowering the temperature is counterbalanced by a decrease of the selectivity close to the transition temperature.

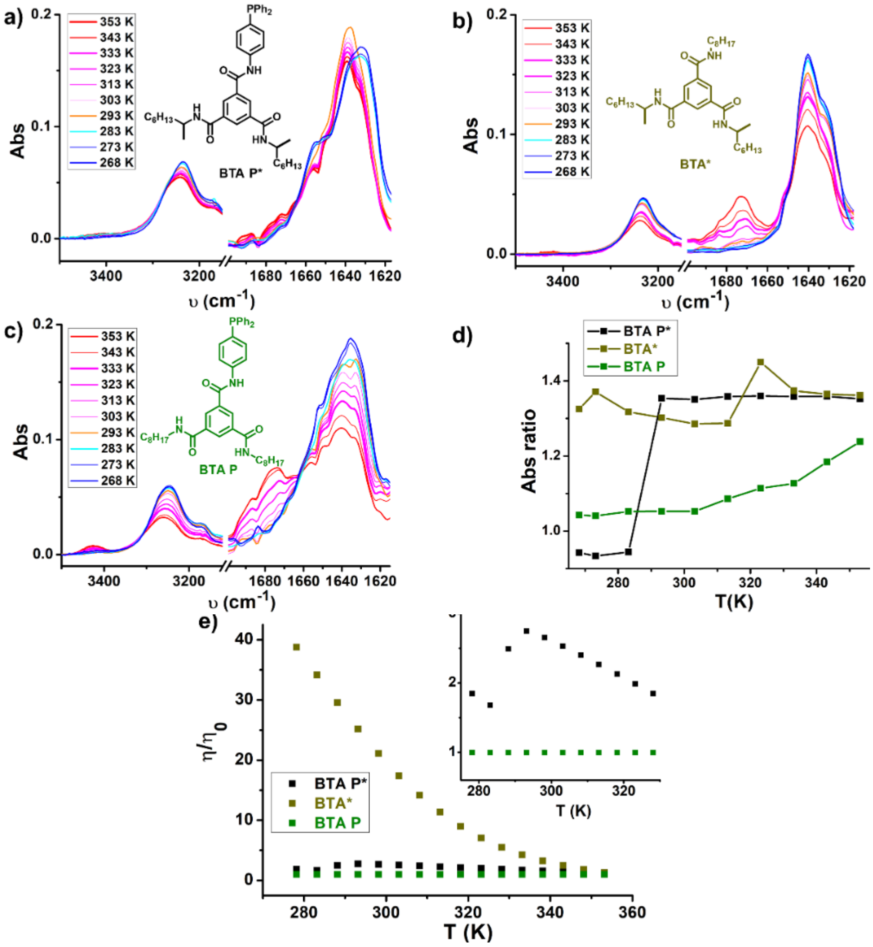

Figure S16. FT-IR spectra of an 8.5 mM solution of either **BTA P*** (a), **BTA*** (b) or **BTA P** (c) in toluene (and toluene-d$_8$) recorded upon cooling at different temperatures (268-353 K, one spectrum every 10 K except between 273 K and 268 K, 1 K.min^{-1}). N─H stretching region recorded in toluene and amide C=O stretching region recorded in toluene-d$_8$. d) Plot of the ratio of the absorbance values at 1638 cm^{-1} and 1630 cm^{-1} as a function of the temperature for the three BTA monomers. e) Plot of the relative viscosities as a function of the temperature for solutions of either **BTA P***, **BTA*** or **BTA P** in toluene (1.0 mM). Recorded upon cooling from 353 K to 278 K. The viscosity of the **BTA*** solution is far higher than the other, yet it does not display thermothickening as expected from the absence of structural transition.

Interpretation of the FT-IR data: The structural transition, associated with a sudden change in the shape and position of the amide I band is observed exclusively for **BTA P***. It translates into a sudden decrease of the ratio of the absorbances at 1638 cm^{-1} and 1630 cm^{-1} upon lowering the temperature (Figure S16d). Increasing the temperature leads to a higher proportion of free C=O functions for **BTA P** and **BTA***, highlighting the higher stability of **BTA P*** relatively to **BTA P** and **BTA***. Further examination of the amide I band indicates that the shoulder which is characteristic of the SP2 state of **BTA P*** (≈ 1655 cm^{-1}) can mostly be attributed to the carbonyl group connected to the diphenylphosphino group through an aryl linker (aromatic C=O) since carbonyl groups connected to alkyl chains (alkyl C=O) are mostly located below 1650 cm^{-1} (as deduced by comparison with the FT-IR signal displayed by **BTA***). This suggests a more regular organization of the carbonyl groups for **BTA P*** monomers in the SP2 state, maybe though exclusive binding between the aromatic and alkyl amide functions (versus mixture of aromatic/aromatic and aromatic/alkyl in the SP1 state) as represented in the modelled hexadecamer in Figure 8b.

Interpretation of the viscosity data: The viscosity of the **BTA*** solution is far higher than the other, as a possible consequence of easier crosslinking between SP chains through interactions between its three alkyl side chains. However, it does not display thermothickening (insert of Figure S16e) as expected from the absence of a structural transition.

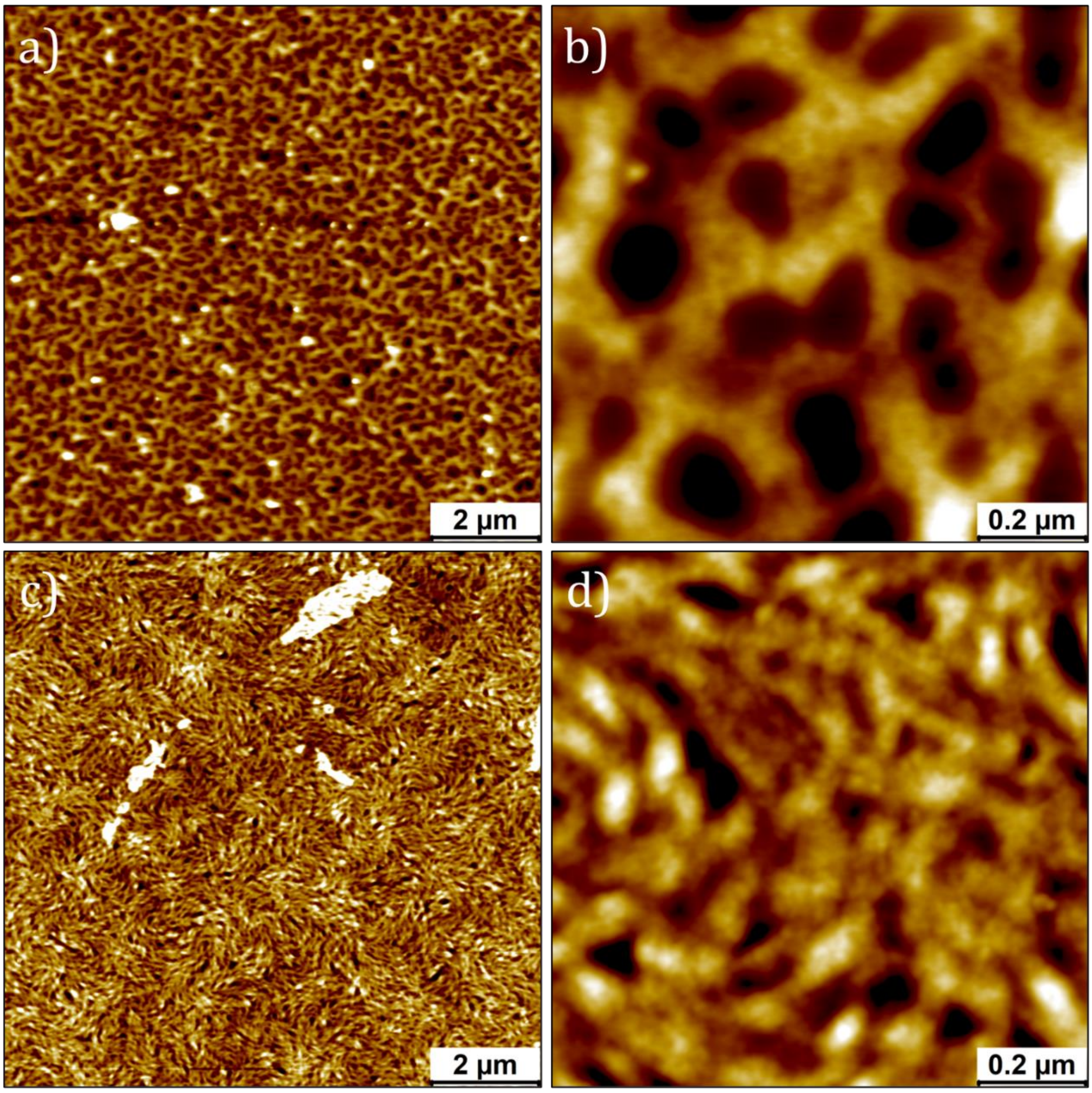

Figure S17. Topography AFM images of **BTA P*** fibers on a silica wafer. AFM images, (a) large-scale, and (b) small-scale, of fibers formed from a 2.0 mM solution of **BTA P*** in toluene. Similarly, images (c) and (d) display large- and small-scale views of fibers formed from a 2.0 mM solution in fluorobenzene. All samples were prepared by spin-coating five drops of the 2.0 mM solutions onto a silica wafer for 2 minutes at 293 K.

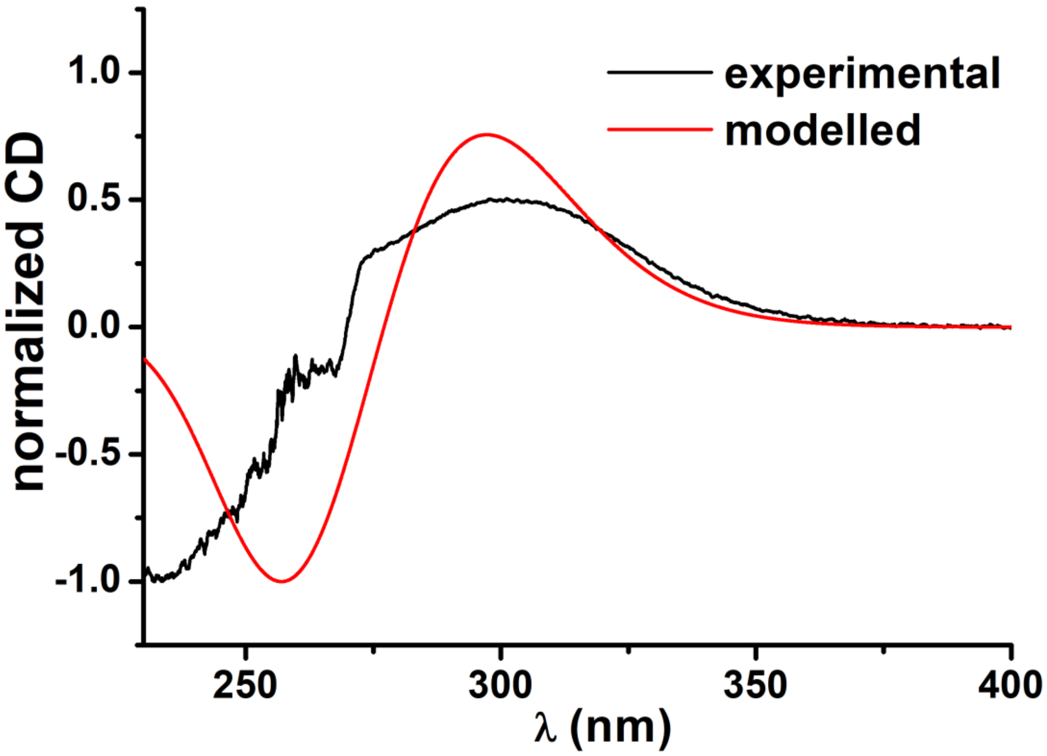

Figure S18. Comparison of the experimental and modelled CD spectra of **BTA P*** assemblies. The experimental spectrum has been recorded in toluene at 293 K (see Figure S9). The modelled spectrum was obtained from the octamer of a modified version of **BTA P*** with (*S*) sec-butyl side chains as explained in the Methods section below.

Interpretation: The positive CD couplet at low energy observed for **BTA P*** assemblies in solution is well reproduced by the CD spectrum of the computed structure which possesses an opposite helical arrangement between the internal hydrogen-bonding groups and the peripherical appended diphenylphosphino moieties.

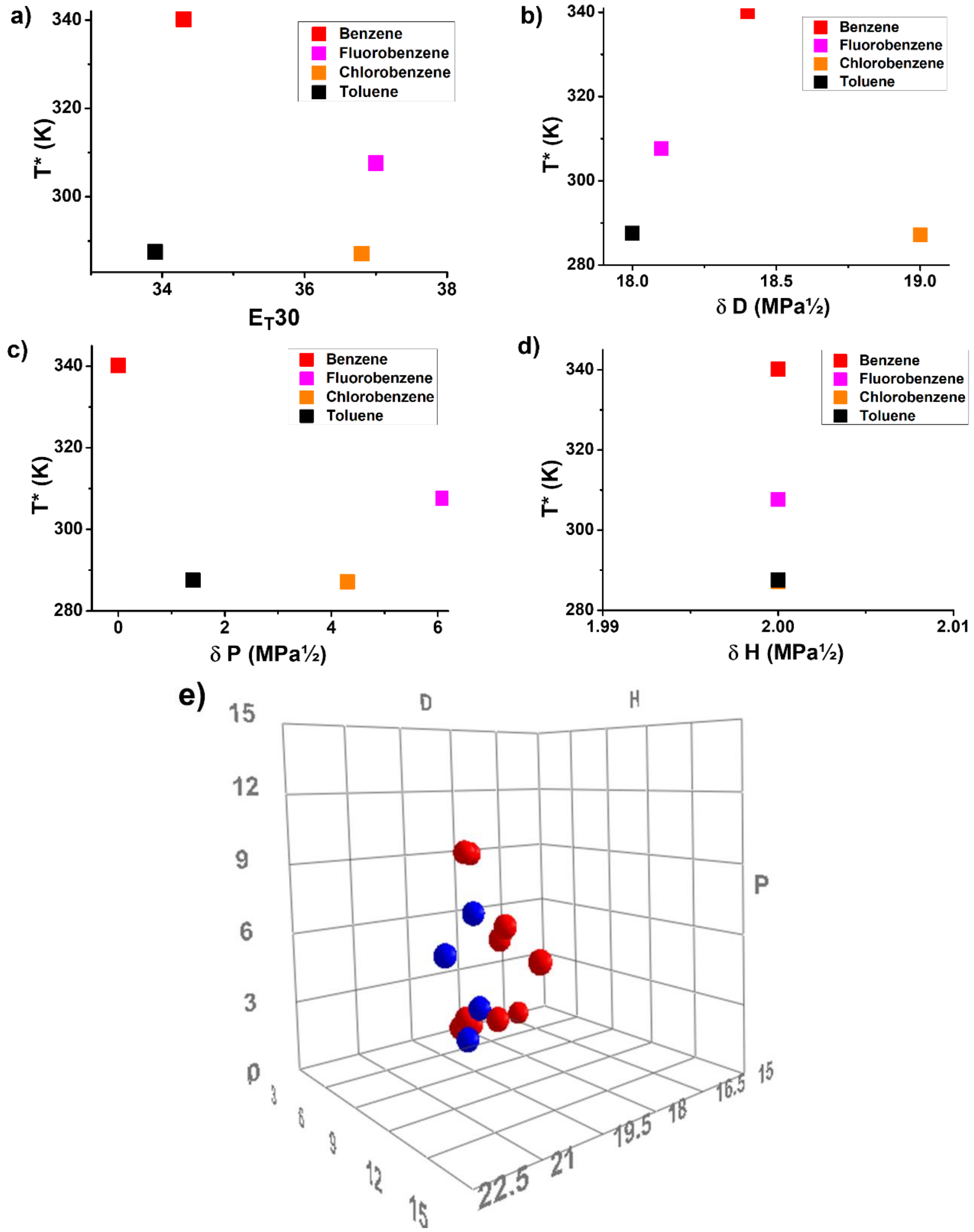

Figure S19. Plot of the T* values obtained for **BTA P*** solutions in pure solvents as a function of E_T30 (a), of the dispersion term of HSP (b), of the polar term of HSP (c) and of the hydrogen bond term of HSP (d). e) Solvents, for which the transition of **BTA P*** has been detected, are positioned in the Hansen space. Blue: solvents for which the T* is above or equal to that observed in toluene (toluene, chlorobenzene, benzene, fluorobenzene). Red: solvents for which the T* is below 286 K (1,2-difluorobenzene, 1,3-difluorobenzene, 1,4-difluorobenzene, methylcyclohexane, anisole, hexafluorobenzene, *p*-xylene, (trifluoromethyl)benzene, mesitylene and decalin). For solvent mixtures, only the HSP corresponding to the cosolvent has been considered.

Interpretation: There is no correlation between the transition temperature and the E_T30 value of the solvents, the individual terms of the HSP of the solvents and the position of the solvents in the HSP space.

**Solvent**	T*(K) 90:10 mixture	T* (K) 80:20 mixture	δD (MPa½)	δP (MPa½)	δH (MPa½)	Molar Volume (MVol) (cm^3/mol)
benzene	292.8	297.2	18.4	0	2.0	89.5
fluorobenzene	288.9	290.3	18.1	6.1	2.0	94.3
chlorobenzene	287.9	287.9	19.0	4.3	2.0	102.1
toluene[(a)]	287.6	287.6	18.0	1.4	2.0	106.6
1,2-difluorobenzene	285.6	283.6	18.0	9.0	1.0	98.5
1,3-difluorobenzene	285.1	282.8	17.7	4.9	3.2	99.0
1,4-difluorobenzene	284.9	282.3	17.9	5.7	4.3	98.7
cyclohexane	284.8	282.1	16.8	0	0.2	108.9
(trifluoromethyl)benzene	284.8	281.5	17.5	8.8	0	122.9
anisole	284.6	281.6	17.4	4.4	6.9	109.2
p-xylene	284.1	280.7	17.8	1.0	3.1	121.1
mesitylene	279.2	270.9	18.0	0.6	0.6	139.5
decalin[(b)]	279.0	262.0	17.8	0	0	157.0
hexafluorobenzene	253.7	219.8	16.0	0	0	115.8

Table S2. Solvent parameters provided by HSPiP.[4] Solvents are ranked according to the T* (descending) obtained for **BTA P*** solutions in pure toluene or in toluene/cosolvent mixtures (10 mol% and 20 mol% of cosolvent). The indicated solvent parameters are those of the cosolvent. (a) Values are those for toluene. (b) Average values between the *trans* and *cis* isomers.

**Solvent**	van der Waals[a]					Accessible surface[b]				
	Area / $Å^2$	Volume / $Å^3$	Ovality[c]	Max length / Å	Max radius / Å	Area / $Å^2$	Volume / $Å^3$	Ovality[c]	Max length / Å	Max radius / Å
benzene	104.3	79.6	1.17	7.2	3.6	306.9	474.7	1.04	11.2	5.6
fluorobenzene	111.1	84.9	1.19	7.8	4.1	318.1	498.0	1.05	11.7	6.1
chlorobenzene	121.4	94.0	1.21	8.4	4.5	335.8	534.9	1.05	12.4	6.5
toluene	123.6	94.7	1.23	8.2	4.1	339.2	541.4	1.06	12.2	6.1
(trifluoromethyl) benzene	144.0	111.0	1.29	8.7	5.2	369.1	606.5	1.07	12.7	7.1
1,3-difluorobenzene	118.1	90.3	1.21	7.8	4.0	330.0	522.3	1.05	11.8	6.0
1,4-difluorobenzene	118.1	90.3	1.21	8.3	4.2	330.1	522.5	1.05	12.3	6.2
cyclohexane	123.3	93.1	1.24	7.2	3.6	331.6	532.8	1.04	11.2	5.6
anisole	134.4	103.6	1.26	9.3	4.8	357.4	578.3	1.06	13.3	6.8
p-xylene	143.3	110.0	1.29	9.2	4.6	372.4	609.6	1.07	13.2	6.6
mesitylene	162.8	125.1	1.35	8.8	4.6	405.0	677.1	1.09	12.8	6.6
decalin	181.7	145.7	1.36	9.5	4.8	414.7	725.6	1.06	13.5	6.8
1,2-difluorobenzene	118.2	90.3	1.21	7.8	4.2	329.0	521.0	1.05	11.8	6.3
hexafluoro benzene	146.0	112.0	1.30	8.4	4.2	370.0	610.0	1.06	12.4	6.2

a: The van der Waals (vdw) surface is generated by substituting every atom with spheres having van der Waals radius.
b: The accessible surface is the locus of the center of a small solvent sphere (2.0 Å radius) tracing the vdw surface.
c: Ovality is defined as molecular surface area divided by minimum surface area, where minimum surface area is the surface area of a (complete) sphere having the identical volume.

Table S3. Molecular areas and volumes for the pure solvents computed by Winmostar software. Every structure was optimized by Gaussian with B3LYP/6-31g(d) level.

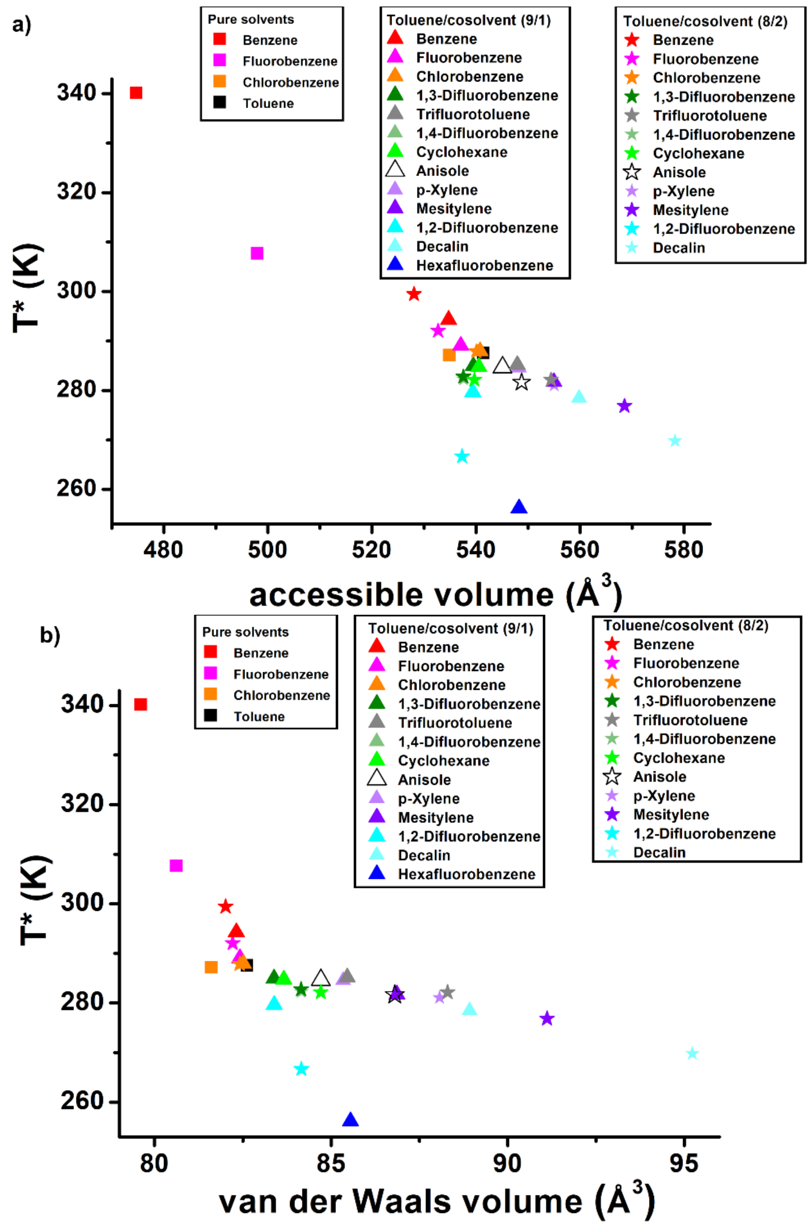

Figure S20. Plot of the T* values obtained for **BTA P*** solutions in pure solvents and toluene/cosolvent mixtures as a function of the accessible volume a) or the van der Waals volume b). The volumes of the mixture are obtained by the weight average of the volumes of the pure solvents.

Interpretation: The transition temperature appears to be reasonably correlated with the solvent volume, a point valid for both aromatic and aliphatic solvents (such as cyclohexane and decalin). Hexafluorobenzene (blue triangles) is a clear outlier: the measured T* is lower than expected relative to the proportion of this solvent in the mixture. This suggests that this solvent has a lower affinity for the PAr_3 group than the other studied solvents; electronic repulsion between these two electron-poor aromatic rings likely leads to a less favorable solvation enthalpy relative to solvents with neutral or electron-rich aromatic rings. Regarding the difluorobenzene isomers, 1,2-difluorobenzene is the only one that seems to be out of the overall trend, suggesting a lower solvation than expected for this solvent maybe because of a geometrical mismatch with the lateral diphenylphosphino group. Finally, solvents with electron-rich aromatic rings are expected to have a higher electronic affinity for PAr_3; however, mesitylene and anisole mixtures follow the general trend inferring that electronics are counterbalanced by steric hindrance in these cases.

Materials and methods (Figure S21)

Materials: The synthesis of **BTA P**[5] was reported previously. **BTA*** was prepared following a protocol reported for similar structures in the literature.[6] DMAP was purchased from Sigma-Aldrich and used as received. $PhSiH_3$ (>97%) and $[Cu(OAc)_2{\cdot}H_2O]$ (>99%) were purchased from Alfa Aesar and used without any purification. EDC·HCl was purchased from Fluorochem. DMSO-d_6 and toluene-d_8 were purchased from Eurisotop and used without further purification. Anhydrous DCM, THF and toluene were obtained from a solvent purification system (IT-Inc).

<u>Solvents:</u> tetralin (>99.5%, Sigma-Aldrich), *iso*-octane (>99%, Sigma-Aldrich), methylcyclohexane (>99%, Sigma-Aldrich), *n*-octane (>98%, Alfa Aesar), *n*-decane (>99% GC, TCI Europe), 1-chlorooctane (>99%, Alfa Aesar), butylcyclohexane (>99%, Sigma-Aldrich), *n*-dodecane (>99%, Sigma-Aldrich), 2,2,4,4,6,8,8-heptamethylnonane (>98%, Sigma-Aldrich), benzene (99.5% GC, TCI Europe), fluorobenzene (>99%, Sigma-Aldrich), chlorobenzene (>99.8%, Sigma-Aldrich), (trifluoromethyl)benzene (>99%, Sigma-Aldrich**)**, 1,3-difluorobenzene (>99%, Sigma-Aldrich), 1,4-difluorobenzene (>99%, Sigma-Aldrich), cyclohexane (>99%, Sigma-Aldrich), anisole (>99%, Sigma-Aldrich), *p*-xylene (>99%, Sigma-Aldrich), mesitylene (>98%, Sigma-Aldrich), decalin (mixture of *cis* + *trans*, >98%, Sigma-Aldrich), 1,2-difluorobenzene (>98%, Sigma-Aldrich), hexafluorobenzene (>99.99%, ABCR).

Purification by flash chromatography was performed by adsorbing the samples on silica; the adsorbed samples were introduced into the solid loader and purified by means of a Reveleris X2 purification system (Buchi®) using pre-packed silica cartridges Ecoflex® (irregular 50 µm silica) of 40 g. ^{1}H NMR spectrum was recorded on a Brucker 400 Avance and is calibrated with residual DMSO-d_6 protons signals at δ=2.50 ppm. ^{13}C NMR and ^{31}P NMR spectra were recorded on a Brucker 400 Avance. Data are reported as follows: chemical shift (δ ppm), multiplicity (d= doublet, m= multiplet), coupling constant (Hz) and integration. Exact mass measurements (HRMS) were obtained on a TQ R30-10 HRMS spectrometer by ESI^+ ionization and are reported in *m/z* for the major signal. FT-IR analysis of the solid of **BTA P*** was performed by drop coasting its solution over a KBr plate. The main peaks were reported as m: medium, s: strong, w: weak, br: broad. A double wall glass beaker filled with PDMS (Kryo 90 from Lauda®) connected to cryostat Proline RP890 from Lauda® was used to perform the catalytic reactions below 293 K, and oil bath was used to perform the catalytic reactions above 293 K.

Methods:

<u>Chiral HPLC analyses:</u> The optical purity of **BTA P*** (ee> 99%) was determined by analytical HPLC (Chiralpak IE, heptane/ethanol (90/10) as mobile phase, 1 mL/min). See Figure S26.

<u>Fourier-Transform Infrared (FT-IR) analyses</u>: FT-IR measurements were performed on a Nicolet iS10 spectrometer. Spectra of solutions in toluene, benzene and

mixture of toluene and hexafluorobenzene were measured in 0.2 mm (Figures 2d, 4c, 8a, S4, S11 and S16) and in 1.0 mm (Figures S1a), pathlength CaF_2 cells and were corrected for air, solvent and cell absorption at the same temperature. For variable-temperature FT-IR measurements, the temperature was controlled with a digital temperature controller (West 6100+) from Specac. Full spectra were measured every 10 °C (heating/cooling rate: 1 K/min). Below 293 K, liquid nitrogen and ethylene glycol were utilized for slow cooling while vacuum was applied to prevent condensation. Thermal expansion of the solutions was not corrected. For FT-IR measurements in toluene, the N-H region was recorded in toluene, while the C=O region was recorded in toluene-d_8 in order to get good quality spectra.

<u>Circular dichroism (CD) analyses</u>: CD measurements were performed on a Jasco J-1500 spectrometer equipped with a Peltier thermostated cell holder and a Xe arc lamp. Data of Figures 2a, S3 and S6b were recorded from 363 K to 263 K upon cooling process, Figures 4a were recorded from 313 K to 363 K upon heating process and Figures 1a and S8b, were recorded at 293 K, with the following parameters: 50 nm·min^{-1} sweep rate, 0.05 nm data pitch, 2.0 nm bandwidth with solutions placed into cylindrical spectrosil quartz cells of 0.01 mm, 0.10 mm, 0.20 mm or 5.0 mm pathlength (Starna® 31/Q/0.01, Starna® 31/Q/0.1, Starna® 31/Q/0.2 and Starna® 31/Q/5.0 respectively). Data of Figure S9 were recorded at 293 K and 283 K with the following parameters: 50 nm·min^{-1} sweep rate, 0.05 nm data pitch, 8.0 nm bandwidth, and between 400 nm and 220 nm with solutions placed into dismountable quartz cells of 0.01 mm pathlength. For variable-temperature CD experiments (Figures 1cd, 2bc, 4b, 5a, S3, S6, S8a and S12-S14), solutions were placed into cylindrical spectrosil quartz cells of 0.10 mm, 0.20 mm, 0.5 mm, 1 mm, 2 mm, 5 mm or 10 mm pathlength (Starna® 31/Q/0.1, 31/Q/0.2, Starna® 31/Q/0.5, Starna® 31/Q/1.0, Starna® 31/Q/2.0, Starna® 31/Q/5.0 or Starna® 31/Q/10.0 respectively), heated to 373 K and the ellipticity was recorded at λ=330 nm during a cooling and a heating process (0.5, 0.3 or 0.1 K.min^{-1}, see caption of the corresponding figures). All solutions were pre-heated before measurements. Toluene and cell contributions at the same temperature were subtracted from the obtained signals. The temperature in the cylindrical (closed) cells can be higher than the boiling point of the solvent because of the autogenous pressure.
All solutions were pre-heated before measurements. For all samples, the LD contribution was negligible (ΔLD < 0.005 dOD) and the shape of the CD signal was independent of the orientation of the quartz cells. Molar CD values are reported in L·mol^{-1}·cm^{-1} and are expressed as follows: $\Delta\varepsilon=\theta/(32982\times l\times c)$ where θ is the measured ellipticity (mdeg), l is the optical path length in cm, and c is the **BTA P*** concentration in mol·L^{-1}.

<u>UV-Vis analyses</u>: UV-Vis absorption spectra were extracted from CD on each of the above samples and obtained after correction of the absorption of air, solvent, and cell at the same temperature.

Nuclear Magnetic Resonance (NMR) analyses: NMR spectra were recorded on a Bruker Avance 400 spectrometer. ^{1}H and $^{31}P\{^{1}H\}$ NMR spectra for the 16.9 mM solution of **BTA P*** in toluene-d_8 were recorded upon cooling from 373 K to 253 K with full spectra at 373 K, 323 K, 303 K, 293 K, 283 K, 273 K, 263 K and 253 K (Figure S5).

Isothermal Titration Calorimetry (ITC) analyses: ITC data were recorded on a Microcal VP-ITC apparatus at the desired temperature, injecting a toluene solution containing **BTA P*** (Figure 1b) into pure toluene. Injections of 5 µL over 10 seconds were performed every 300 seconds at a stirring rate of 260 rpm.

Viscosimetry: Viscosimetry measurements were performed on an Anton Paar AMVn falling-ball microviscosimeter with a Ø 0.16 mm capillary, between 278 K and 353 K (upon heating, every 5K), for solutions of **BTA P*** in either toluene or fluorobenzene. The temperature was stabilized for 5 min before starting the measurement. At each temperature, measurements were done at an angle of +70° and -70° (5 times for each angle, 10 measurements in total). Results are reported as an average of these 10 measurements. Toluene and fluorobenzene was recorded under the same conditions and the relative viscosities reported in Figure 6b correspond to the ratio between the falling times of **BTA P*** solutions and the corresponding solvent. The same protocol was followed to measure the relative viscosities of solutions of **BTA P** and **BTA*** in toluene (Figure S16e).

Small-Angle Neutron Scattering (SANS) analyses: SANS measurements were made at the LLB (Saclay, France) on the PA20 instrument, at three distance-wavelength combinations to cover the 2×10^{-3} to 0.3 $Å^{-1}$ q-range, where the scattering vector q is defined as usual, assuming elastic scattering, as $q=(4\pi/\lambda)\sin(\theta/2)$, where θ is the angle between incident and scattered beam. Data were corrected for the empty cell signal and the solute and solvent incoherent background. A light water standard was used to normalize the scattered intensities to cm^{-1} units. The data was fitted with the DANSE software SasView. The number n of molecules in the cross-section can be derived from n_L (the number of molecules per unit length)[7] by assuming an average intermolecular distance of 3.62 Å, which is the usual spacing between aromatic rings in BTA helical assemblies.

Small-Angle X-ray Scattering (SAXS) analyses: SAXS measurements were made at Orsay on a home-made instrument equipped with a closed-cycle helium cryogenerator (minimum temperature, ca. 30K). The X-ray generator is a Rigaku-MicroMax (focus size 70x70 $µm^2$; 40 kV, 30 mA) and a multilayer graded optics delivers a parallel beam 1 mm^2 in cross-section. The presently chosen sample-to-detector distance offers a q-range of analysis between 0.02 and 0.17 $Å^{-1}$. The data was fitted with the DANSE software SasView to get the radius of the rodlike assemblies at 293 K and 193 K.

High-sensitivity differential scanning calorimetry (DSC) analyses: Solution-phase differential scanning calorimetry measurements were performed on a high sensitivity

TA Instruments nDSC III system (baseline noise ± 15 nW), between 278 K and 323 K, using 3 full heating/cooling cycles, at 0.2 K.min^{-1}. The reference cell was filled with the solvent and the sample cell (0.3 mL) with the solution of **BTA P***. The capillary cells were not capped, and a constant pressure of 5×10^5 Pa was applied.

Molecular modelling: A hexadecamer of an analogue of **BTA P*** with (*S*) sec-butyl side chains and a specific arrangement of the diphenylphosphino unit (right-handed) relatively to the hydrogen-bonding network (left-handed) was built and modelled with the Materials Studio 6.0 modelling package from Biovia[BIOVIA, Dassault Systemes, Biovia Materials Studio, 6.0, San Diego: Dassault Systemes, 2011]. As a forcefield, Dreiding[8] was selected, and modified in house to better describe the van der Waals interactions of hydrogen atoms[9] and the torsions involving the BTA substituents. The charges on the atoms are ESP charges extracted from three molecules that are representative of the different fragments of the **BTA P*** analogue, optimized at the MP2/cc-pvdz level with Gaussian16 A long-range interaction cutoff was set to 15.5 Å with a spline width of 1 Å, and the dielectric constant was distance-dependent. The hexadecamer was submitted to molecular mechanics (MM) energy minimizations using the Smart Minimizer algorithm until a convergence criterion of 0.0001 kcal.mol^{-1} and 0.005 kcal per mol.Å was reached. Then, a 500 ns molecular dynamics (MD) was performed in NVT conditions using a Nose thermostat[10] to maintain the temperature at 298 K. The equations of motion were integrated with the Verlet algorithm[11], using a timestep of 1 fs, and saving structures every 250 ps. The snapshot of Fig. 8b has been extracted at the end of the MD.

Absorption and CD spectra have then been modelled for the assembly. The last structure obtained by MD has been further optimized by MM, then reduced to eight monomers to limit calculation time, by removing four monomers at both extremities of the stack. Gaussian 16 has been used to perform TD-DFT calculations using the B3LYP functional and the 6-31G(d,p) basis set to determine the excitation energies and exciton couplings for the individual chromophores. EXAT[12] was used to build the excitonic Hamiltonian, using the first eight excited states per monomer. The intensities of the absorption and CD spectra were convoluted with a gaussian function of HWHM of 3000 cm^{-1}.

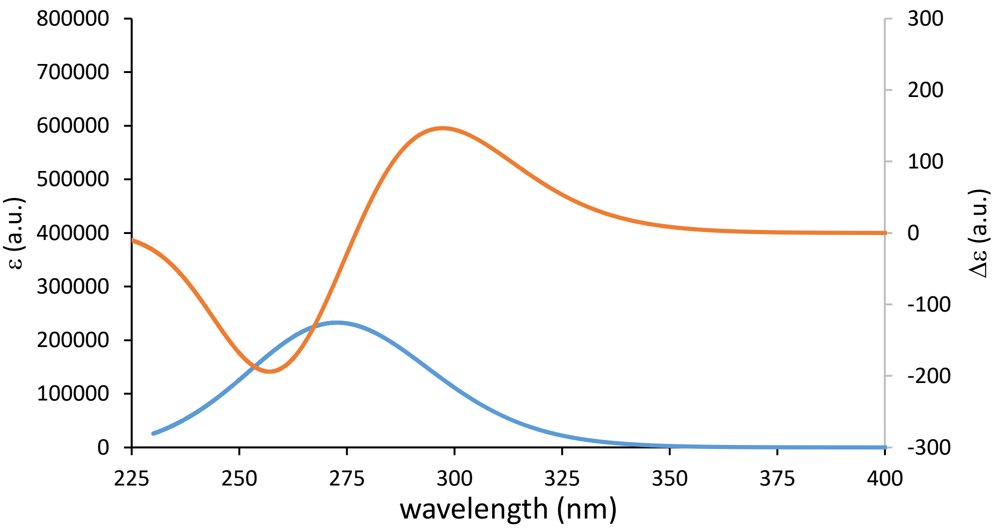

Figure S21: Modelled absorption and CD spectra of an analogue of **BTA P*** with (*S*) sec-butyl side chains and a specific arrangement of the diphenylphosphino unit (right-handed) relatively to the hydrogen-bonding network (left-handed). Only the low-energy part of the spectra has been reproduced, as only the first eight excited states per chromophore were considered.

Atomic Force Microscopy (AFM): Dry films of **BTA P*** were prepared by spin-coating of toluene and fluorobenzene solutions onto silica wafer at 293 K and characterized using AFM at the air/silica interface. The samples were imaged using tapping mode AFM on a Cypher AFM (Oxford Instruments) with AC240TS probe (spring constant: k = 2 N/m, resonant frequency: f = 70 kHz). Images were acquired with a scan size varying from 500 nm to 5 µm with a resolution of 512 × 512 pixels and line rate 1 Hz. Images were taken at different locations on each sample, with each location separated by at least a few micrometers. The images were analyzed using Scanning Probe Image Processor (SPIP) software.

Catalytic experiments (Tables S4-S6)

For all catalytic experiments a pre-catalytic mixture composed of the ligand, the copper salt and the substrate was prepared as follows: oven-dried test tubes were loaded with a given amount of a stock solution prepared by mixing **BTA P*** and [$Cu(OAc)_2 \cdot H_2O$], divided in order to get **BTA P*** (7.02 mg, 10.14 μmol, 6.0 mol%) and [$Cu(OAc)_2 \cdot H_2O$] (1.01 mg, 5.07 μmol, 3.0 mol%) in dry THF (500 μL) in each tube. The mixture was stirred for 30 minutes at 293 K, then the solvent was removed after the tubes were kept under vacuum (10^{-3} mbar) for 1 hour. Then 1-(4-nitrophenyl)ethanone (**NPnone**, 27.89 mg, 169 μmol, 100 mol%) was added. Finally, 600 μL of either dry toluene, fluorobenzene or a mixture of toluene and hexafluorobenzene (9/1, volume ratio) was added to each vial. The mixtures were stirred for 15 min at 293 K and then briefly heated up to solvents boiling point (≈ 383 K). The mixtures were stirred for another 15 min to cool down the mixtures to 293 K. Then the tubes were transferred into a bath with controlled temperature, two tubes per temperature for duplicating the experiments. $PhSiH_3$ (41.7 μL, 388 μmol, 100 mol%) was added to each test tube when arrived the desired temperature and the mixtures were stirred for 17 hours at the desired temperature.

Typical work-up: Aqueous solution of HCl (10 wt%, 1 mL) was added at the temperature of the catalytic experiment and the mixtures were stirred for 30 min (until the solution became transparent). The tubes were removed from the temperature-controlled batch and let reach 293 K with stirring. Then, the products were extracted with Et_2O (1 mL) and AcOEt (1 mL) for three times and the organic phase was passed through a small silica plug. The solvents were evaporated and the crude material was analyzed by NMR and by chiral GC. Conversion >99% was obtained for all catalytic experiments, as determined by GC and ^{1}H NMR analyses. The optical purity was determined by GC analysis.

Chiral GC analyses: The optical purity was determined by GC analysis: Chiral Cyclosil-B column, 30 m × 250 μm x 0.25 μm, inlet pressure= 12.6 psi. Injection temperature= 250°C; detector temperature= 300°C; column temperature= 145°C. Variation in the retention times of the enantiomers is due to the aging of the column; **(*R*)-NPnol** and **(*S*)-NPnol** are the first- and second-eluted enantiomers, respectively.[13] Representative GC analyses are given in Figures S29-S39.

Table S4 Enantioselectivities in NPnol for **BTA P*** solutions in toluene at different temperatures, **BTA P*=** 16.9 mM. Most experiments have been done in duplicate as indicated. Conversion> 99%.

Entry	T (°C)	T (K)	1000/ T (K^{-1})	ee±1% in **NPnol** (%)	average ee±1% in **NPnol** (%)	average ln (*S*/*R*)
1	77	350	2.86	21.4, 21.3	21.4	0.434
2	57	330	3.03	29.7, 29.8	29.8	0.614
3	37	310	3.22	36.4, 36.0	36.2	0.758
4	28	301	3.32	40.0, 38.2	39.1	0.826
5	22	295	3.39	40.2	40.2	0.852
6	17	290	3.45	41.8, 41.9	41.8	0.892
7	12	285	3.51	43.7, 43.7	43.7	0.937
8	7	280	3.57	48.5, 48.5	48.5	1.059
9	2	275	3.63	46.0, 46.6	46.3	1.002
10	-3	270	3.70	50.2, 51.8	51.0	1.126
11	-8	265	3.77	49.0, 49.7	49.4	1.083
12	-13	260	3.84	51.5, 50.5	51.0	1.125
13	-20	253	3.95	51.2	51.2	1.131
14	-23	250	4.00	49.5, 50.5	50.0	1.099
15	-30	243	4.11	51.6	51.6	1.142
16	-33	240	4.16	52.9, 51.2	52.0	1.154

Table S5 Enantioselectivities in **NPnol** for **BTA P*** solutions in fluorobenzene at different temperatures, **BTA P*=** 16.9 mM. Most experiments have been done in duplicate as indicated. Conversion> 99%.

Entry	T (°C)	T (K)	1000/ T (K^{-1})	ee±1% in **NPnol** (%)	average ee±1% in **NPnol** (%)	average ln (*S*/*R*)
1	57	330	3.03	22.9, 23.8	23.4	0.476
2	52	325	3.08	24.5, 24.6	24.5	0.501
3	46	319	3.13	28.7, 29.7	29.2	0.602
4	42	315	3.17	30.6, 30.6	30.6	0.632
5	37	310	3.22	32.3, 32.0	32.2	0.667
6	33	306	3.27	32.6	32.6	0.677
7	28	301	3.21	36.2, 35.3	35.8	0.748
8	22	295	3.39	36.6, 36.6	36.6	0.768
9	18	291	3.43	37.1, 36.7	36.9	0.775
10	12	285	3.51	37.4, 39.6	38.5	0.812
11	5	278	3.60	41.0, 40.0	40.5	0.859

12	-2	271	3.69	43.6, 42.4	43.0	0.919
13	-9	264	3.79	44.9, 44.2	44.6	0.958
14	-20	253	3.95	47.0, 46.6	46.8	1.015
15	-30	243	4.11	47.7, 46.8	47.2	1.027

Table S6 Enantioselectivities in **NPnol** for **BTA P*** solutions in a mixture of toluene and hexafluorobenzene (9/1, volume ratio) at different temperatures, **BTA P*=** 16.9 mM. Most experiments have been done in duplicate as indicated. Conversion> 99%.

Entry	T (°C)	T (K)	1000/ T (K^{-1})	ee±1% in **NPnol** (%)	average ee±1% in **NPnol** (%)	average ln (*S*/*R*)
1	54	327	3.01	29.6, 28.8	29.2	0.602
2	46	319	3.13	33.4, 29.8	31.6	0.677
3	37	310	3.22	36.3, 35.1	35.7	0.747
4	28	301	3.32	35.7	35.7	0.747
5	22	295	3.39	38.2, 40.2	39.2	0.828
6	18	291	3.43	39.6, 39.6	39.6	0.838
7	12	285	3.51	41.5	41.5	0.883
8	5	278	3.6	41.6, 43.1	42.4	0.905
9	-2	271	3.69	46.7, 43.1	44.9	1.005
10	-5	268	3.73	41.2, 43.0	42.1	0.899
11	-9	264	3.79	42.8, 43.1	43.0	0.919
12	-10	263	3.8	43.3, 41.6	42.4	0.906
13	-15	258	3.87	44.8, 43.7	44.2	0.951
14	-20	253	3.95	47.7, 48.1	47.9	1.044
15	-25	248	4.03	45.4, 47.6	46.5	1.007
16	-30	243	4.11	51.7, 50.2	51.0	1.124

Synthesis of BTA P* (Figures S22-S25)

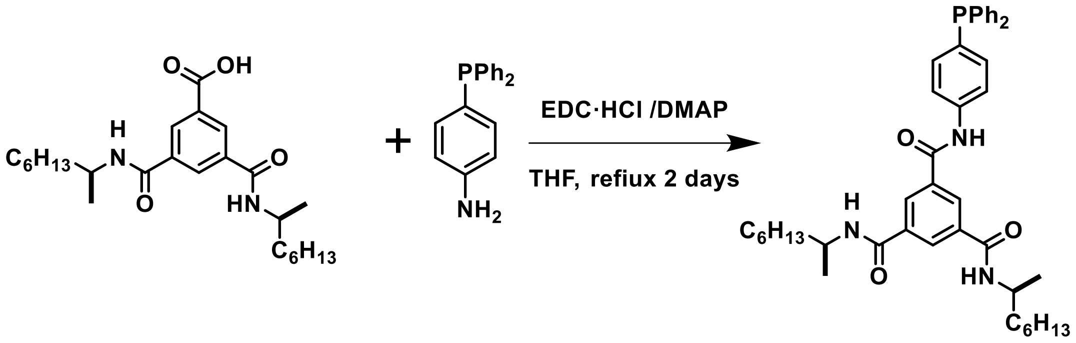

1-carboxylic acid-benzene-3,5-di-(1*S*)-methylheptyl amide (666 mg, 1.54 mmol, 1 equiv.), 4-diphenylphosphinoaniline[14] (641 mg, 2.31 mmol, 1.5 equiv.), EDC·HCl (502 mg, 2.62 mmol, 1.7 equiv.) and DMAP (320 mg, 2.62 mmol, 1.7 equiv.) were dissolved in THF (40 mL) in a one-necked flask. The resulting mixture was stirred and refluxed for two days under argon. After cooling down to the room temperature, the solvent was evaporated under reduced pressure. The product was purified by flash column chromatography over silica gel, eluting with DCM/EtOAc (93/7) yielding **BTA P*** as a colourless solid (727 mg, 68% yield). The **BTA P*** solid was stored under argon to avoid oxidation. The enantiomer of **BTA P***, **BTA (*R*,*R*)-P***, was synthesized in the same way from 1-carboxylic acid-benzene-3,5-di-(1*R*)-methylheptyl amide to determine the optical purity of **BTA P***. **^{1}H NMR** (400 MHz, DMSO-d$_6$), δ (ppm): 10.65 (s, 1H, ArNH), 8.47-8.42 (m, 5H, CHN*H* + BTA ring), 7.85 (dd, *J* = 8.7, 1.2 Hz, 2H, CH$_{arom.}$ linker), 7.44-7.38 (m, 6H, PAr$_2$), 7.30- 7.22 (m, 6H, PAr$_2$ + CH$_{arom.}$ linker), 4.08-3.99 (m, 2H, C*H*NH), 1.64-1.43 (m, 4H, C*H*$_2$CHNH), 1.42-1.22 (m, 16H, CH$_2$), 1.15 (d, *J* = 6.6 Hz, 6H, CHC*H*$_3$), 0.87-0.82 (m, 6H, CH$_3$**)**. **^{31}P{^{1}H} NMR** (162 MHz, DMSO-d$_6$), δ (ppm): −7.8. **^{13}C{^{1}H} NMR** (101 MHz, DMSO-d$_6$), δ (ppm): 165.2, 164.7 (d, *J* = Hz), 139.9, 137.0 (d, *J* = 11.4 Hz), 135.4 (d, *J* = Hz), 135.2, 134.1 (d, *J* = 20.6 Hz), 133.0 (d, *J* = 19.4 Hz), 131.4, 128.7 (q, *J* = 6.8 Hz), 120.4 (d, *J* = 7.4 Hz), 45.2, 39.5 (below the solvent peak), 35.9, 31.2, 28.6, 25.8, 22.0, 20.7, 13.9. **HRMS** (ESI, m/z) for $C_{43}H_{54}N_3O_4PNa$ [M+O+Na]$^+$: calculated 730.3744, found: 730.3741. Oxidation of the ligand presumably occurs during the ionization process (see Figure S23). **FT-IR** (solid, cm^{-1}): 1557 (m, C-N), 1593 (m, C-N), 1633 (s, C=O), 3234 (m br, N-H stretch), 3360 (w, N-H stretch). Enantiomeric excess > 99% (determined by chiral HPLC, see Figure S26). It was verified that no significant oxidation occurs upon preparation of the solutions (see Figures S25-26).

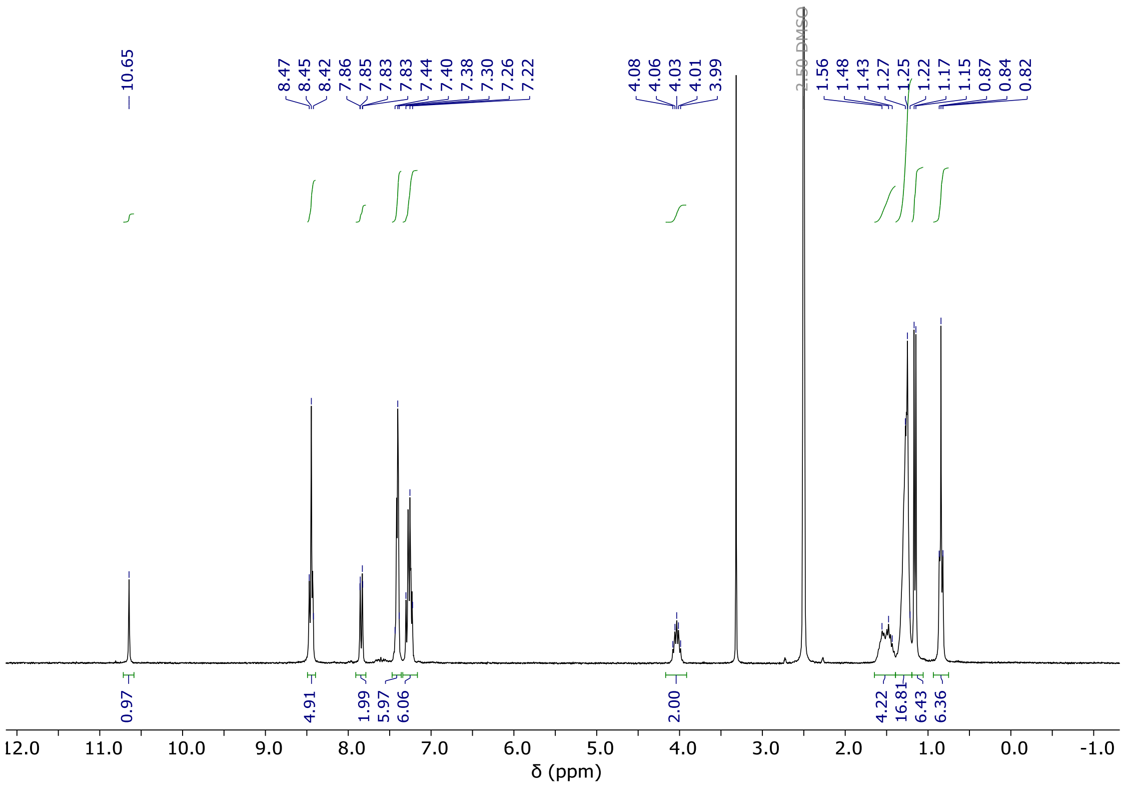

Figure S22. ^{1}H NMR of **BTA P*** in DMSO-d6. Signal at *ca.* 3.3 ppm is water.

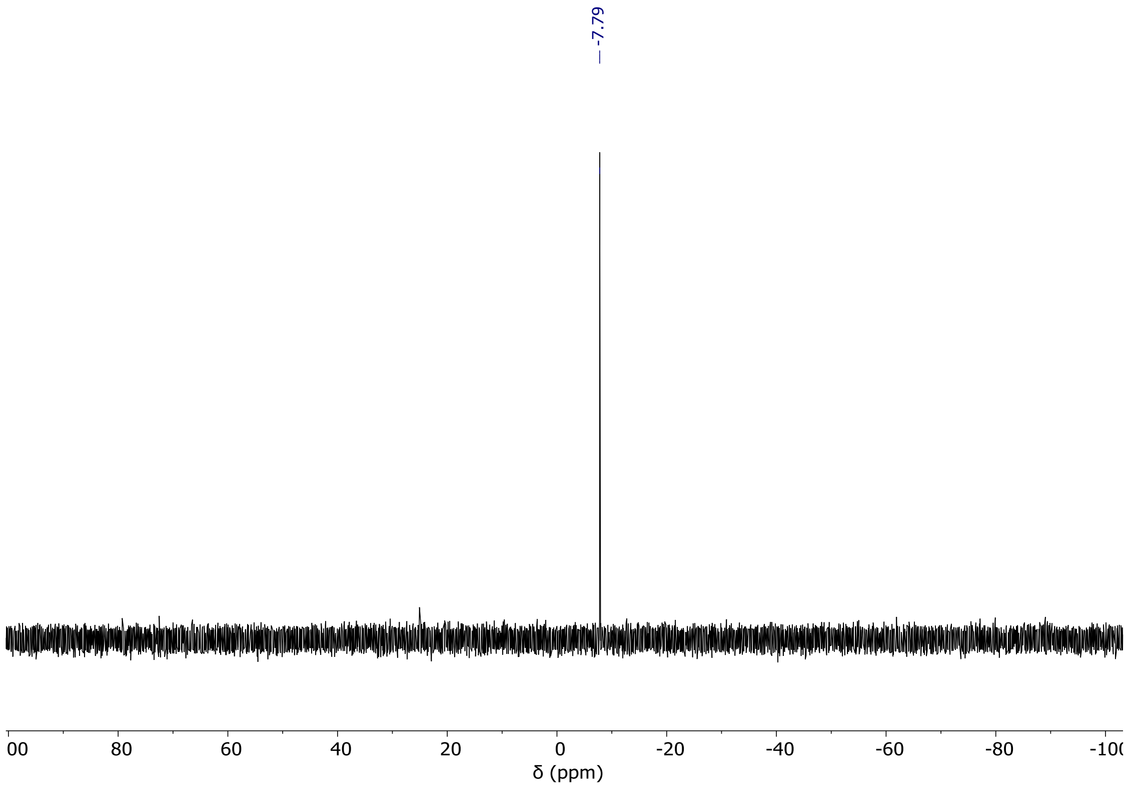

Figure S23. ^{31}P{^{1}H} NMR of **BTA P*** in DMSO-d6. The tiny signal δ= 25.1 ppm is due some oxidation of **BTA P*** which occurs in DMSO-d6 prior and during NMR recording.

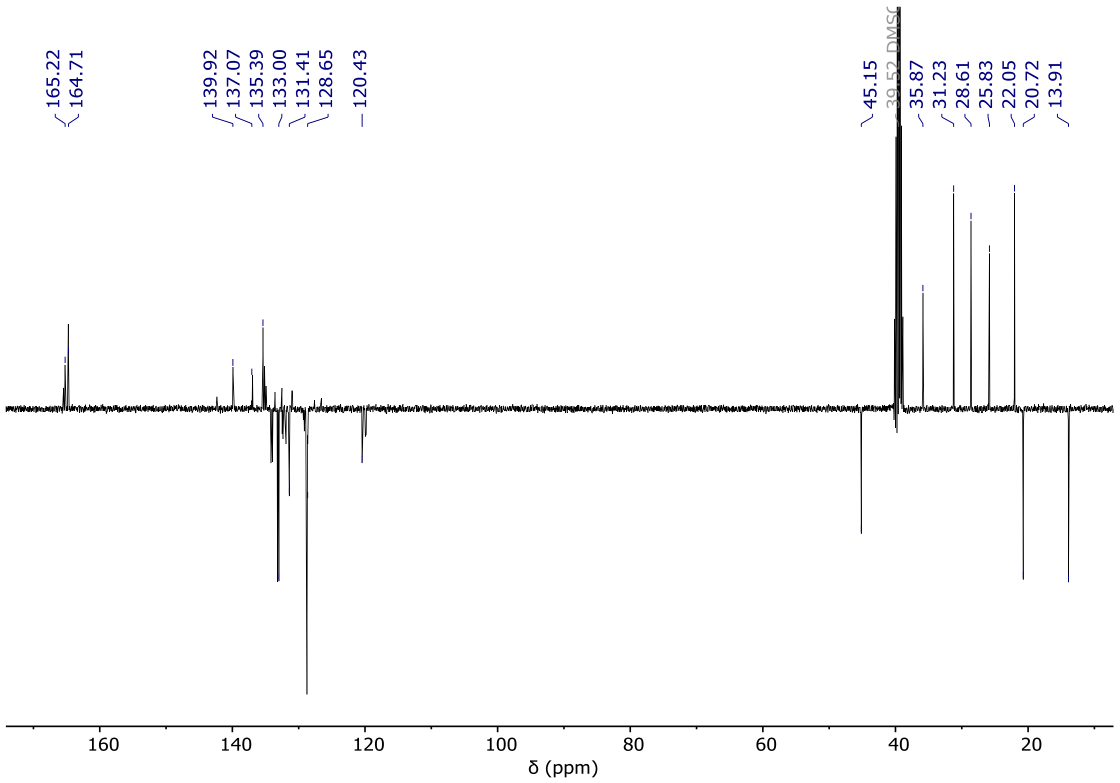

Figure S24. ^{13}C JMOD-NMR of **BTA P*** in DMSO-d_6.

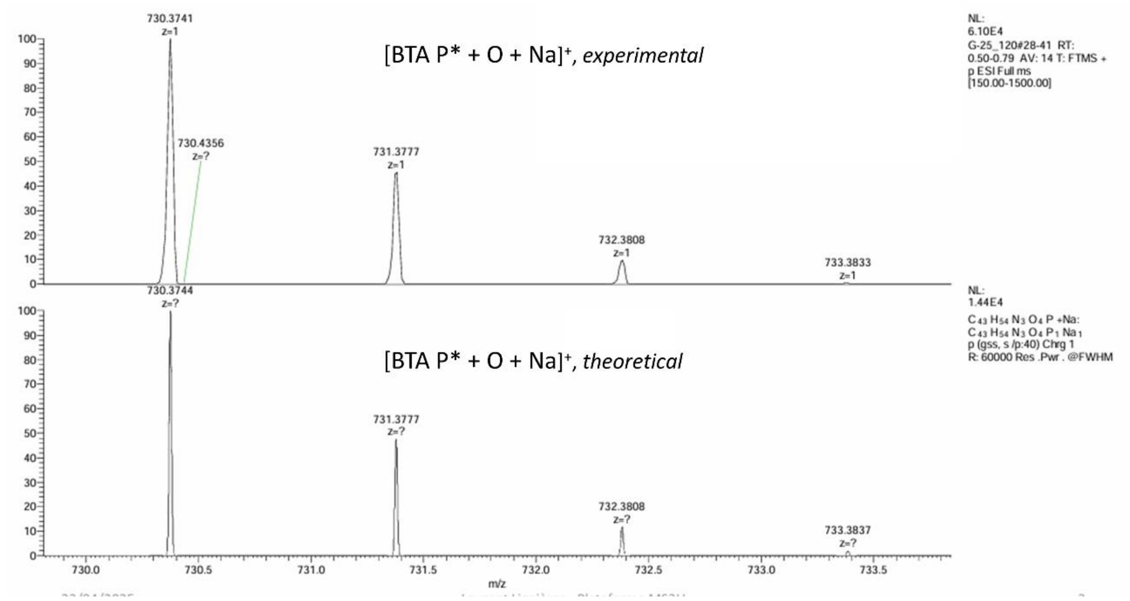

Figure S25. HRMS of **BTA P*** in methanol.

Optical purity of BTA P* (Figure S26)

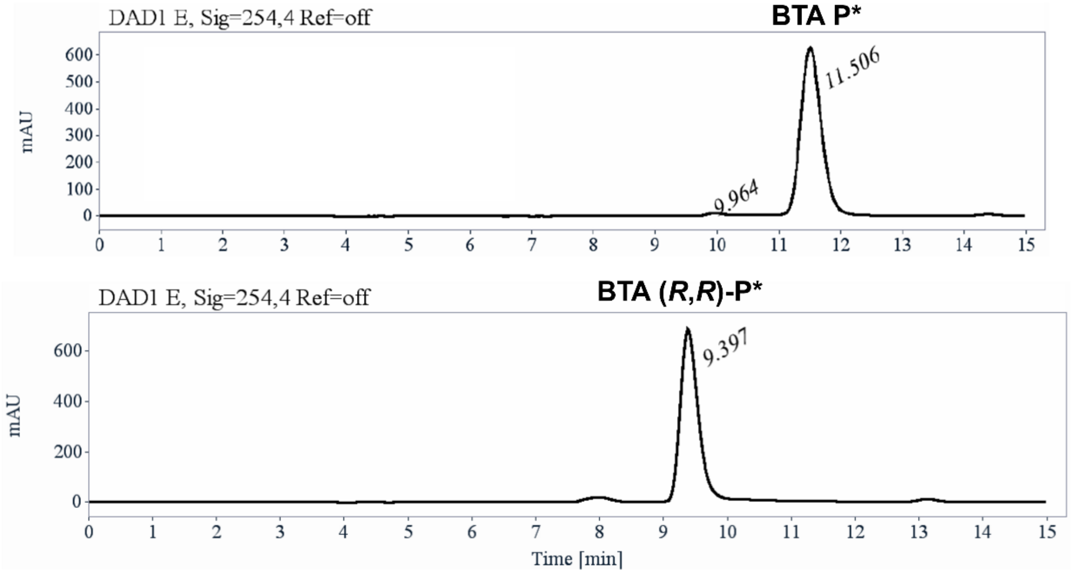

Figure S26. Chiral HPLC analyses of **BTA P*** and **BTA *(R,R*)-P***. Results: **BTA P***: > 99 ee. **BTA *(R,R)*-P***: > 99% ee.

Stability of BTA P* (Figures S27-28)

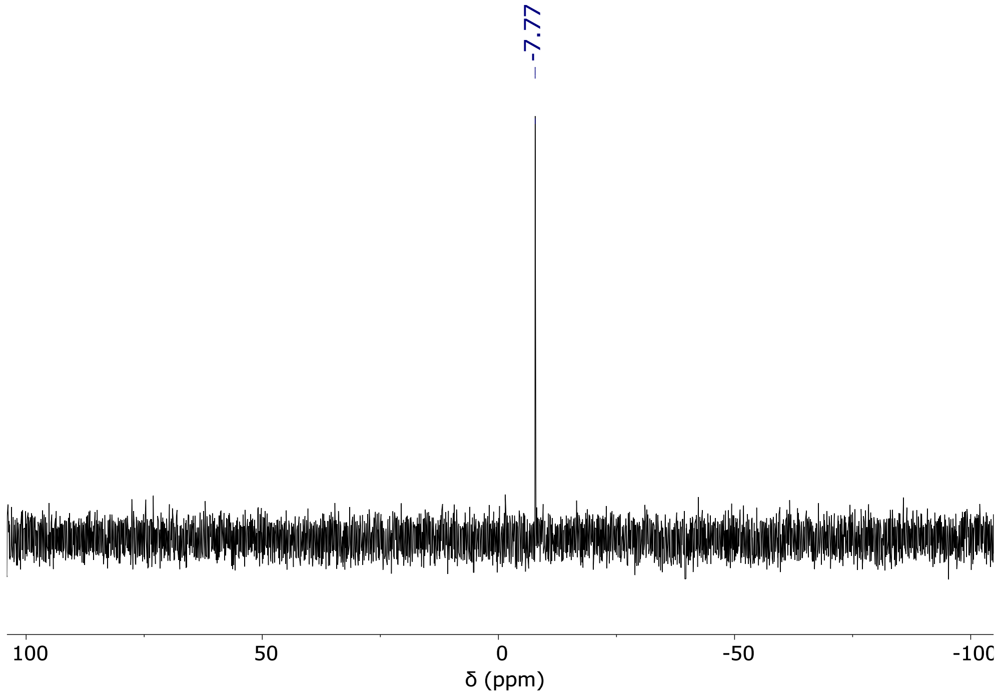

Figure S27. $^{31}P\{^1H\}$ NMR of **BTA P*** in DMSO-d_6. The solution was prepared by heating a 5.0 mM solution of **BTA P*** in *p*-xylene in an oil bath at 393 K with stirring bar for 10 minutes, cooling down to 293 K and by removing *p*-xylene under vacuum. No significant oxidation of the ligand occurred.

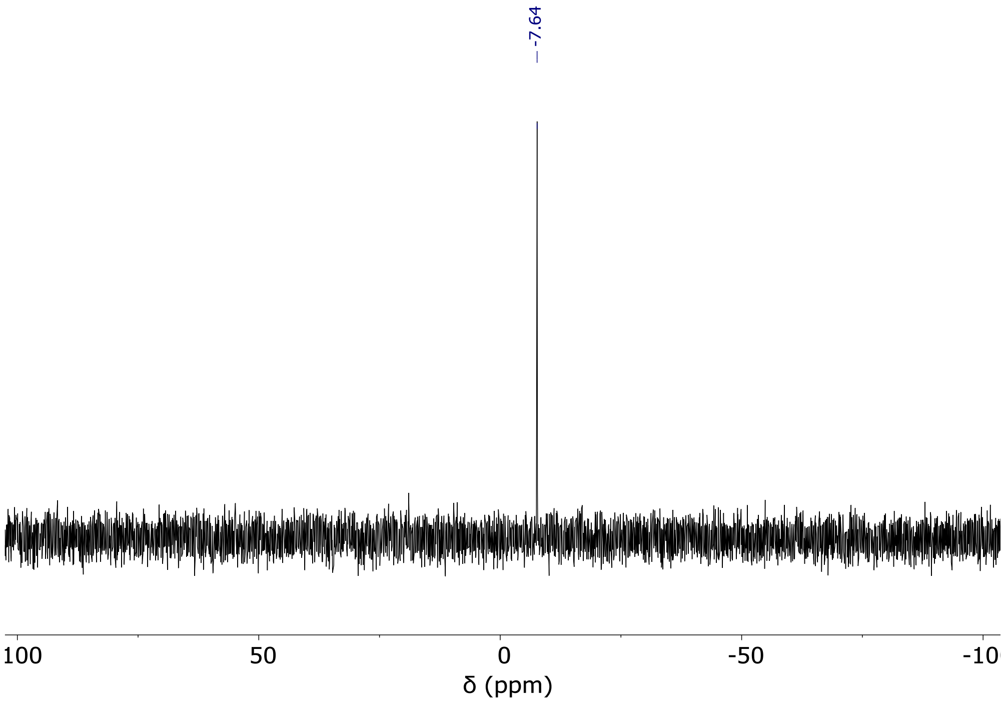

Figure S28. $^{31}P\{^1H\}$ NMR of **BTA P*** solution in DMSO-d_6. The solution was prepared by heating a 5.0 mM solution of **BTA P*** in mesitylene in an oil bath at 438 K with stirring bar for 10 minutes, cooling down to 293 K and by removing mesitylene under vacuum. No significant oxidation of the ligand occurred.

Selected chiral GC analyses (Figures S29-S39)

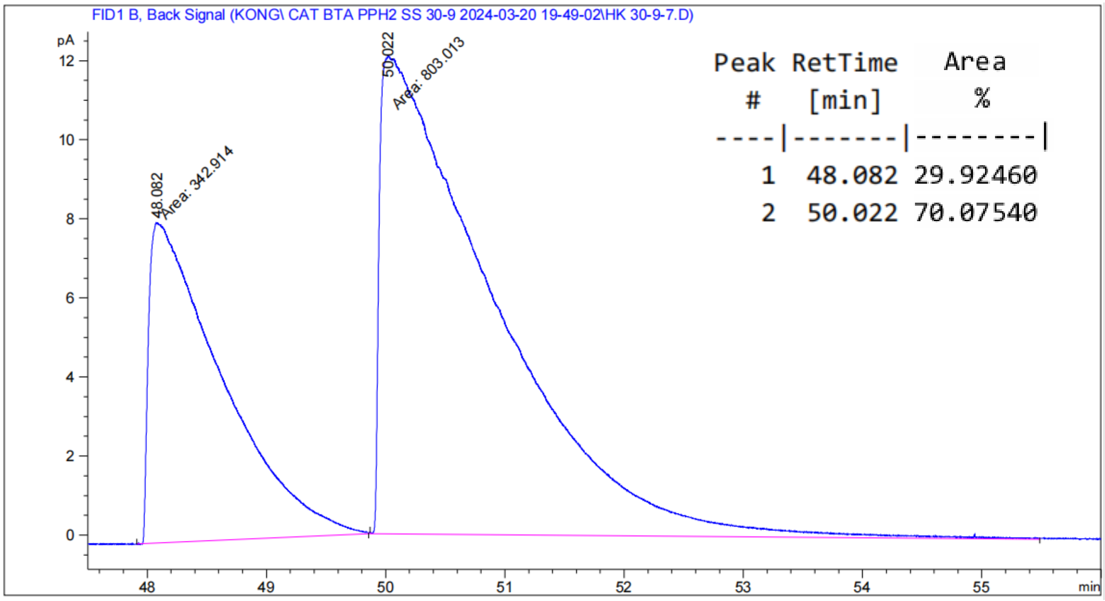

Figure S29. Chiral GC analysis of entry 5, Table S4. Solvent: toluene, 295 K, 40.2% ee.

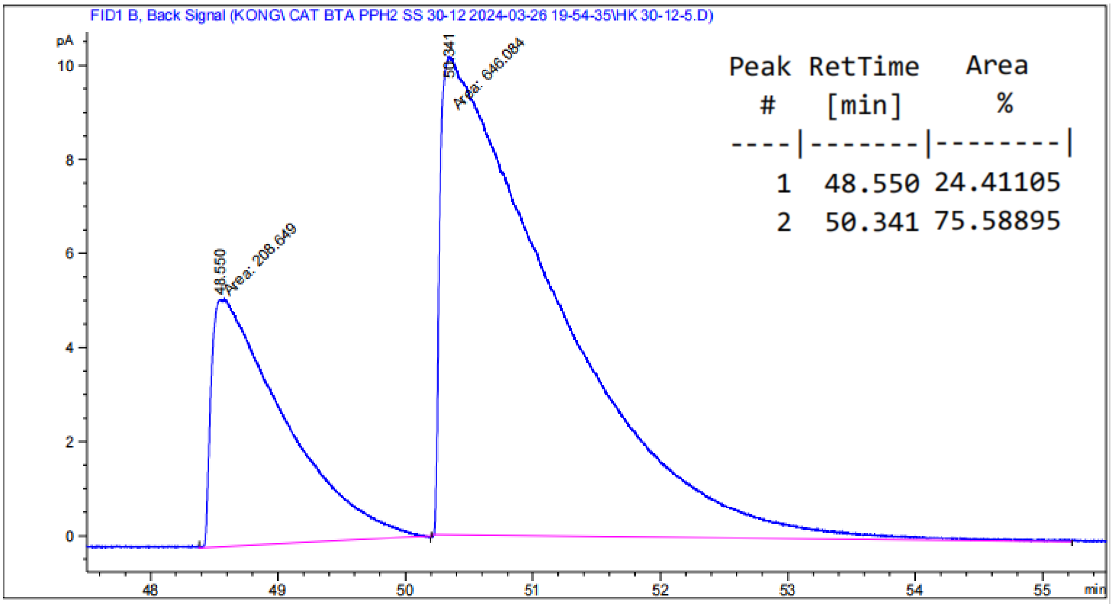

Figure S30. Chiral GC analysis of entry 13, Table S4. Solvent: toluene, 253 K, 51.2% ee.

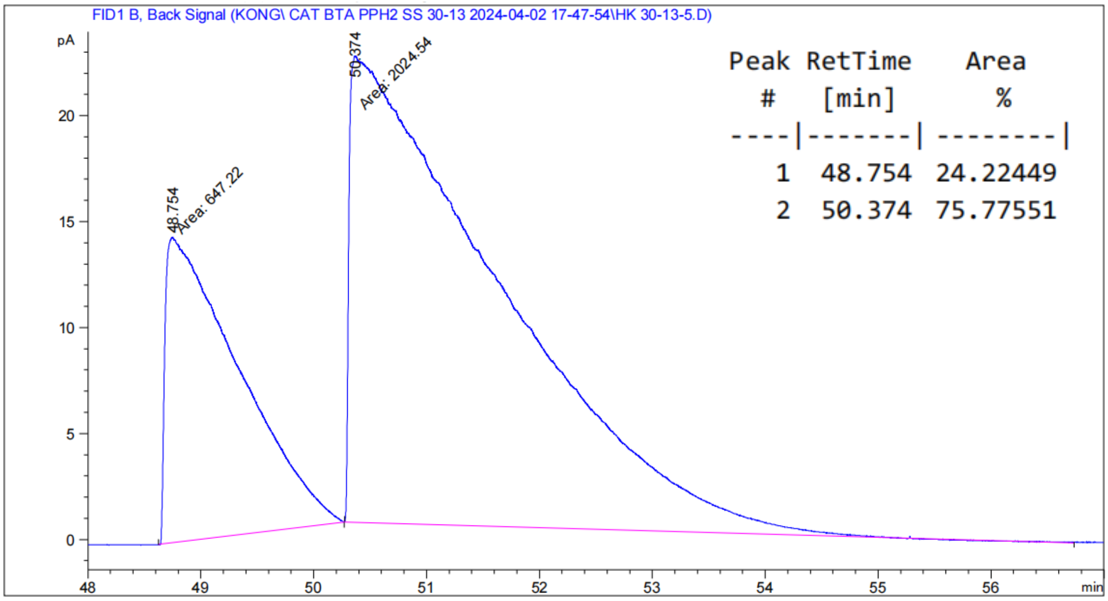

Figure S31. Chiral GC analysis of entry 15, Table S4. Solvent: toluene, 243 K, 51.6% ee.

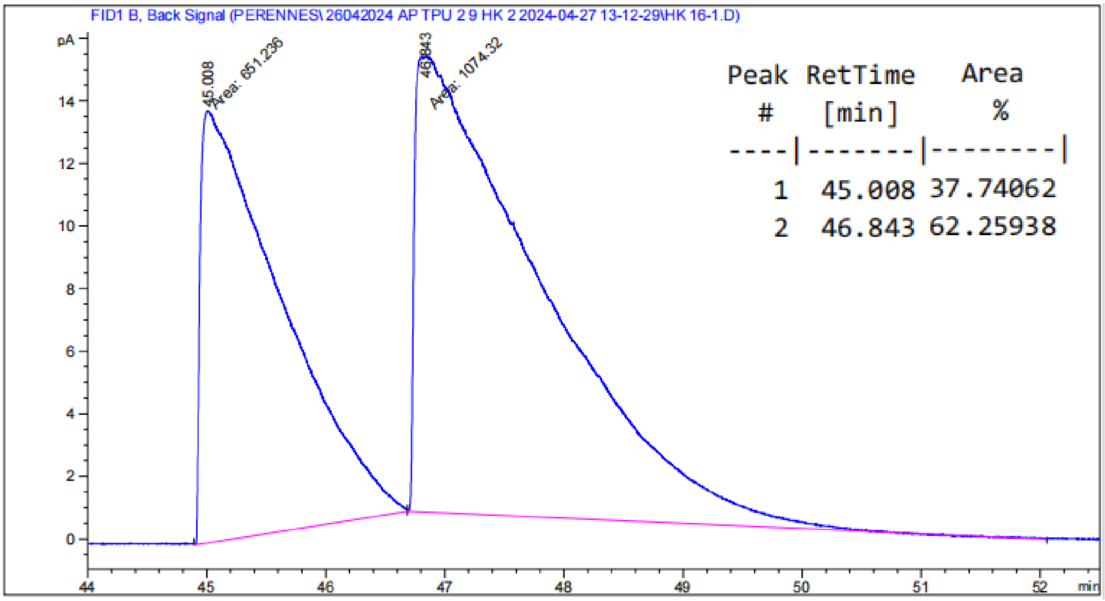

Figure S32. Chiral GC analysis of entry 2, Table S5. Solvent: fluorobenzene, 325 K, 24.5 % ee.

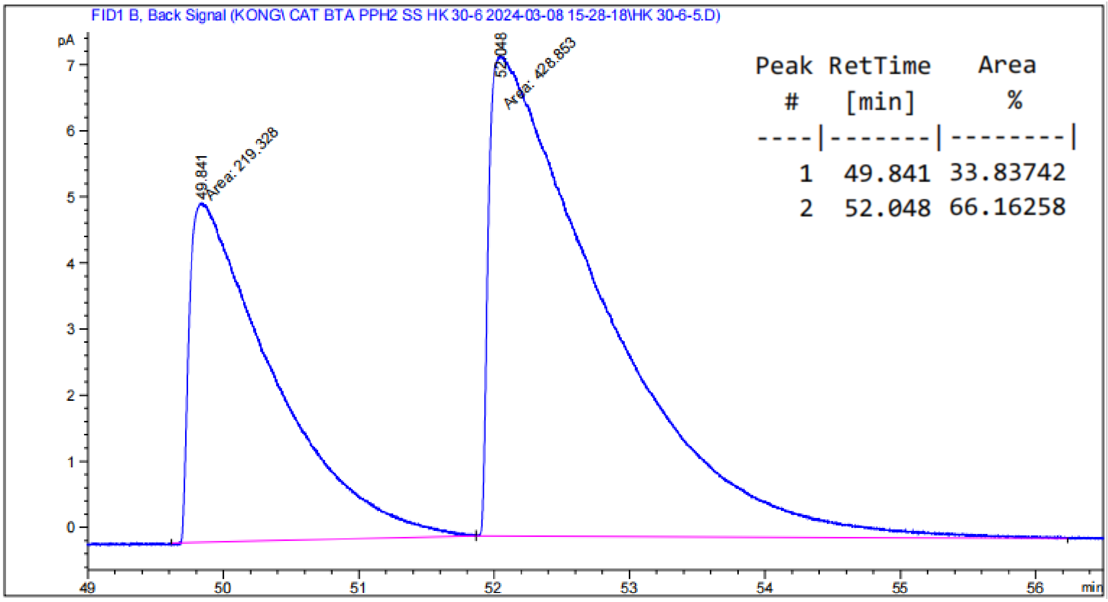

Figure S33. Chiral GC analysis of entry 5, Table S5. Solvent: fluorobenzene, 310 K, 32.3 % ee.

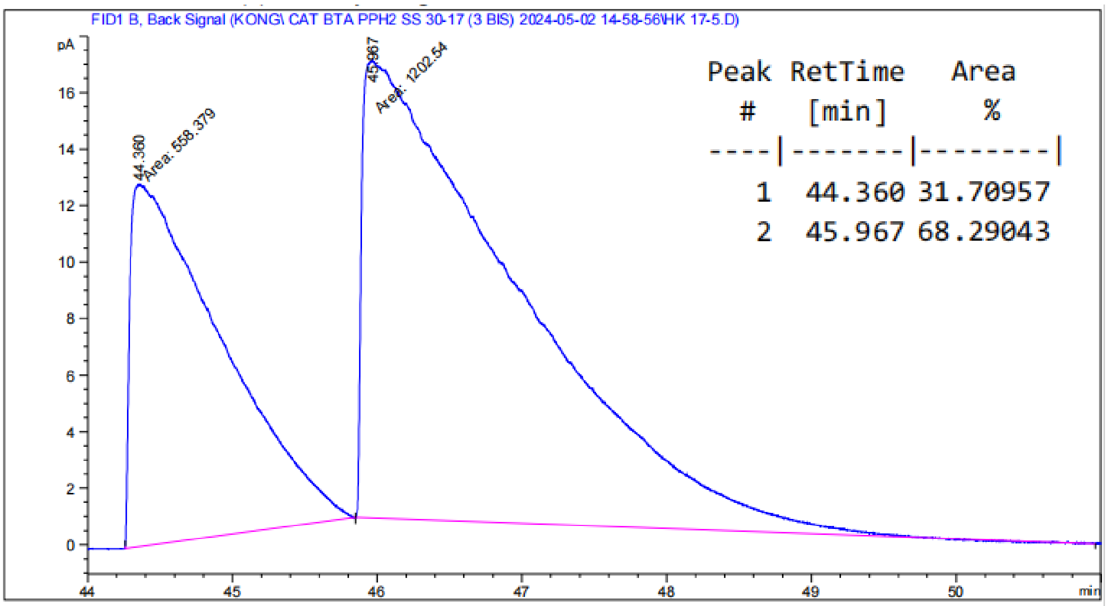

Figure S34. Chiral GC analysis of entry 8, Table S5. Solvent: fluorobenzene, 295 K, 36.6 % ee.

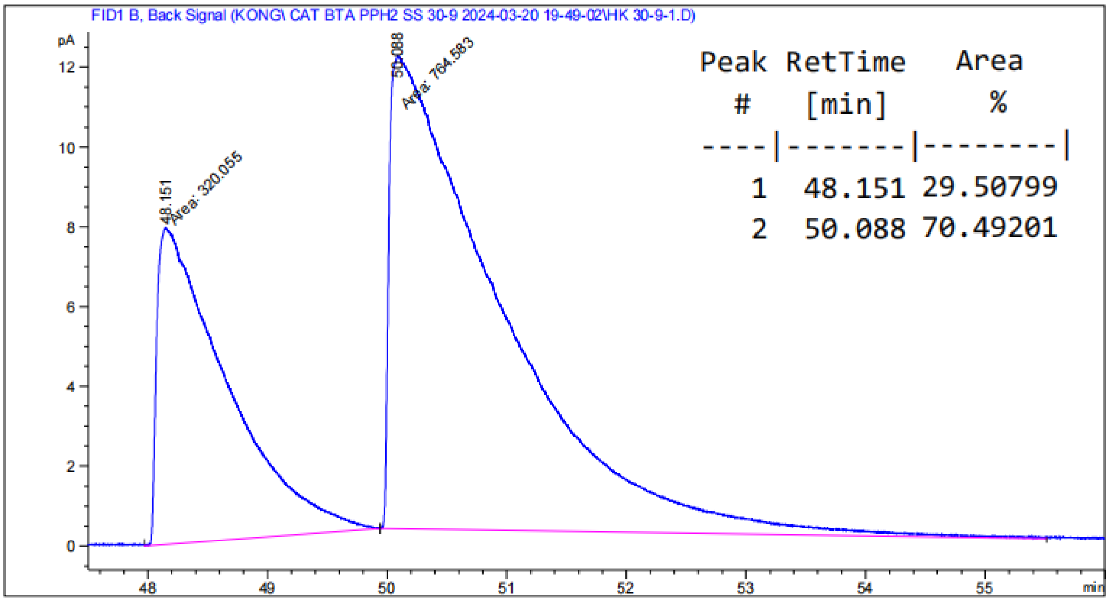

Figure S35. Chiral GC analysis of entry 11, Table S5. Solvent: fluorobenzene, 278 K, 41.0 % ee.

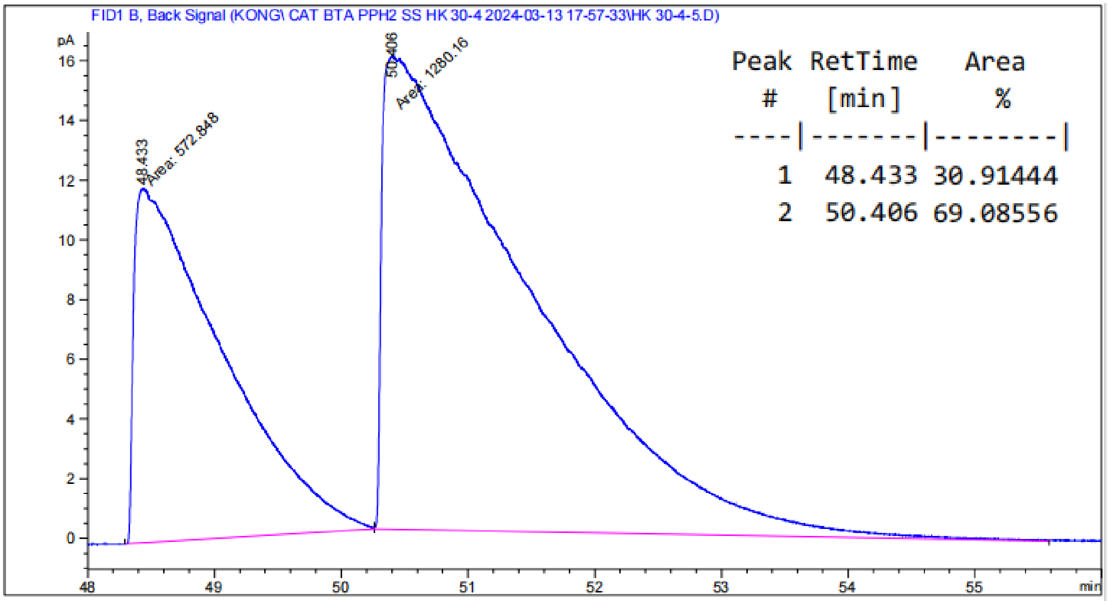

Figure S36. Chiral GC analysis of entry 5, Table S6. Solvent: mixture of toluene and hexafluorobenzene (9/1, vol%), 295 K, 38.2 % ee.

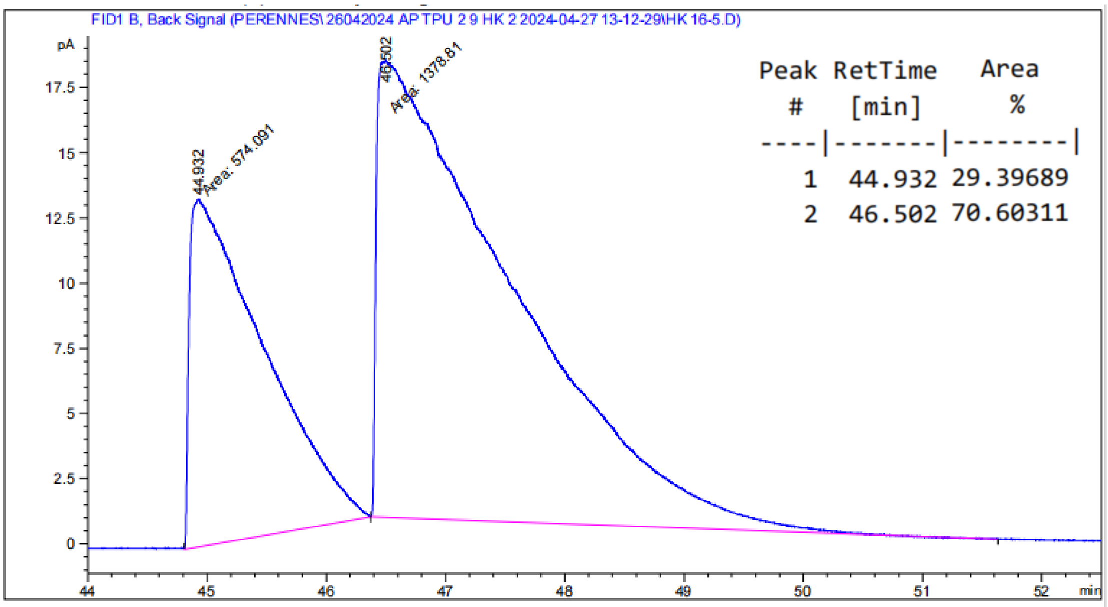

Figure S37. Chiral GC analysis of entry 10, Table S6. Solvent: mixture of toluene and hexafluorobenzene (9/1, vol%), 268 K, 41.2 % ee.

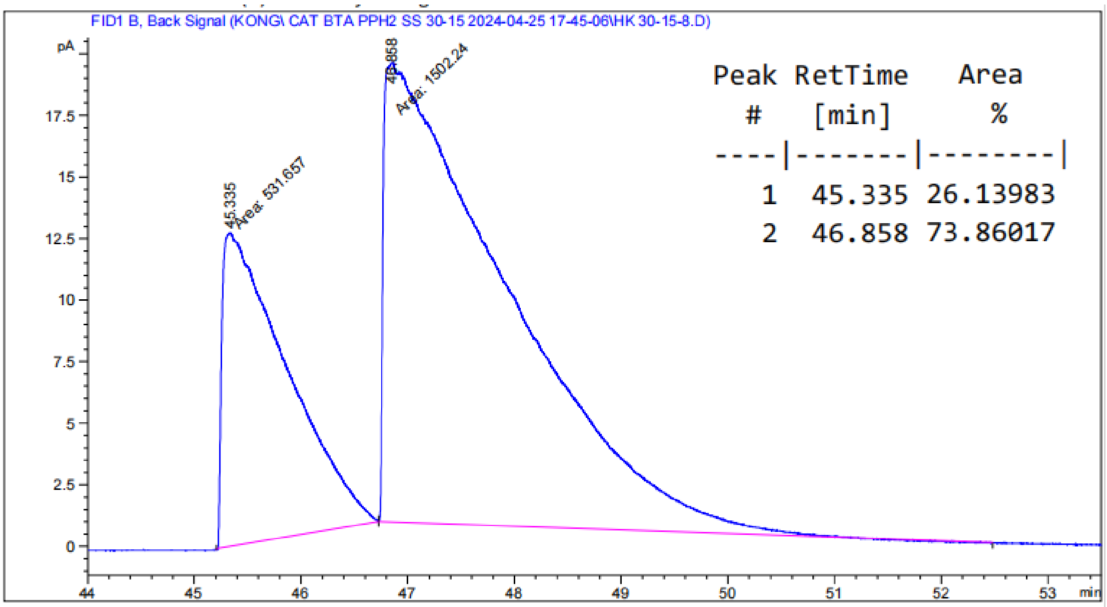

Figure S38. Chiral GC analysis of entry 14, Table S6. Solvent: mixture of toluene and hexafluorobenzene (9/1, vol%), 253 K, 47.7 % ee.

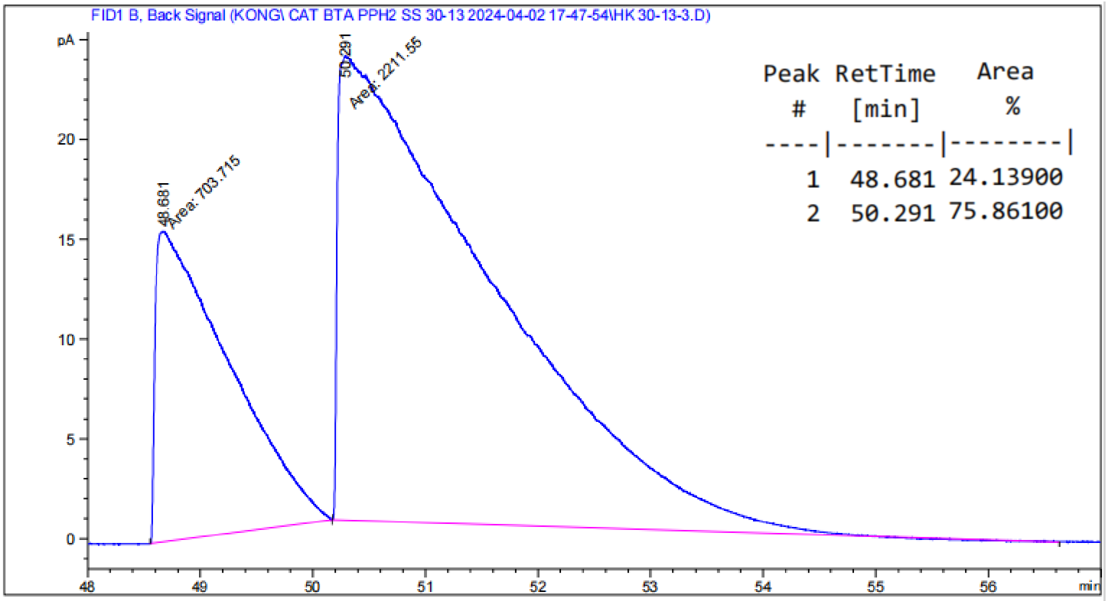

Figure S39. Chiral GC analysis of entry 16, Table S6. Solvent: mixture of toluene and hexafluorobenzene (9/1, vol%), 243 K, 51.7 % ee.

References

[1] S. Spange, N. Weiß, C. H. Schmidt, K. Schreiter, “Reappraisal of Empirical Solvent Polarity Scales for Organic Solvents” *Chemistry–Methods* **2021**, *1*, 42–60.

[2] C. Reichardt, “Solvatochromic Dyes as Solvent Polarity Indicators” *Chem. Rev.* **1994**, *94*, 2319–2358.

[3] A. Desmarchelier, B. G. Alvarenga, X. Caumes, L. Dubreucq, C. Troufflard, M. Tessier, N. Vanthuyne, J. Idé, T. Maistriaux, D. Beljonne, P. Brocorens, R. Lazzaroni, M. Raynal, L. Bouteiller, “Tuning the nature and stability of self-assemblies formed by ester benzene 1,3,5-tricarboxamides: the crucial role played by the substituents” *Soft Matter* **2016**, *12*, 7824–7838.

[4] C. M. Hansen, *Hansen Solubility Parameters: A User’s Handbook, Second Edition*, CRC Press, Boca Raton, **2007**.

[5] J. M. Zimbron, X. Caumes, Y. Li, C. M. Thomas, M. Raynal, L. Bouteiller, “Real-Time Control of the Enantioselectivity of a Supramolecular Catalyst Allows Selecting the Configuration of Consecutively Formed Stereogenic Centers” *Angewandte Chemie International Edition* **2017**, *56*, 14016–14019.

[6] P. J. Stals, M. M. Smulders, R. Martín-Rapún, A. R. Palmans, E. emsp14W Meijer, “Asymmetrically Substituted Benzene-1, 3, 5-tricarboxamides: Self-Assembly and Odd–Even Effects in the Solid State and in Dilute Solution” *Chemistry-A European Journal* **2009**, *15*, 2071–2080.

[7] F. Lortie, S. Boileau, L. Bouteiller, C. Chassenieux, B. Demé, G. Ducouret, M. Jalabert, F. Lauprêtre, P. Terech, “Structural and Rheological Study of a Bis-urea Based Reversible Polymer in an Apolar Solvent” *Langmuir* **2002**, *18*, 7218–7222.

[8] S. L. Mayo, B. D. Olafson, W. A. Goddard, “DREIDING: a generic force field for molecular simulations” *J. Phys. Chem.* **1990**, *94*, 8897–8909.

[9] S. Hoyas, V. Lemaur, Q. Duez, F. Saintmont, E. Halin, J. De Winter, P. Gerbaux, J. Cornil, “PEPDROID: Development of a Generic DREIDING-Based Force Field for the Assessment of Peptoid Secondary Structures” *Advanced Theory and Simulations* **2018**, *1*, 1800089.

[10] S. Nosé, “A molecular dynamics method for simulations in the canonical ensemble” *Molecular Physics* **1984**, *52*, 255–268.

[11] L. Verlet, “Computer ‘Experiments’ on Classical Fluids. I. Thermodynamical Properties of Lennard-Jones Molecules” *Phys. Rev.* **1967**, *159*, 98–103.

[12] S. Jurinovich, L. Cupellini, C. A. Guido, B. Mennucci, “EXAT: EXcitonic analysis tool” *Journal of Computational Chemistry* **2018**, *39*, 279–286.

[13] G. Uray, W. Stampfer, W. M. F. Fabian, “Comparison of Chirasil-DEX CB as gas chromatographic and ULMO as liquid chromatographic chiral stationary phase for enantioseparation of aryl- and heteroarylcarbinols” *Journal of Chromatography A* **2003**, *992*, 151–157.

[14] D. Gelman, L. Jiang, S. L. Buchwald, “Copper-Catalyzed C−P Bond Construction via Direct Coupling of Secondary Phosphines and Phosphites with Aryl and Vinyl Halides” *Org. Lett.* **2003**, *5*, 2315–2318.
